# HDX-MS for Epitope Characterization of a Therapeutic ANTIBODY Candidate on the Calcium-Binding Protein Annexin-A1

**DOI:** 10.3390/antib10010011

**Published:** 2021-03-19

**Authors:** Marius Gramlich, Henry C. W. Hays, Scott Crichton, Philipp D. Kaiser, Anne Heine, Nicole Schneiderhan-Marra, Ulrich Rothbauer, Dieter Stoll, Sandra Maier, Anne Zeck

**Affiliations:** 1 NMI, Natural and Medical Sciences Institute at the University of Tuebingen, Markwiesenstr. 55, 72770 Reutlingen, Germany; marius.gramlich@nmi.de (M.G.); Philipp.Kaiser@nmi.de (P.D.K.); Anne.Heine@nmi.de (A.H.); Nicole.Schneiderhan@nmi.de (N.S.-M.); Ulrich.Rothbauer@nmi.de (U.R.); Dieter.Stoll@nmi.de (D.S.); Sandra.Maier@nmi.de (S.M.); 2 Medannex Ltd., 1 Lochrin Square, Fountainbridge, Edinburgh EH3 9QA, UK; henryhays@medannex.org (H.C.W.H.); scottcrichton@medannex.org (S.C.); 3 Pharmaceutical Biotechnology, Eberhard Karls University Tuebingen, Geschwister-Scholl-Platz, 72074 Tuebingen, Germany; 4 Department of Life Sciences, University of Applied Sciences Albstadt-Sigmaringen, Anton-Guentherstr. 51, 72488 Sigmaringen, Germany

**Keywords:** HDX-MS, hydrogen–deuterium exchange, mass spectrometry, proteolysis-resistant protein, ANXA1, annexin-A1, conformational epitope mapping

## Abstract

Annexin-A1 (ANXA1) belongs to a class of highly homologous Ca^2+^-dependent phospholipid-binding proteins. Its structure consists of a core region composed of four homologous repeats arranged in a compact, hydrolysis-resistant structure and an N-terminal region with a Ca^2+^-dependent conformation. ANXA1 is involved in several processes, including cell proliferation, apoptosis, metastasis, and the inflammatory response. Therefore, the development of antibodies blocking selected regions on ANXA1 holds great potential for the development of novel therapeutics treating inflammatory and cancer diseases. Here, we report the interaction site between an ANXA1-specific antibody known to inhibit T cell activation without adverse cytotoxic effects and ANXA1 using amide hydrogen–deuterium exchange mass spectrometry (HDX-MS). For the epitope determination, we applied two bottom-up HDX-MS approaches with pepsin digestion in solution and immobilized on beads. Both strategies revealed the interaction region within domain III of ANXA1 in Ca^2+^-bound conformation. The antibody-binding region correlates with the hydrophobic binding pocket of the N-terminal domain formed in the absence of calcium. This study demonstrates that even cryptic and flexible binding regions can be studied by HDX-MS, allowing a fast and efficient determination of the binding sites of antibodies which will help to define a mode of action profile for their use in therapy.

## 1. Introduction

Annexin A1 (ANXA1) is a 38-kDa protein which belongs to the annexin family of calcium-dependent phospholipid-binding proteins [1]. It contains a C-terminal core region, consisting of four homologous repeat domains (I–IV), of which each has five α-helices and a 41-amino-acid N-terminal region [2]. The C-terminal region is tightly compressed into a slightly curved disk, which renders the protein resistant to enzymatic hydrolysis. The convex face of the protein contains 12 calcium-binding sites [3] which, when occupied, lead to a conformational change that exposes the N-terminal domain III. Subsequently, two ANXA1 molecules can form a dimer or interact with a second bilayer [2]. The N-terminal domain is highly variable between the different members of the annexin family [4]. ANXA1 function is mediated through binding to the formyl peptide receptor (FPR) and/or the phospholipid bilayer of the cell membrane [5]. The role of ANXA1 has been investigated in a variety of different diseases, including cardiology, immunology, neurology, endocrinology, and oncology [5,6,7]. One of ANXA1’s most important properties is its ability to alter the innate and adaptive immune system [5,8]. In addition to the well-documented role in neutrophil and monocyte function in the innate immune system, it has been shown to modulate the signaling strength of the T cell receptor and consequently the T cell activation and differentiation [9,10]. Because of these properties, ANXA1 has been proposed as a therapeutic target for the treatment of T cell activation dysregulation diseases such as rheumatoid arthritis or multiple sclerosis [11,12].

In this study, we characterize the binding region of a therapeutic antibody candidate binding to ANXA1 in complex with calcium [11]. The humanized antibody was generated from a murine antibody that has been shown to specifically inhibit T cell activation without any adverse cytotoxic effects [12]. Knowing the interaction region between the antibody and ANXA1 will help to define a mode of action profile for its therapeutic use.

Epitopes of therapeutic monoclonal antibodies (mAbs) are often discontinuous and are only recognized by the mAb in their native conformational state [13]. A detailed mapping of such epitopes is challenging and there is only a limited number of technologies available. Of these technologies, hydrogen–deuterium exchange coupled to mass spectrometry (HDX-MS) does not introduce changes to the binding partners, such as the removal or addition of modifications (e.g., glycosylation, mutations, cross-linkers). In comparison to other structural high-resolution techniques, such as X-ray crystallography [14,15], NMR spectroscopy [16,17], or cryo-electron microscopy [18,19], HDX-MS is an effective method in terms of time, sample requirement, and throughput to supply coarse to highly resolved epitope structure information [13,20]. The method relies on differences in the solvent accessibility of epitope and non-epitope regions at the surface of an antigen. The exchange rate of backbone amide hydrogen atoms against deuterium slows down when the surface area is protected by the binding partner [21]. Following complex formation and deuteration, the antigen is proteolyzed by pepsin and the mass increase in the peptides is determined as a function of time to assess the level of deuteration [22]. Here, we report two epitope-mapping approaches based on HDX followed by pepsin digestion in solution and immobilized on beads, which both led to the elucidation of the ANXA1 surface region affected by antibody binding. Both approaches were adapted during the feasibility stage to the compact three-dimensional conformation of ANXA1 and to the calcium dependency of the antibody binding.

## 2. Results

### 2.1. Antigen and Antibody Characterization

**Figure 1:**
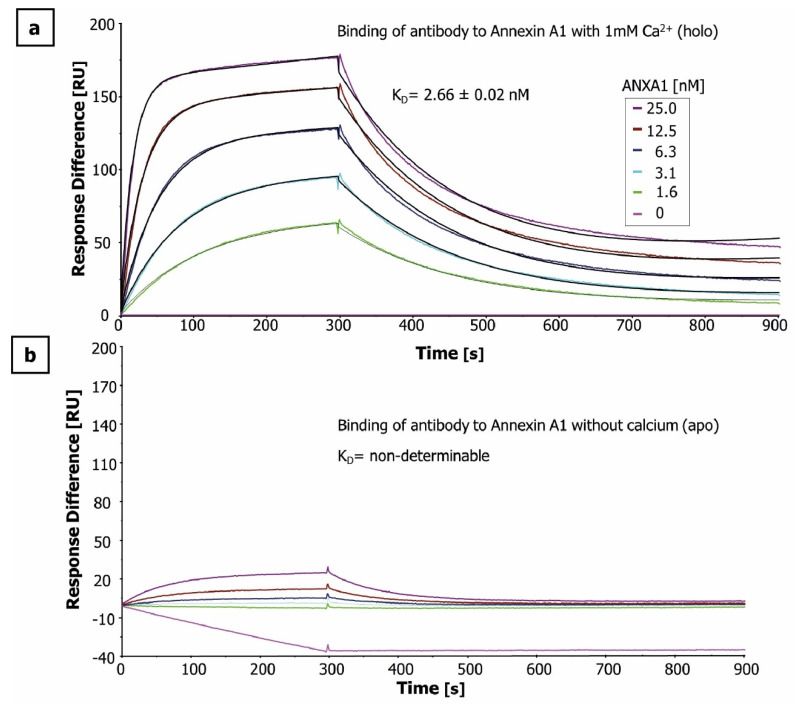
Binding affinities of anti-ANXA1 antibody. For surface plasmon resonance spectroscopy (SPR)-based affinity measurements, anti-ANXA1 antibody was immobilized via a covalently coupled anti-human IgG on a CM5 chip. Kinetic measurements were performed by injecting five concentrations of ANXA1 in presence and absence of calcium (holo (**a**), and apo (**b**), respectively) ranging from 1.6 to 25 nM. The obtained datasets were evaluated using the 1:1 Langmuir binding model. All measurements were performed in triplicate. Only one representative sensorgram is shown. The equilibrium dissociation constant in presence of calcium was determined to be 2.66 ± 0.02 nM, whereas the antibody did not bind to ANXA1 in the apo-state.

The antigen and the humanized murine anti-ANXA1 antibody (IgG1) were produced recombinant in *E. coli* and Chinese hamster ovary (CHO) cells, respectively. Their identity and purity were confirmed by HPLC-MS (Figure S1). Binding affinities of the anti-ANXA1-antibody for the two conformational states of ANXA1 (in the presence (holo) or absence (apo) of 1 mM Ca^2+^) were measured by surface plasmon resonance (SPR). For the Ca^2+^-bound holo-state of ANXA1, an equilibrium dissociation constant (K_D_) of 2.66 ± 0.02 nM was determined (Figure 1a), whereas no binding of the anti-ANXA1 antibody to the apo-state of ANXA1 was detectable (Figure 1b). This strongly suggests that the antibody exclusively binds to the Ca^2+^-bound holo-state of ANXA1. As a result, epitope characterization of the antibody–antigen complex formation was only performed in the presence of 1 mM Ca^2+^. To ensure a high degree of complex formation (>89%) and thereby avoid a false EX1 signature, the K_D_ value was used to calculate the antibody and antigen concentrations.

### 2.2. ANXA1 Deuteration Kinetics

**Figure 2:**
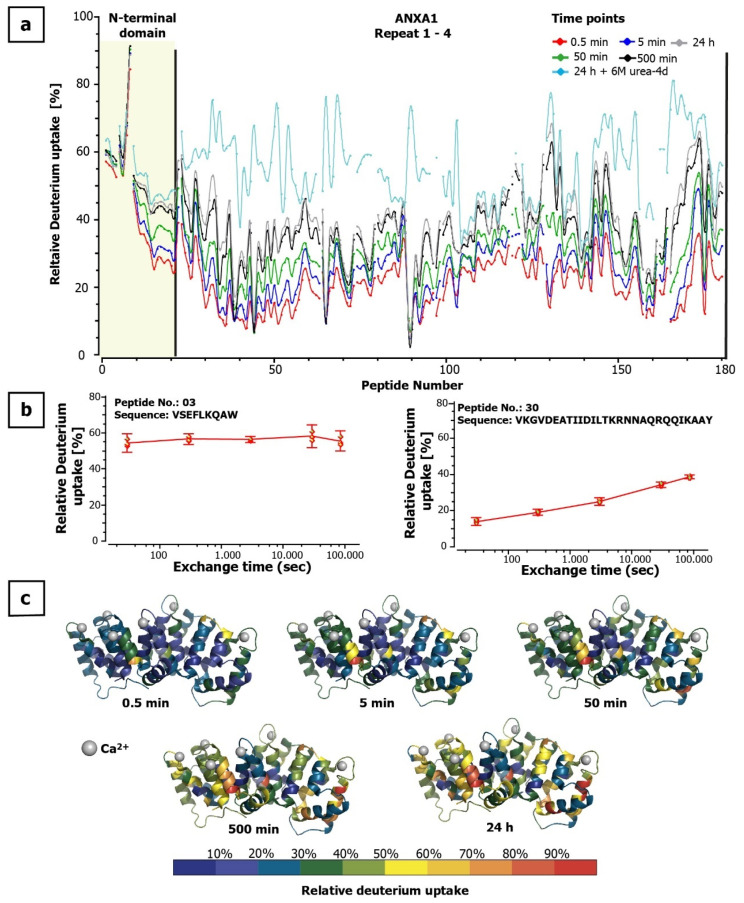
ANXA1 deuteration kinetics. (**a**) Relative deuterium uptake of 180 partially overlapping peptic peptides of ANXA1 numbered from N- to C-terminus over time. (**b**) Examples of deuterium uptake kinetics at peptide level: peptide #3: V5SEFLKQAW13 (left panel) showed very fast uptake whereas peptide #30: V58KGVDEATIIDILTKRNNAQRQQIKAAY85 (right panel) showed a slowly increasing uptake. (**c**) Heat map of deuterium uptake mapped onto the ribbon crystal structure of human ANXA1 taken from PDBe (1AIN [24]) using PyMOL (v2.0.7 http://www.pymol.org (accessed on 24 December 2020) and HDExaminer (v2.5.0, http://massspec.com/hdexaminer (accessed on 24 December 2020)) with medium smoothing for the partially overlapping peptic peptides. Dark blue regions showed a very slow exchange kinetics, indicating a compact, hardly accessible structure.

For the in-solution HDX approach, the amide proton to deuterium exchange in ANXA1 was carried out for 0.5, 5, 50, 500 min, and 24 h following the published recommendations for HDX-MS experiments [23]. For the immobilized pepsin approach, 5 and 500 min were chosen (Tables S1 and S2). The back exchange of the set-up was estimated to be 29% using synthetic peptides of different length (Table S3) and confirmed in full deuteration experiments (Figure 2 and Figure S2). Further HDX parameters are summarized in Table S2. Both digestion protocols showed 100% sequence coverage and similar HDX profiles. The results of the in-solution pepsin digest will be presented in detail. The unstructured part of the N-terminal domain covered by the first 10 peptic peptides showed a very fast uptake kinetics, with 60–80% deuteration after 0.5-min incubation with D_2_O. Thereafter, only a very low increase in deuteration was observed for this region. In contrast, peptic peptides covering highly structured domains, starting with the first α-helix of repeat I showing a slow deuteration rate. During the first 0.5 min, in which all the easily solvent-accessible amide hydrogens were exchanged, only an average relative deuterium uptake of 21% was observed. The deuteration kinetic of repeat III is faster compared to the other core domains. Even after 24 h of deuteration, no saturation was reached and some peptides still showed an ongoing uptake of deuterium (Figure 2). The average deuteration uptake as determined at peptide level was 41% after 24 h under native conditions. Full deuteration of ANXA1 was achieved in the presence of 6 M urea-d4 for 24 h at 20 °C. However, while protein repeats I, II, and IV showed a clear increase in deuteration following denaturation, the deuterium uptake of the N-terminus and domain III showed only a slight increase (Figure 2a and Figure S2).

### 2.3. HDX for Epitope Mapping of Anti-ANXA1 Antibody

**Figure 3:**
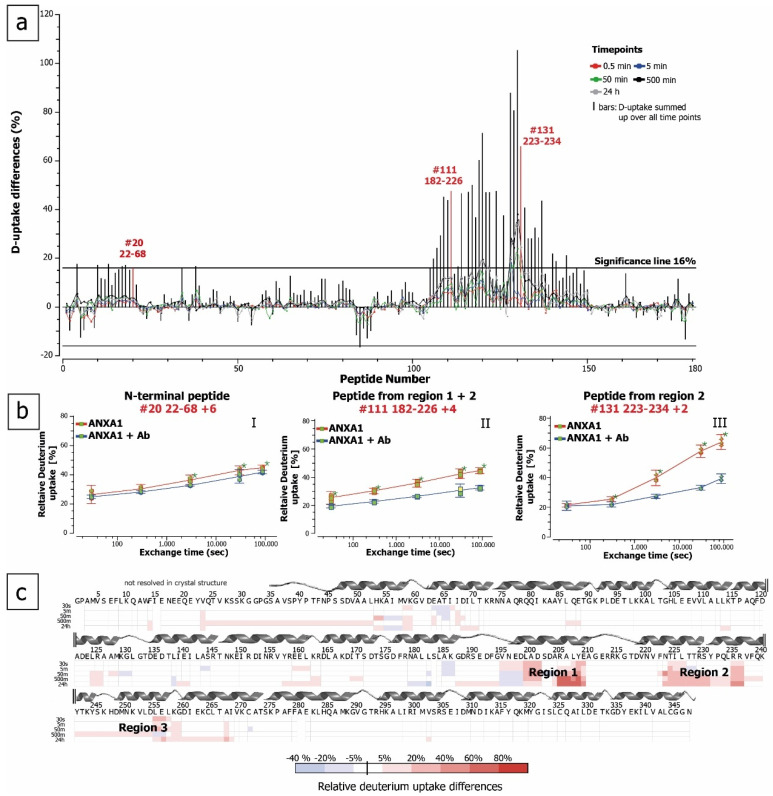
Epitope mapping of anti-ANXA1 by HDX mass spectrometry. (**a**) Differential deuterium uptake of ANXA1 alone and in complex with the anti-ANXA1 after different deuteration times and digestion with pepsin at 0 °C for 10 min. Partially overlapping peptic peptides are numbered from the N- to the C-terminus. High-confidence identification by mass, retention time, and charge was applied using a peptide library. (**b**) Examples of deuterium uptake plots for three peptides from the N-terminal region (I) and two epitope regions (II and III)) with and without antibody. * time point showing statistical differences with >95% confidence. (**c**) Heat map of anti-ANXA1 antibody epitope regions narrowed down after combining the data from all overlapping peptides and time points. The secondary structural behavior of the amino acids is illustrated based on the crystal structure of human ANXA1 (PDBe code: 1AIN [24]).

**Figure 4:**
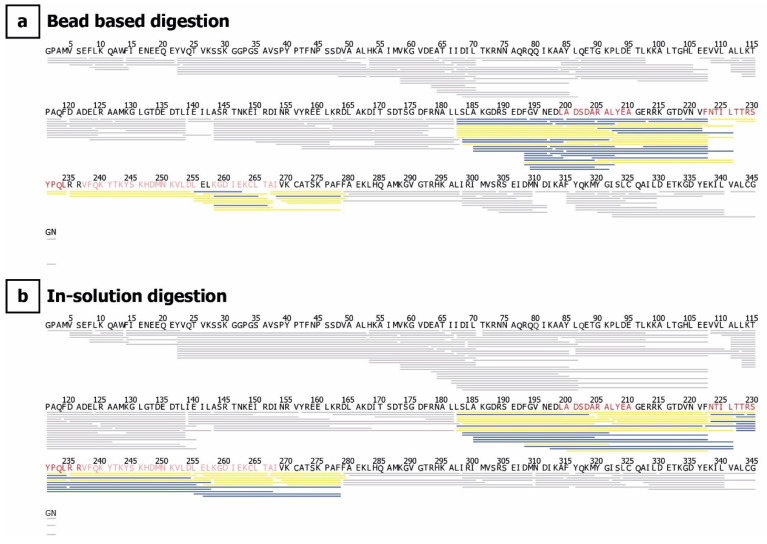
Sequence coverage (100%) and coverage redundancy of the bead-based pepsin digestion (**a**) and the in-solution pepsin digestion (**b**). Identical peptides within the antibody–ANXA1 interaction region are depicted in yellow; peptides obtained in one of the two approaches only are shown in blue. Amino acids highlighted in red show the refined interaction region, with significant deuteration differences in the covering peptides.

**Figure 5:**
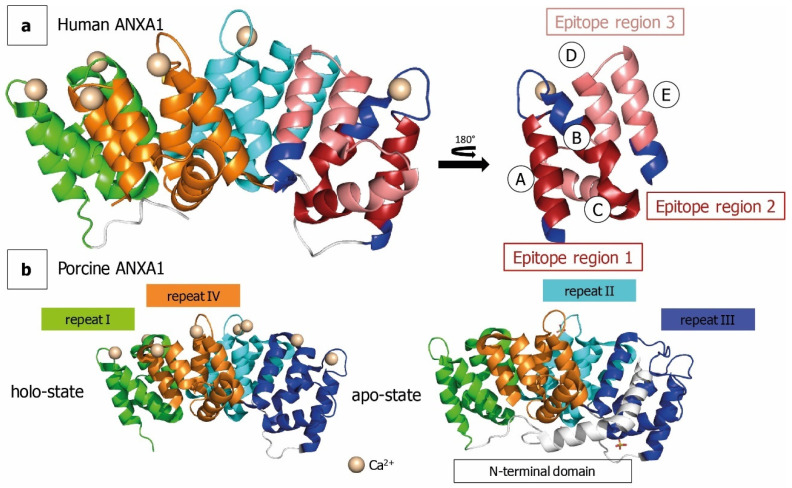
Crystal structures of ANXA1 with mapped epitope regions. (**a**) Ribbon diagram of human ANXA1 with calcium and mapped epitope regions (PDB code: 1AIN [24]). α-helices were named from A to E. Epitope regions showing >40% differences in deuterium uptake for bound and unbound ANXA1 are shown in dark red. Regions with 20–40% difference in deuterium uptake between the bound and unbound antibody state are drawn in light red. (**b**) Ribbon diagram of porcine ANXA1 in absence of calcium (right panel, PDB-code: 1HM6 [2]) and presence of calcium (left panel, PDB code: 1MCX [25]). Figure was generated using PyMOL (v2.0.7 http://www.pymol.org (accessed on 24 December 2020)).

We found significant differences in deuterium uptake between ANXA1 alone and when complexed with anti-ANXA1 antibody in peptides covering the N-terminus and domain III (Figure 3a). The bars represent the sum of all differences across the measured time points. Using the variance, a ΔHX threshold of 0.64 Da was calculated. Peptides crossing the significance line (five times ΔHX; 5 time points) were considered to show significant differences in deuterium uptake (Figure 3a). Strong protection was found in two regions within the core of ANXA1. This protective effect of the antibody is shown by two example peptides consisting of residues 223–234 (>2-Da mass shift after 24 h deuteration, corresponding to 25% of exchangeable amide protons, Figure 3b, right panel) and residues 182–226 (>5-Da mass shift, corresponding to 13% of exchangeable amide protons, Figure 3b, middle panel). Taking into consideration all amino acids within peptides showing significant differences in HDX, the ANXA1 region affected by antibody binding is located within amino acids 182 to 278. Using the software HDExaminer, a further refinement of the binding region based on HDX data from partially overlapping peptides was performed. The results presented in Figure 3c highlight individual amino acids according to differences found in the HDX profiles of all peptides containing the respective amino acid. The refinement results were performed for both digestion protocols separately. Taking into account that the peptide sets obtained from the two digestion approaches differ in the sequence stretch covered, peptide length, and time of exposure to H_2_O, it is notable that refinement results were similar (Figure 3c and Figure 4 and Figure S3) even though the back exchange was lower and the repeatability (ΔHX threshold) was better using the bead-based digestion (Table S2). The refined interaction sites were located more precisely to residues 199–210 (region I) and 223–236 (region II) (Figure 3c). Although the regions I and II are 14 amino acids apart in the primary sequence, they are proximal in the three-dimensional structure and constitute one spatial region on the surface of the antigen. These regions form two α-helices in repeat III of ANXA1 (Helix A and B, Figure 5). Additionally, peptides covering amino acids 237–268 were found to show modest but statistically significant deuteration protection in the presence of the antibody. This region is located in three spatially close α-helices (Helix C, D, and E, Figure 5a) and some amino acids within this region may contribute to the binding. This slight protection in deuterium exchange is better preserved using the bead-based digest (Figure S7). A fourth region in proximity to the N-terminus also showed slight differences (<4% per time point) in deuterium uptake (Figure 3a). However, when examining data from all peptides covering this region, only one exchange time point showed a significant but very low difference in deuterium uptake (Figure 3c). This difference might therefore be due to changes in the dynamics or conformation upon antibody binding as opposed to direct interaction. Interestingly, all interacting regions undergo major structural changes upon calcium binding, as illustrated by the two crystal structures obtained from porcine ANXA1 in the presence and absence of calcium (Figure 5b, PDBe-code: 1MCX [25] and 1HM6 [2]). A manual examination of the isotopic distribution of protected region-containing peptides supports their belonging to the latter. The deuterated isotope profile of the peptides obtained from ANXA1 alone resembles closely a Gaussian distribution (Figure S4, top panel) featuring local unfolding by a “rapid breathing” of the local environment. Upon antibody binding, the local unfolding is reduced and the isotopic exchange profile becomes asymmetrical (Figure S4, middle panel), supporting earlier findings that the antibody binding not only excludes solvent from epitope regions but also stabilizes the local structure [26].

## 3. Discussion

HDX-MS is a well-established technology to study the structural dynamics of proteins or elucidate protein–protein interaction sites. However, the bottom-up approach relies on good sequence coverage and redundancy. Inefficient pepsin cleavage and poor solvent accessibility of amide protons in rigid proteins remain a challenge for HDX. In the literature, immobilized pepsin is often used to improve digestion efficiency and shorten the digestion times [23,27,28].

Here, we compared two strategies, the in-solution and immobilized pepsin digestion, to reveal the epitopes of an ANXA1-specific antibody which is intended for clinical applications. The usage of immobilized pepsin resulted in higher recovery of peptic peptides due to higher signal intensity and consequently lower D-uptake deviation within the triplicate measurements. To our knowledge, this is the first report of HDX-MS analysis of ANXA1 and our results confirm the high solvent accessibility of the N-terminal domain upon Ca^2+^ binding. The amino acid residues belonging to the N-terminus were not resolved in the holo-state and are therefore missing in the published X-ray crystal structure (Figure 5) [2]. The deuteration kinetic at the peptide level followed a regular pattern of high and low deuteration (Figure 3). Notably, this finding correlates to the pattern of the solvent accessibility surface area (SASA) calculated from the X-ray structure of porcine ANXA1 across the amino acid sequence (Figure S5). Both patterns can be explained by the three-dimensional structure of ANXA1, which is composed of 20 α-helices whose amino acids show higher and lower solvent accessibility in an alternate manner. Furthermore, this explains the relatively high initial deuteration rate (23% D uptake) within the first 30 s, followed by a very slow increase over time. The special three-dimensional structure of ANXA1 precludes a prediction or confirmation of potential epitope regions by SASA analysis, as previously suggested [29]. Peptides covering the N-terminal region and repeat III did not show a further increase in deuterium uptake in the fully deuterated sample compared to the longest deuteration time point under native conditions (24 h). This suggests that domain III, which also showed high deuteration protection upon binding to the antibody, is more accessible to the solvent (and antibody) than expected due to the depicted crystal structure.

The deuteration profile and the regions showing exchange protection upon antibody binding, as well as the HDExaminer-refined regions, are similar whether using immobilized or solubilized pepsin digestion (Figure 3 vs. Figure S3). Two regions in repeat III show >40% differences in D-uptake upon antibody binding. They consist of α-helices A and B/C, which undergo extensive structural changes upon calcium binding. Only in the apo-state without calcium ions do Phe^221^; Leu^225^, Phe^237^, and Val^268^ form a hydrophobic pocket harboring the N-terminal domain [25]. These amino acids are exactly within the protected region of the antibody. In the holo-state, the N-terminus is released and interactions with the lipid bilayer or dimer formation are triggered [3]. A third region with deuterium uptake differences between 20 and 40% contains two amino acid patches belonging to the α-helices D and E. Helix D is present in holo-ANXA1 only and becomes unstructured without calcium. This conformational change might prevent the antibody from binding to apo-ANXA1. This binding specificity in targeting only the biological active form of ANXA1 makes the humanized antibody an attractive therapeutic candidate. It may be used in the treatment of T-cell-mediated diseases to reduce the extracellular concentration of the holo-ANXA1 form. Low levels of holo-ANXA1 have been found to display impaired activation and increased differentiation of CD4+ T-cells into Th2 cells [9]. While the ability to reduce the T cell activity has been reported for the original murine antibody [12], further mode-of-action studies are required to clarify the impact of the antibody–antigen complex formation on the biological functions of ANXA1.

## 4. Material and Methods

### 4.1. Reagents, Peptides, and Antibody

First, 4-(2-hydroxyethyl)-1-piperazineethanesulfonic free acid (HEPES) was obtained from VWR Life Science (Darmstadt, Germany). Formic acid was obtained from LGC Standards (Teddington, United Kingdom). Tris(2-carboxyethyl)phosphine (TCEP), calcium chloride, sodium chloride, and guanidinium hydrochloride were obtained from Carl Roth (Karlsruhe, Germany). Ammonium formate was obtained from Fluka (Munich, Germany). Pepsin from porcine gastric mucosa (3200–4500 U/mg), ethylenediaminetetraacetic acid tetra sodium salt dihydrate (EDTA), urea-d4 (98 atom %D), and 99.9% deuterium oxide were obtained from Sigma-Aldrich (Munich, Germany). Pierce immobilized porcine pepsin was obtained from Thermo Scientific (Schwerte, Germany). PNGase F was obtained from R&D Systems GmbH (Wiesbaden, Germany). The human monoclonal anti-ANXA1-antibody (IgG1) was obtained by humanization of a murine IgG2b antibody that was obtained from mice by genetic immunization with human ANXA1, as previously described [12]. The murine hybridoma cell line is deposited within the European Collection of Cell Cultures (accession number 10060301). The humanized antibody was further sequence-optimized to remove potential sequence instabilities in CDR regions [11] and was produced in a CHO-based transient expression system. Formulation buffer was phosphate buffer saline (PBS), pH 7.4. For epitope mapping, a stock solution of the antibody was prepared by buffer exchange into 20 mM HEPES buffer containing 150 mM NaCl and 2 mM CaCl_2_ at a stock solution of 5.4 µg/µL (36.6 µM). All synthetic peptides used for determination of the HDX back exchange were obtained from Intavis (Tübingen, Germany).

### 4.2. ANXA1 Production and Purification

ANXA1 cDNA was cloned into a pGEX-6P-1 expression vector. The protein was expressed as GST-ANXA1 fusion protein in *E. coli* BL21(DE3) cells obtained from New England Biolabs (Ipswich, England). Purification of the GST-ANXA1 from soluble *E. coli* extract was achieved by affinity chromatography using GSTrap FF (GE Health Care, Freiburg, Germany) according to the manufacturer’s instructions. After buffer exchange into PreScission protease cleavage buffer using a HiTrap desalting column (GE Healthcare, Freiburg, Germany), the GST tag was cleaved off upon incubation with PreScission protease (GE Healthcare). Subsequently, GST was removed by affinity capture using Glutathione Sepharose 4B (GE Healthcare, Freiburg, Germany). Residual amounts of GST were removed by incubation with GST Trap resin (ChromoTek, Martinsried, Germany). Finally, the buffer comprising cleaved ANXA1 was exchanged to 20 mM HEPES pH 6.0, 150 mM NaCl by dialysis using D-Tube Dialyzer (Merck Millipore, Massachusetts, US). Purified ANXA1 was stored at a concentration of 1.6 mg/mL at −78 °C. Purity and integrity of produced ANXA1 were examined by SDS PAGE followed by Coomassie staining and mass spectrometry.

### 4.3. HPLC-MS Analysis of ANXA1 and Antibody

First, 1.6 µg (4 µL) of ANXA1 and PNGaseF-deglycosylated antibody, respectively, was analyzed by LC-MS using a UHPLC system (UltiMate 3000, RSLCnano, Dionex GmbH, Idstein, Germany) coupled to a QTOF-type mass spectrometer (MaXis HD UHR q-TOF, Bruker, Bremen, Germany). Reversed-phase chromatography (ACQUITY BEH C4, 1.7 µm, 300 Å, 1 mm × 50 mm, Waters GmbH, Eschborn, Germany) was applied for desalting. The solvents were 0.1% formic acid in (A) water (Carl Roth, Karlsruhe, Germany) and in (B) acetonitrile (Carl Roth, Karlsruhe, Germany), respectively. A stepwise linear gradient was run at 75 °C at a flow rate of 150 µL/min (0–0.4 min, 5% B; 2.5 min 30% B; 7 min, 50%B) followed by wash steps. Mass spectrometer parameters were adapted to the size of the molecule and the LC flow rate.

### 4.4. Determination of Affinity by Surface Plasmon Resonance (SPR)

SPR interaction analysis was performed on a Biacore 3000 system (GE Healthcare, Freiburg, Germany). For ANXA1 interaction analysis, purified goat antihuman IgG Fc (Jackson ImmunoResearch Laboratories, Cambridgeshire, UK) was immobilized to achieve a level of 13,000 response units (RU). Immobilization of the capturing antibody was performed on a CM5 chip (GE Healthcare, Freiburg, Germany) using amino coupling chemistry. Anti-ANXA1 IgG was captured by the anti-human IgG at a concentration of 7 nM with a pulse of 60 s at a flow rate of 10 µL/min to achieve a binding level of 300 (±50) RU on the measuring flow cells. Binding experiments were carried out in HEPES-buffered saline (10 mM HEPES, 150 mM NaCl, 3.4 mM EDTA or 1 mM CaCl2, and 0.05% (*v*/*v*) P20 surfactant) at 25 °C. Kinetic data were collected at five different concentrations of ANXA1 with or without calcium, 25, 12.5, 6.25, 3.125, and 1.563 nM, through all flow cells to record the association phase. The analyte was allowed to associate for 5 min, followed by a dissociation phase in buffer for 10 min at 30 µL/min flow rate. At the end of each binding cycle, the sensor surface was regenerated by 8-min injections of 10 mM glycine HCl, pH 2.0, at a flow rate of 10 µL/min, followed by 5-s injections of 50 mM NaOH at a flow rate of 60 µL/min. The data were fitted to a 1:1 binding with drifting baseline using the BIAevaluation 4.1 software (GE Healthcare, Freiburg, Germany). Kinetic rate constants were determined from the fits of the association and dissociation phases, and the K_D_ was derived from the ratio of these constants.

### 4.5. Hydrogen–Deuterium Exchange

#### ANXA1 Deuteration Kinetics and Epitope Elucidation

ANXA1 (5 µL) was incubated with and without anti-ANXA1 (5 µL) at 20 °C for 10 min. Deuterium exchange of the pre-incubated antibody–antigen complex was initiated by dilution with 90 µL HEPES-buffered saline (10 mM HEPES, 150 mM NaCl, pH 7.4, with 1 mM CaCl_2_) prepared with D_2_O. The final concentrations in the exchange reaction were 2.0 and 1.8 µM for ANXA1 and antibody, respectively. To ensure a minimum of 89% of complex formation, the molar ratio of antigen to antibody was calculated as per [30], using the equilibrium dissociation constant (K_D_) of 2.66 nM determined by SPR analysis. The final D_2_O concentration was 90%. After 0.5, 5, 50, 500 min, and 24 h at 20 °C, aliquots of 20 µL were taken and quenched by adding 20 µL ice-cold quenching solution (0.4 M TCEP and 0.8 M guanidine HCl in 100 mM ammonium formate solution pH 2.2), resulting in a final pH of 2.5. The HDX of the bead-based digest was followed for 5 and 500 min. The immobilized pepsin was prepared by adding 40 µL of 50% slurry (in ammonium formate solution pH 2.5) to a tube and dried by centrifugation at 1000× *g* for 3 min at 0 °C and discarding the supernatant. Quenched samples were immediately snap-frozen. Before injection, aliquots were thawed and either 0.45 µL pepsin (100 µM) was added to the quenched samples (in-solution digest) or the samples were added to the dried pepsin beads (bead-based digest). Proteolysis of the in-solution and bead-based digest was performed for 10 min or 1 min, respectively, in a water ice bath. The samples of the in-solution digest were injected immediately into an LC-MS system. The samples prepared with the bead-based digest were added to a 22-µm filterand the beads were separated by centrifugation at 1000× *g* for 30 s at 0 °C and were afterwards also applied to an LC-MS system. Undeuterated control samples were prepared under the same conditions using HEPES-buffered saline prepared with H_2_O instead of D_2_O. All experiments were performed in triplicate. A high-deuterated control was prepared for 24 h at 20 °C. Labeling was performed using HEPES-buffered saline (50 mM HEPES, 150 mM NaCl, pH 7.4, with 1 mM CaCl_2_) in D_2_O with addition of 6 M urea-d4 to the deuteration buffer. Samples were prepared and analyzed as described above. Samples were quenched by adding 1:1 (*v*/*v*) ice-cold quenching solution (0.2 M TCEP in 100 mM ammonium formate solution pH 2.6) to pH 3.0. Digest and analysis were performed as described above.

The overall deuterium recovery of the method was determined using 14 synthetic peptides with different lengths and compositions (see supplement). A stock solution was prepared containing 1 mg/mL of each peptide. Aliquots of 1 µL of the stock solution were diluted 1:10 in PBS and freeze-dried. Deuteration was initiated by dissolving each aliquot in 10 µL, 99.9% D_2_O. After overnight (ON) incubation at 20 °C, deuteration was quenched by adding 10 µL of quenching solution to each aliquot. The proteolysis step was simulated by storing the samples on ice or at 20 °C for 2, 5, and 10 min. The samples were injected into the LC-MS system immediately after incubation.

### 4.6. Chromatography, Mass Spectrometry, and HDX Data Analysis

HDX data acquisition and analysis was performed as previously described [31]. For data analysis, an ANXA1 peptide list was generated from an LC-MS/MS peptide map using database search and applying the pepsin specificity rules described by Hamuro et al. [32]. For the database search, Mascot (v2.3.02, Matrix Science LTD, London, UK) was used together with a protein database containing the amino acid sequences of ANXA1, the antibody, and pepsin. ANXA1 peptides overlapping with antibody peptides were removed according to criteria reported in [31]. No back exchange correction was applied. The significance level for deuterium uptake differences between bound and unbound state was set to 0.05 and calculated using Student’s t-test. The significance level of 0.64 Da displayed in the residual plot was calculated using the variance of the deuterium uptake of all peptide triplicates followed for HDX. Peptides used to calculate the heat map were considered as significant if the summed difference between both states was higher than five times the significance level displayed by the significance line (number of time points multiplied by the significance level).

## Data Availability

Not applicable.

## Supplementary Materials

The following are available online at https://www.mdpi.com/2073-4468/10/1/11/s1, Supplementary information accompanies this paper at Supplements.pdf.

## References

[1] Rosengarth A., Luecke H. (2000). Crystallization and preliminary X-ray analysis of full-length annexin i comprising the core and n-terminal domain. Acta Crystallogr. D Biol. Crystallogr..

[2] Rosengarth A., Gerke V., Luecke H. (2001). X-ray structure of full-length annexin 1 and implications for membrane aggregation. J. Mol. Biol..

[3] Lizarbe M.A., Barrasa J.I., Olmo N., Gavilanes F., Turnay J. (2013). Annexin-phospholipid interactions. Functional implications. Int. J. Mol. Sci..

[4] Liemann S., Huber R. (1997). Three-dimensional structure of annexins. Cell. Mol. Life Sci..

[5] Sheikh H.M., Solito E. (2018). Annexin a1: Uncovering the many talents of an old protein. Int. J. Mol. Sci..

[6] Purvis G.S.D., Solito E., Thiemermann C. (2019). Annexin-a1: Therapeutic potential in microvascular disease. Front. Immunol..

[7] Shao G., Zhou H., Zhang Q., Jin Y., Fu C. (2019). Advancements of annexin a1 in inflammation and tumorigenesis. OncoTargets Ther..

[8] D’Acquisto F., Perretti M., Flower R.J. (2008). Annexin-a1: A pivotal regulator of the innate and adaptive immune systems. Br. J. Pharmacol..

[9] D’Acquisto F., Paschalidis N., Sampaio A.L., Merghani A., Flower R.J., Perretti M. (2007). Impaired t cell activation and increased th2 lineage commitment in annexin-1-deficient t cells. Eur. J. Immunol..

[10] D’Acquisto F., Merghani A., Lecona E., Rosignoli G., Raza K., Buckley C.D., Flower R.J., Perretti M. (2007). Annexin-1 modulates t-cell activation and differentiation. Blood.

[11] Hays H.C.W., Wood C.B., Flatau T.C. (2020). Anti Human Annexin a1 Antibody. Patent.

[12] D’Acquisto F., Perretti M. (2011). Annexin 1 Antibody. Patent.

[13] Abbott W.M., Damschroder M.M., Lowe D.C. (2014). Current approaches to fine mapping of antigen-antibody interactions. Immunology.

[14] Zoll S., Schlag M., Shkumatov A.V., Rautenberg M., Svergun D.I., Gotz F., Stehle T. (2012). Ligand-binding properties and conformational dynamics of autolysin repeat domains in staphylococcal cell wall recognition. J. Bacteriol..

[15] Davies D.R., Cohen G.H. (1996). Interactions of protein antigens with antibodies. Proc. Natl. Acad. Sci. USA.

[16] Monaco S., Tailford L.E., Juge N., Angulo J. (2017). Differential epitope mapping by std nmr spectroscopy to reveal the nature of protein-ligand contacts. Angew. Chem. Int. Ed..

[17] Becker W., Bhattiprolu K.C., Gubensak N., Zangger K. (2018). Investigating protein-ligand interactions by solution nuclear magnetic resonance spectroscopy. Chemphyschem.

[18] Bianchi M., Turner H.L., Nogal B., Cottrell C.A., Oyen D., Pauthner M., Bastidas R., Nedellec R., McCoy L.E., Wilson I.A. (2018). Electron-microscopy-based epitope mapping defines specificities of polyclonal antibodies elicited during hiv-1 bg505 envelope trimer immunization. Immunity.

[19] Renaud J.P., Chari A., Ciferri C., Liu W.T., Remigy H.W., Stark H., Wiesmann C. (2018). Cryo-em in drug discovery: Achievements, limitations and prospects. Nat. Rev. Drug Discov..

[20] Hansen J., Baum A., Pascal K.E., Russo V., Giordano S., Wloga E., Fulton B.O., Yan Y., Koon K., Patel K. (2020). Studies in humanized mice and convalescent humans yield a sars-cov-2 antibody cocktail. Science.

[21] Pandit D., Tuske S.J., Coales S.J., Yen E S., Liu A., Lee J.E., Morrow J.A., Nemeth J.F., Hamuro Y. (2012). Mapping of discontinuous conformational epitopes by amide hydrogen/deuterium exchange mass spectrometry and computational docking. J. Mol. Recognit..

[22] Englander S.W. (2006). Hydrogen exchange and mass spectrometry: A historical perspective. J. Am. Soc. Mass Spectrom..

[23] Masson G.R., Burke J.E., Ahn N.G., Anand G.S., Borchers C., Brier S., Bou-Assaf G.M., Engen J.R., Englander S.W., Faber J. (2019). Recommendations for performing, interpreting and reporting hydrogen deuterium exchange mass spectrometry (hdx-ms) experiments. Nat. Methods.

[24] Weng X., Luecke H., Song I.S., Kang D.S., Kim S.H., Huber R. (1993). Crystal structure of human annexin i at 2.5 a resolution. Protein Sci..

[25] Rosengarth A., Luecke H. (2003). A calcium-driven conformational switch of the n-terminal and core domains of annexin a1. J. Mol. Biol..

[26] Hao G., Wesolowski J.S., Jiang X., Lauder S., Sood V.D. (2015). Epitope characterization of an anti-pd-l1 antibody using orthogonal approaches. J. Mol. Recognit..

[27] Puchades C., Kukrer B., Diefenbach O., Sneekes-Vriese E., Juraszek J., Koudstaal W., Apetri A. (2019). Epitope mapping of diverse influenza hemagglutinin drug candidates using hdx-ms. Sci. Rep..

[28] Kielkopf C.S., Ghosh M., Anand G.S., Brown S.H.J. (2019). Hdx-ms reveals orthosteric and allosteric changes in apolipoprotein-d structural dynamics upon binding of progesterone. Protein Sci..

[29] Huang R.Y.C., Krystek S.R., Felix N., Graziano R.F., Srinivasan M., Pashine A., Chen G. (2017). Hydrogen/deuterium exchange mass spectrometry and computational modeling reveal a discontinuous epitope of an antibody/tl1a interaction. mAbs.

[30] Kochert B.A., Iacob R.E., Wales T.E., Makriyannis A., Engen J.R. (2018). Hydrogen-deuterium exchange mass spectrometry to study protein complexes. Methods Mol. Biol..

[31] Wagner T.R., Kaiser P.D., Gramlich M., Ostertag E., Ruetalo N., Junker D., Haering J., Traenkle B., Becker M., Dulovic A. (2020). Neutrobodyplex—nanobodies to monitor a sars-cov-2 neutralizing immune response. bioRxiv.

[32] Hamuro Y., Coales S.J. (2018). Optimization of feasibility stage for hydrogen/deuterium exchange mass spectrometry. J. Am. Soc. Mass Spectrom..

