# Supplementary material for: HDX-MS for Epitope Characterization of a Therapeutic ANTIBODY Candidate on the Calcium-Binding Protein Annexin-A1
Source: Antibodies (Basel). 2021 Mar 19;10(1):11. doi: 10.3390/antib10010011 (PMC8006148; doi:10.3390/antib10010011)
Supplement: Supplementary file 1 [file antibodies-10-00011-s001.pdf]

Supplemental information

# HDX-MS for epitope characterization of a therapeutic antibody candidate on the calcium-binding protein annexin-A1

**Marius Gramlich[1], Henry C. W. Hays[2], Scott Crichton[2], Philipp D. Kaiser[1], Anne Heine[1], Nicole Schneiderhan-Marra[1], Ulrich Rothbauer[1,3], Dieter Stoll[1,4], Sandra Maier[1], Anne Zeck[1]**

[1]NMI, Natural and Medical Sciences Institute at the University of Tuebingen, Markwiesenstr. 55, 72770 Reutlingen, Germany; [2]MedAnnex Ltd,1 Lochrin Square, Fountainbridge, Edinburgh EH3 9QA, Scotland; [3]Pharmaceutical Biotechnology, Eberhard Karls University Tuebingen, Geschwister-Scholl-Platz, 72074 Tuebingen; [4]University of Applied Sciences Albstadt-Sigmaringen, Anton-Guentherstr. 51, 72488 Sigmaringen, Germany;

Table S1: Comparison of different parameters between the in-solution and the bead based digest.

| **Characteristic parameters** | In solution digest | Bead based digest |
|---|---|---|
| Identified ANXA1 peptides | 220 | 228 |
| Sequence coverage ANXA1 | 100 % | 100 % |
| Ab Sequence coverage LC/HC | 100% / 57% | 100% / 62% |
| Identified LC/HC peptides | 65 / 70 | 84 / 88 |
| Interfering Ab and ANXA1 peptides | 2 | 3 |
| Sequence coverage pepsin | 44% | 74% |
| Identified pepsin peptides | 26 | 79 |
| Interfering pepsin and ANXA1 peptides | - | - |
| Peptides followed for HDX | 180 (81.8%) | 207 (90.8%) |

Table S2: HDX-MS Summary

| **Table HDX summary** | In solution digest | Bead based digest |
|---|---|---|
| State | ANXA1 & ANXA1 bound by Ab | ANXA1 & ANXA1 bound by Ab |
| HDX reaction details | 10 mM HEPES pH 7.4, 1 mM $CaCl_2$ , 20 °C, 90% $D_2O$ | 10 mM HEPES pH 7.4, 1 mM $CaCl_2$ , 20 °C, 90% $D_2O$ |
| Time points | 30s, 300s, 3000s, 30000s, 86400s, | 300s, 3000s |
| Av. back exchange (Synthetic peptides) | 29% | 24 % |
| Digest conditions | 10 min on-Ice, 1:1 pepsin:ANXA1 molar ratio | 1 min on-Ice, 20 µl pepsin beads |
| Number of Peptides /Sequence coverage | 180 / 100% | 207 / 100 % |
| Average peptide length / average redundancy | 21 (Std. Dev. 12) / 11 | 15 (Std. Dev. 9) / 9.1 |
| Technical replicates (triplicate) | min 2 of 3 peptides per time point, in both states | min 2 of 3 peptides per time point, in both states |
| Determined ΔHX threshold for each time point | 0.64 Da | 0.25 Da |
| Significant differences in HDX | Students t-distribution on 95% confidence level | Students t-distribution on 95% confideñnce level |

Table S3: List of the used systemic peptides for back exchange determination. * marks a carbamidomethylated cysteine.

| #No | Sequence | Length | Retention time [min] |
|---|---|---|---|
| 1 | LTIEELK | 7 | 16.3 |
| 2 | FNNYQVR | 7 | 7.9 |
| 3 | MSDSVILR | 8 | 14.5 |
| 4 | SEC*HVDFFR | 9 | 17.3 |
| 5 | TVAAFGGEK | 9 | 5.1 |
| 6 | IVVLC*GQEAVK | 11 | 14.8 |
| 7 | LQDEIDAALPNK | 12 | 15.8 |
| 8 | GTTLITNLSSVLK | 13 | 20.4 |
| 9 | AAATEDATPAALEK | 14 | 11.4 |
| 10 | ISIIPQDPILFPGSLR | 16 | 21 |
| 11 | EQLDSLVC*LESAILEVLR | 18 | 23.4 |
| 12 | LSDRPQLPYLEAFILETFR | 19 | 22.7 |
| 13 | AMDSFPGPPTHWLFGHALEIQK | 22 | 20.6 |
| 14 | GDFVAVFPPMIHNDPEVFDAPK | 22 | 20.5 |
| 15 | VYGPVFTLYFGSKPTVVVHGYEAVK | 25 | 20.7 |

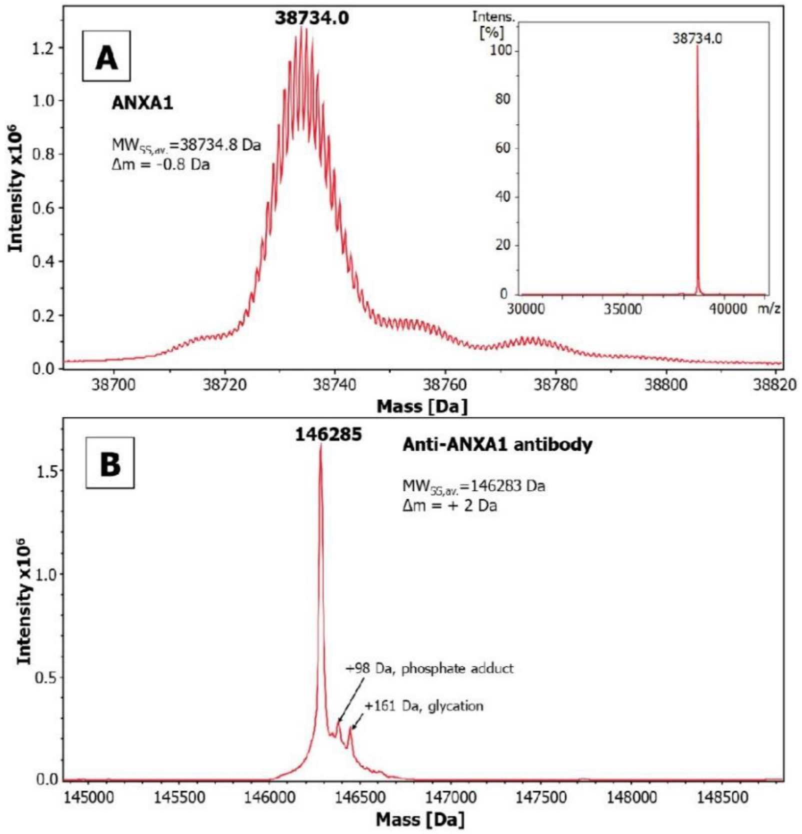


Figure S1: High resolution deconvoluted ESI QTOF MS spectrum of recombinant ANXA1 (A) and deglycosylated anti-ANXA1 antibody (B) in positive mode. The experimental mass of the molecules confirmed the theoretical mass and identity of both molecules. Integrity was good with no degradation or contamination as displayed in the inset of (A). The antibody showed 14% glycation (B).

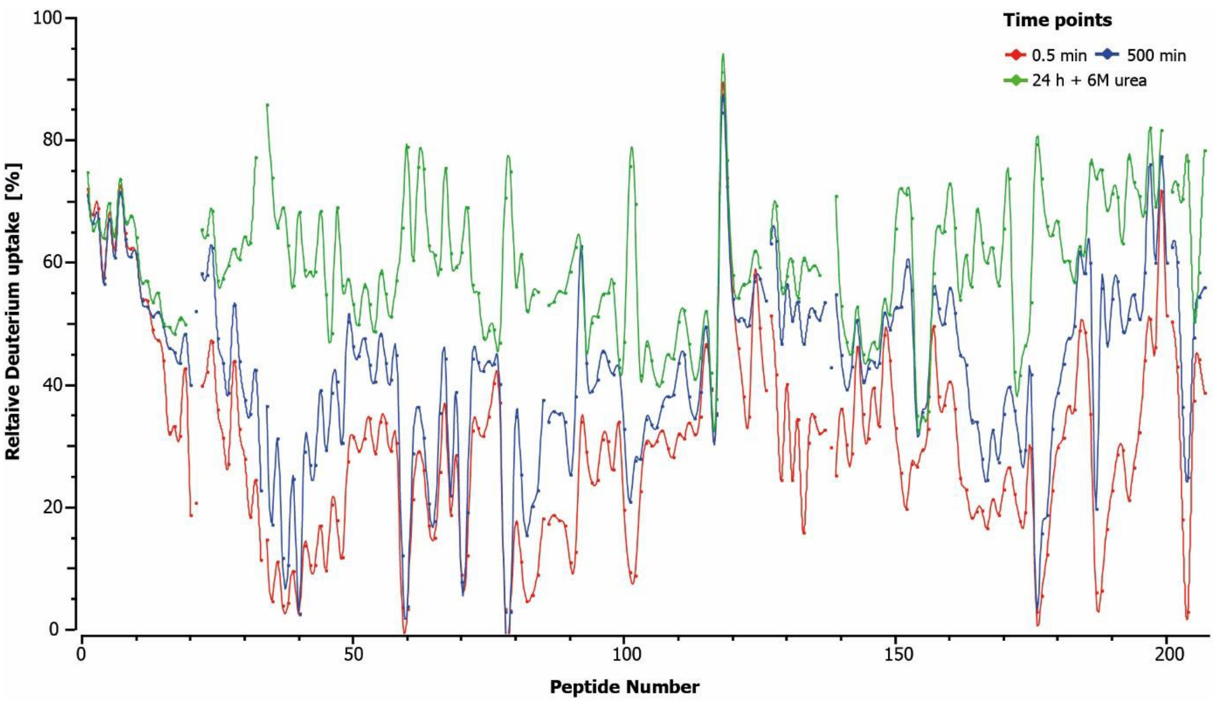


Figure S2: ANXA1 deuteration kinetics of the bead-based digest. Relative deuterium uptake of peptic peptides of ANXA1 numbered from N- to C-terminus over time.

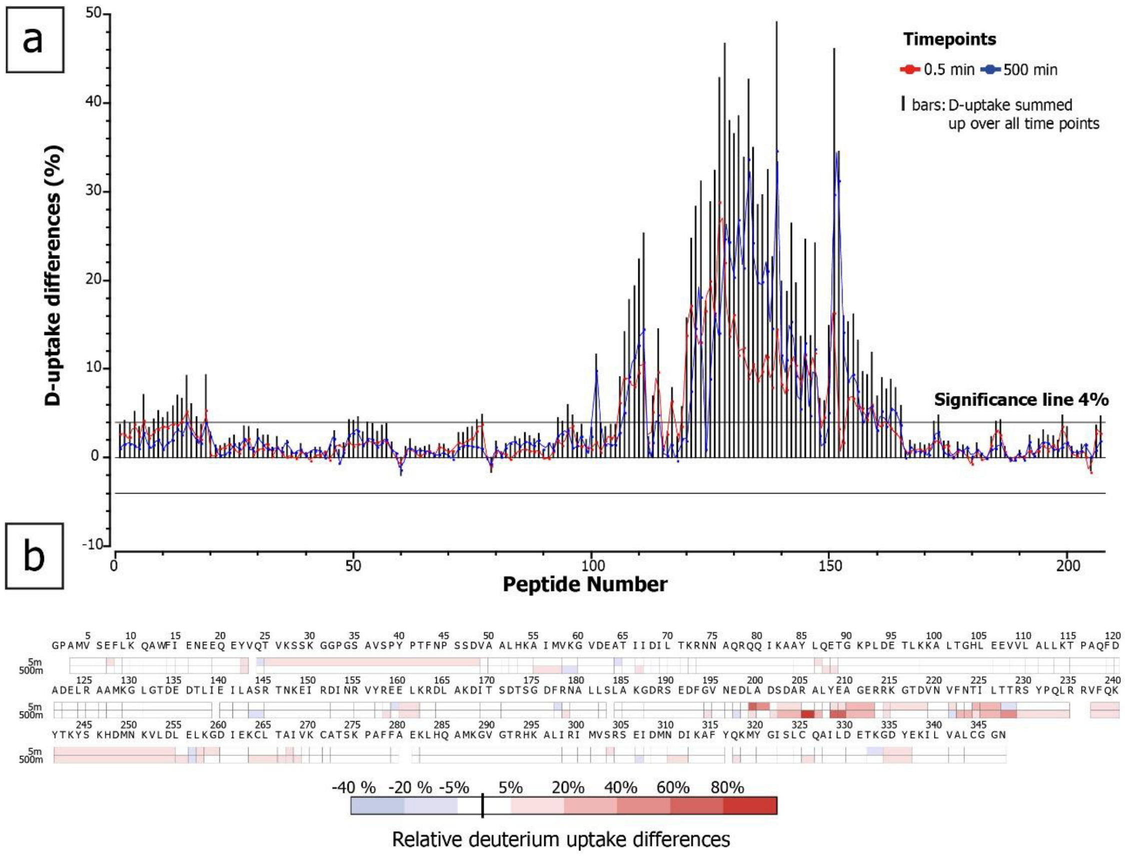


Figure S3: Epitope mapping of anti-ANXA1 by HDX mass spectrometry of the bead-based digest. (a) Differential deuterium uptake of ANXA1 alone and in complex with the anti-ANXA1. Partially overlapping peptic peptides are numbered from the N- to the C-terminus. High confidence identification by mass, retention time and charge was applied using a peptide library. (b) Heat map of anti-ANXA1 antibody epitope regions narrowed down after combining the data from all overlapping peptides and time points.

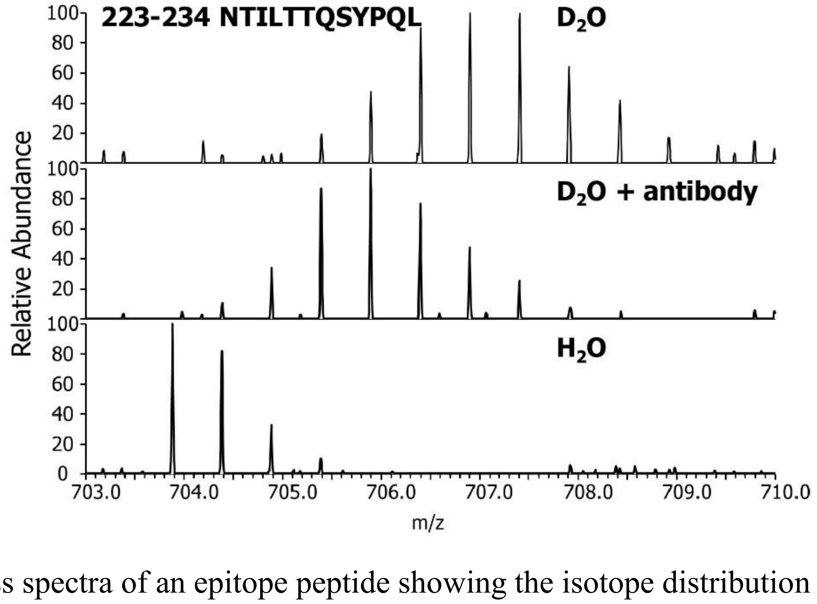


Figure S4: Mass spectra of an epitope peptide showing the isotope distribution of the control (lower panel), ANXA1 in complex with antibody (middle panel) and alone after 24 h HDX (top panel).

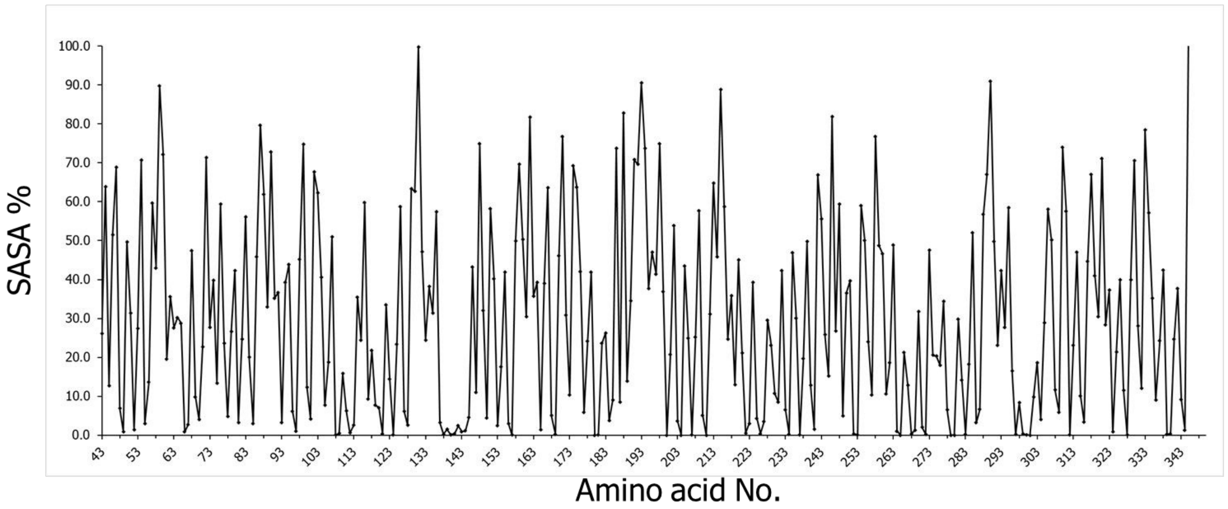


Figure S5: Relative solvent accessibility surface area (SASA) of porcine ANXA1 PDBe: 1MCX. Total SASA of each residue was calculated using PyMOL. The exposure of the residue X was then normalized to the accessibility of a GxG peptides [Miller S, Janin J, Lesk AM, Chothia C. Interior and Surface of Monomeric Proteins. J. Mol. Biol. 1987 196, 641-656]. The first 42 residues are not resolved in crystal structure. Amino acid numbers are adapted to the sequence used within this experiments.

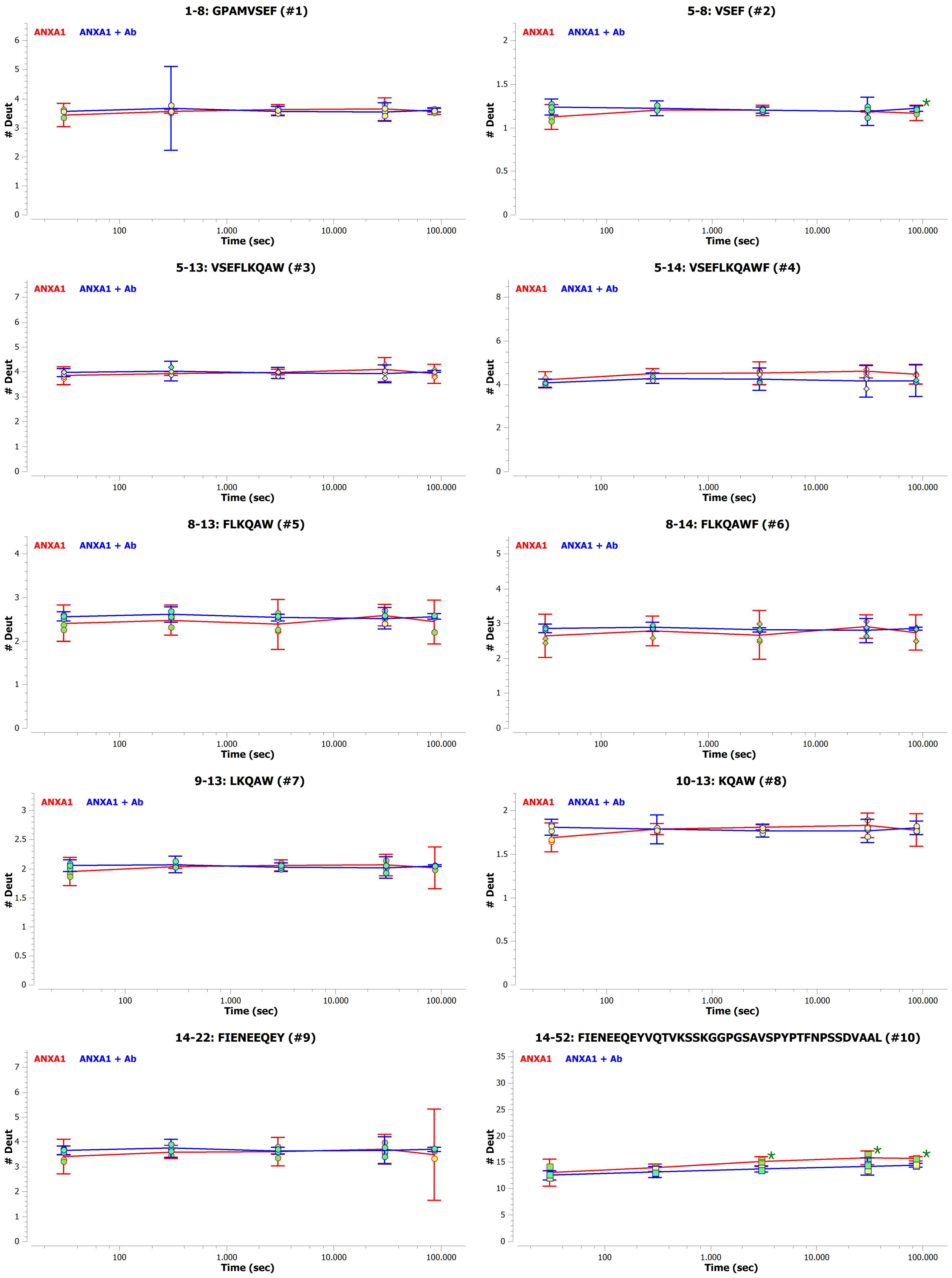

1-8: GPAMVSEF (#1)
5-8: VSEF (#2)
5-13: VSEFLKQAW (#3)
5-14: VSEFLKQAWF (#4)
8-13: FLKQAW (#5)
8-14: FLKQAWF (#6)
9-13: LKQAW (#7)
10-13: KQAW (#8)
14-22: FIENEEQEY (#9)
14-52: FIENEEQEYVQTVKSSKGGPGSAVSPYPTFNPSSDVAAL (#10)
ANXA1
ANXA1 + Ab
# Deut
Time (sec)
100
1.000
10.000
100.000

**14-57: FIENEEQEYVQTVKSSKGGPGSAVSPYPTFNPSSDVAALHKAIM (#11)**

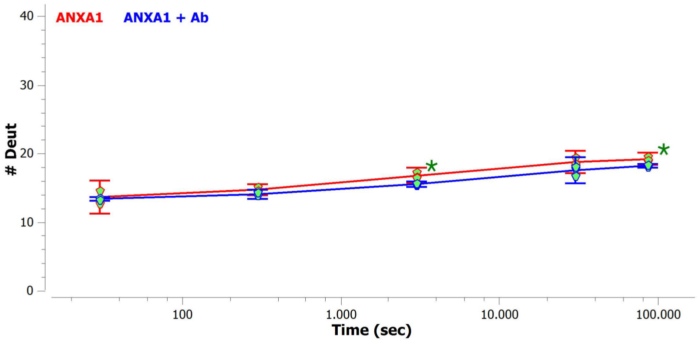


**14-65: FIENEEQEYVQTVKSSKGGPGSAVSPYPTFNPSSDVAALHKAIMVKGVDEAT (#12)**

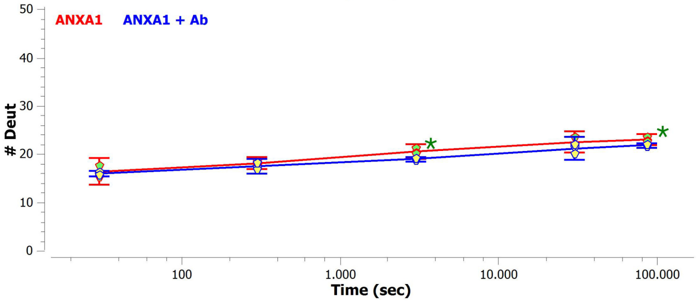


**14-68: FIENEEQEYVQTVKSSKGGPGSAVSPYPTFNPSSDVAALHKAIMVKGVDEATIID (#13)**

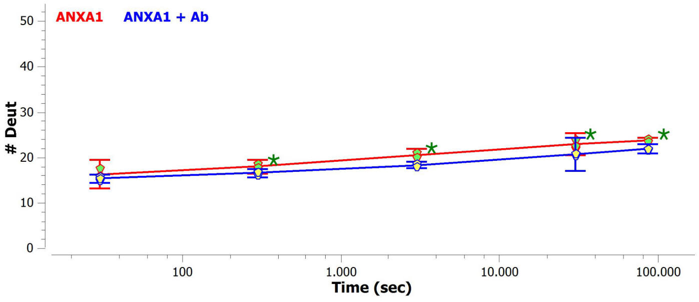


**22-52: YVQTVKSSKGGPGSAVSPYPTFNPSSDVAAL (#14)**

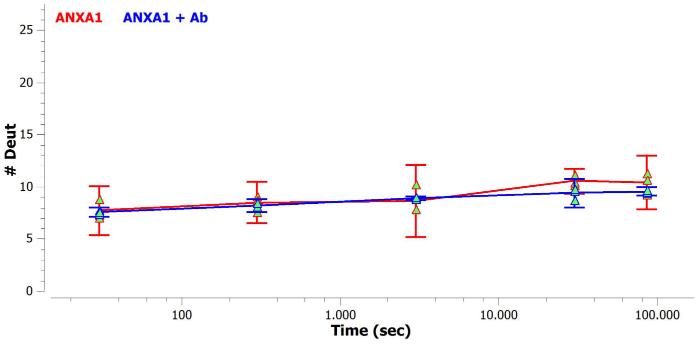


**22-57: YVQTVKSSKGGPGSAVSPYPTFNPSSDVAALHKAIM (#15)**

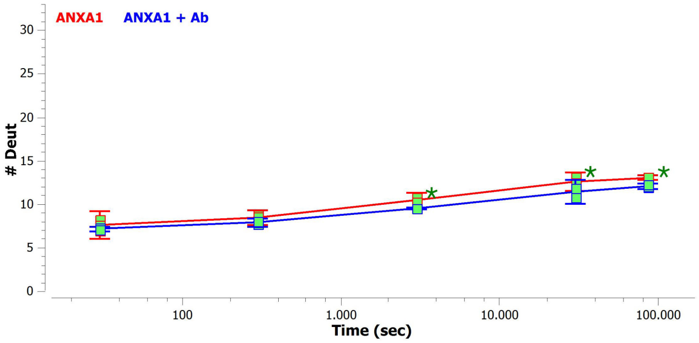


**22-62: YVQTVKSSKGGPGSAVSPYPTFNPSSDVAALHKAIMVKGVD (#16)**

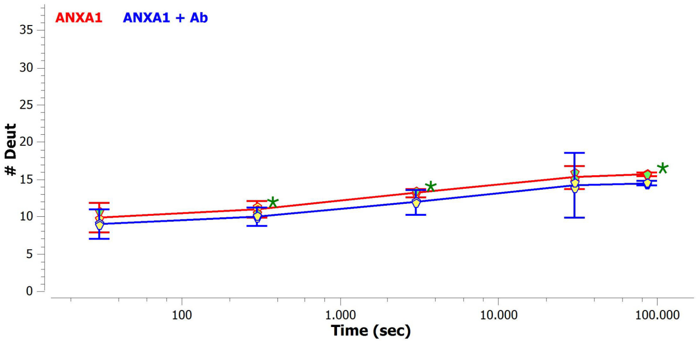


**22-63: YVQTVKSSKGGPGSAVSPYPTFNPSSDVAALHKAIMVKGVDE (#17)**

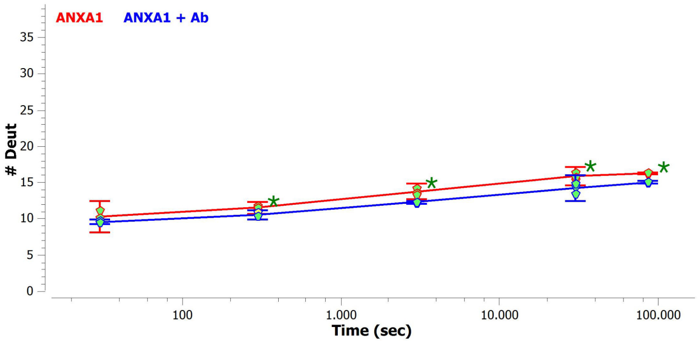


**22-65: YVQTVKSSKGGPGSAVSPYPTFNPSSDVAALHKAIMVKGVDEAT (#18)**

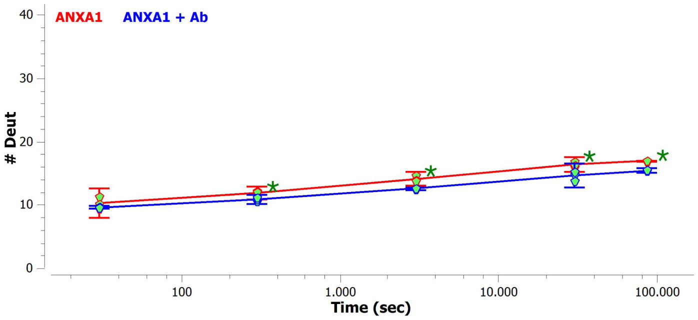


**22-66: YVQTVKSSKGGPGSAVSPYPTFNPSSDVAALHKAIMVKGVDEATI (#19)**

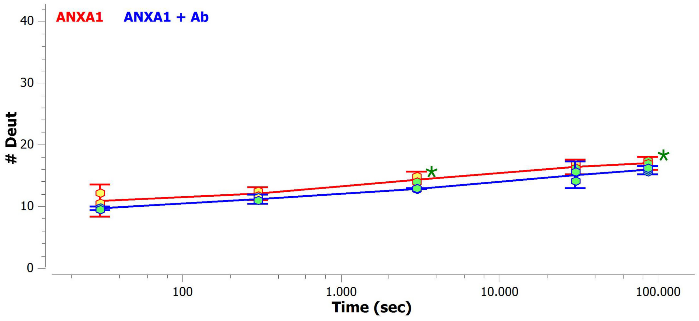


**22-68: YVQTVKSSKGGPGSAVSPYPTFNPSSDVAALHKAIMVKGVDEATIID (#20)**

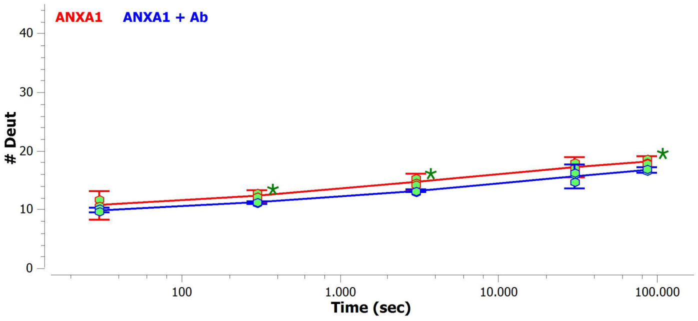

**22-70: YVQTVKSSKGGPGSAVSPYPTFNPSSDVAALHKAIMVKGVDEATIIDIL (#21)**

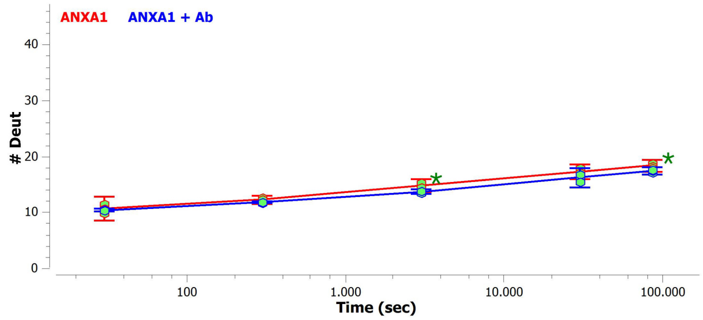


**53-64: HKAIMVKGVDEA (#22)**

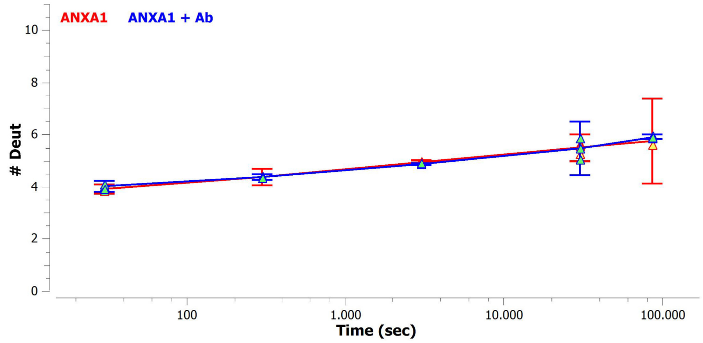


**53-65: HKAIMVKGVDEAT (#23)**

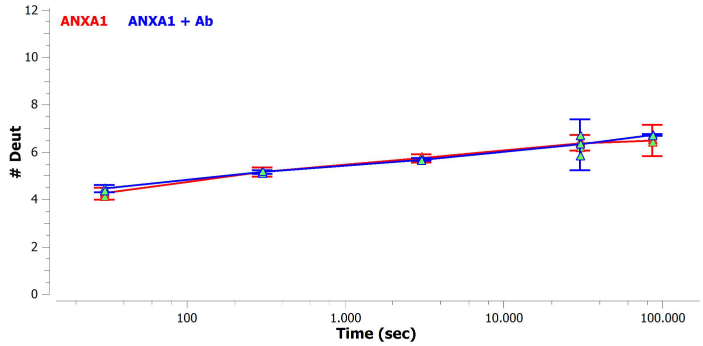


**53-66: HKAIMVKGVDEATI (#24)**

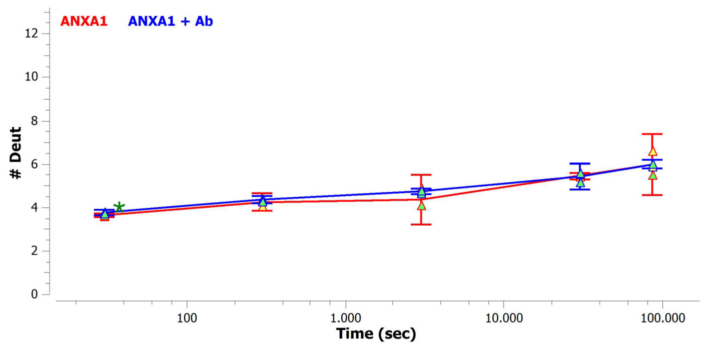


**53-68: HKAIMVKGVDEATIID (#25)**

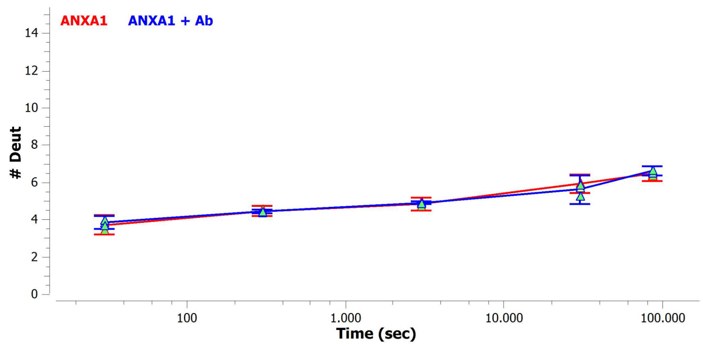


**53-70: HKAIMVKGVDEATIIDIL (#26)**

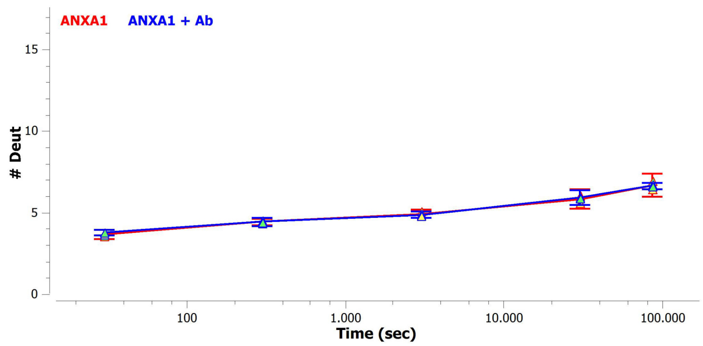


**58-66: VKGVDEATI (#27)**

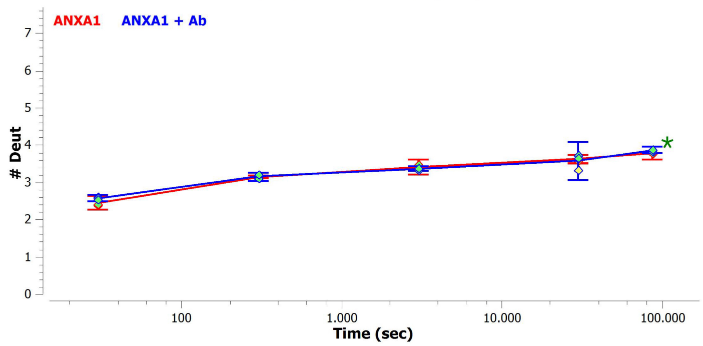


**58-68: VKGVDEATIID (#28)**

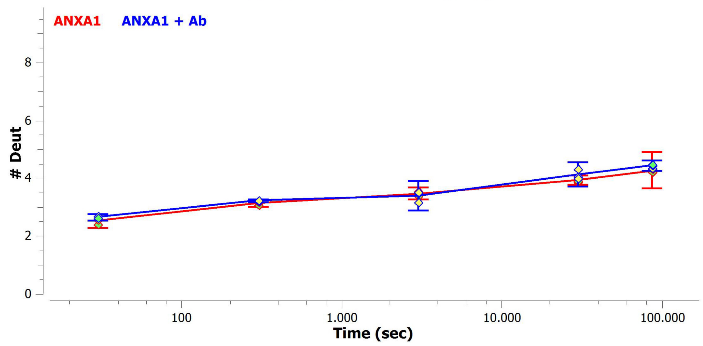


**58-70: VKGVDEATIIDIL (#29)**

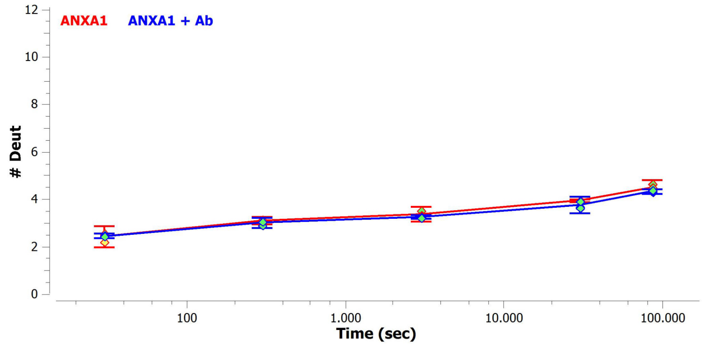


**58-85: VKGVDEATIIDILTKRNNAQRQQIKAAY (#30)**

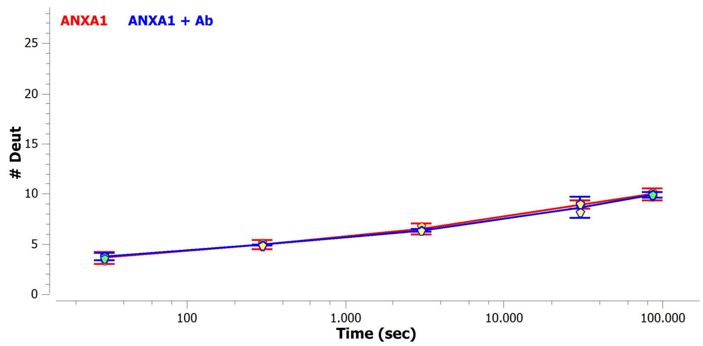

**58-107: VKGVDEATIIDILTKRNNAQRQQIKAAYLQETGKPLDETLKKALTGHLEE (#31)**

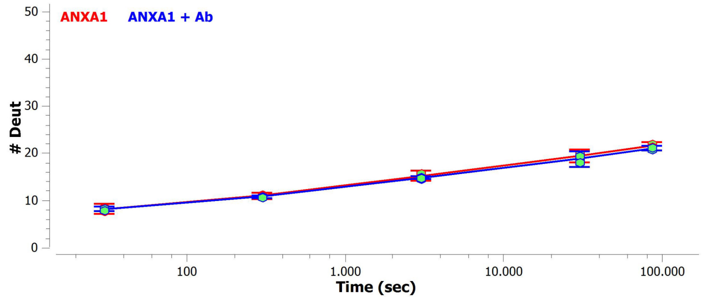


**63-68: EATIID (#32)**

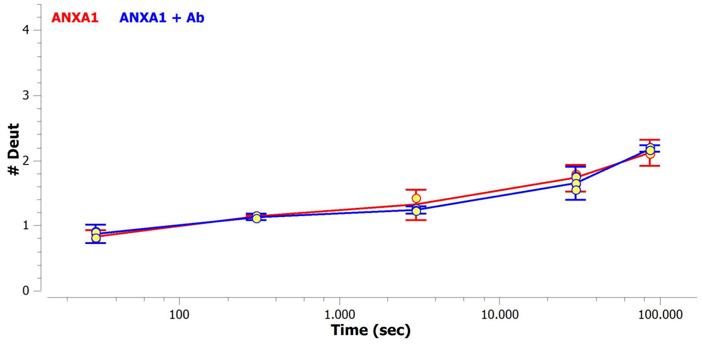


**63-107: EATIIDILTKRNNAQRQQIKAAYLQETGKPLDETLKKALTGHLEE (#33)**

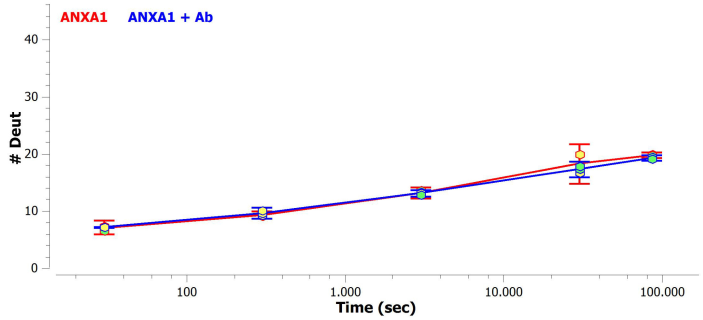


**64-70: ATIIDIL (#34)**

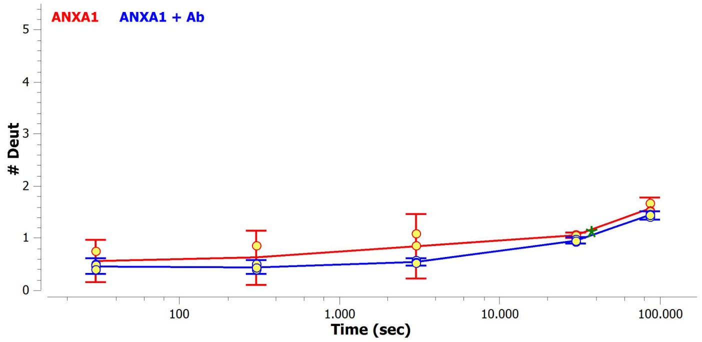


**64-83: ATIIDILTKRNNAQRQQIKA (#35)**

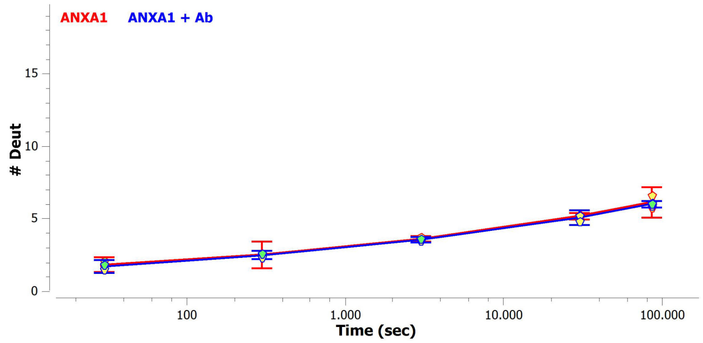


**64-85: ATIIDILTKRNNAQRQQIKAAY (#36)**

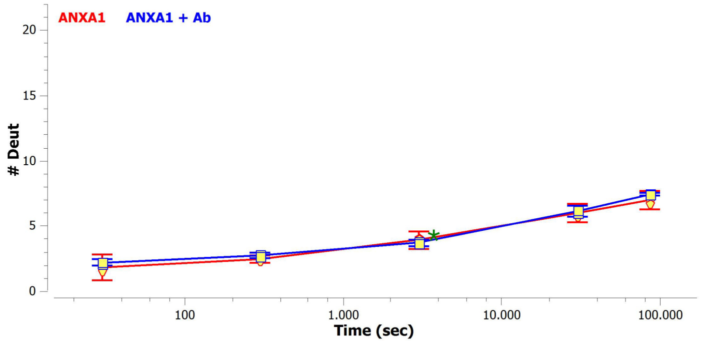


**64-107: ATIIDILTKRNNAQRQQIKAAYLQETGKPLDETLKKALTGHLEE (#37)**

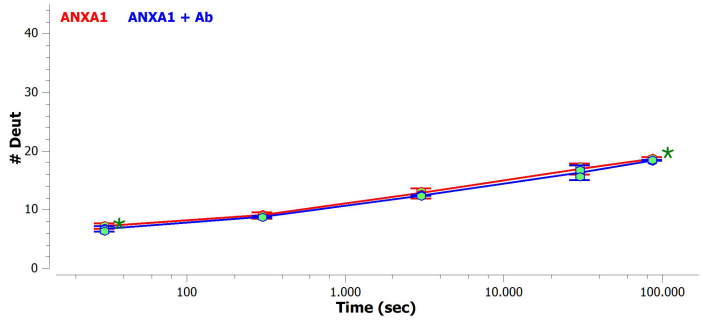


**65-70: TIIDIL (#38)**

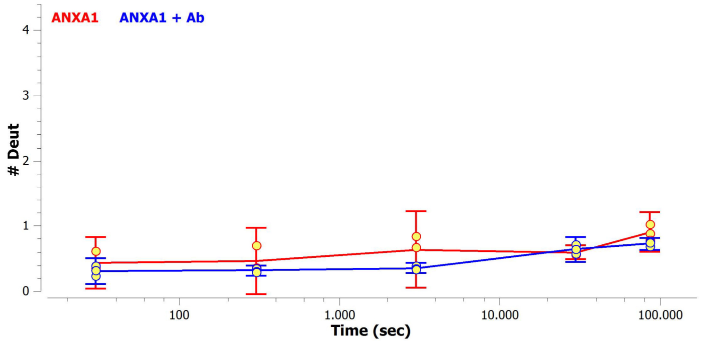


**66-70: IIDIL (#39)**

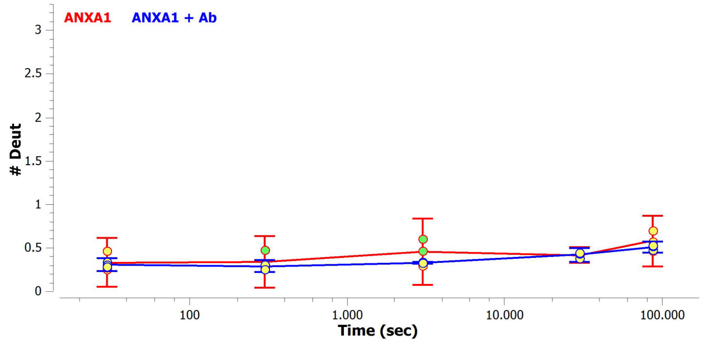


**66-83: IIDILTKRNNAQRQQIKA (#40)**

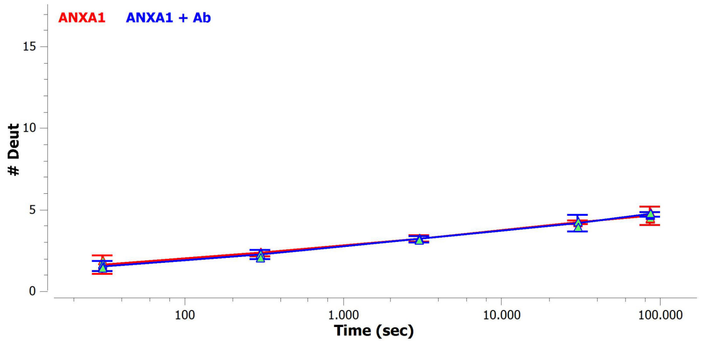

66-85: IIDILTKRNNAQRQQIKAAY (#41)

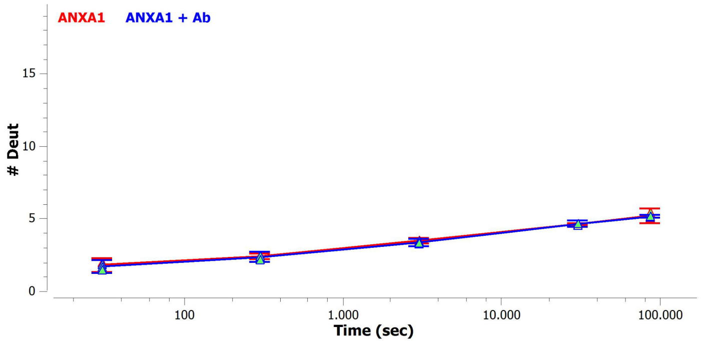


66-86: IIDILTKRNNAQRQQIKAAYL (#42)

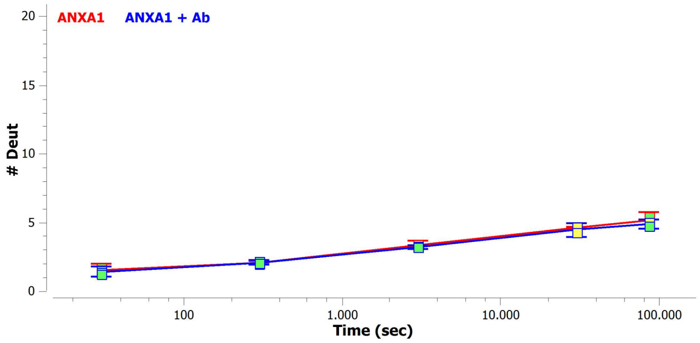


66-107: IIDILTKRNNAQRQQIKAAYLQETGKPLDETLKKALTGHLEE (#43)

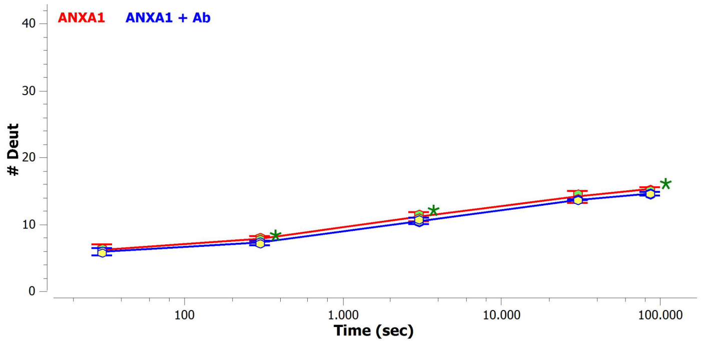


67-70: IDIL (#44)

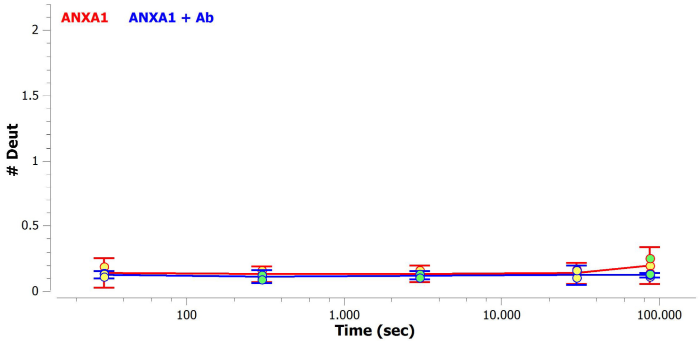


67-83: IDILTKRNNAQRQQIKA (#45)

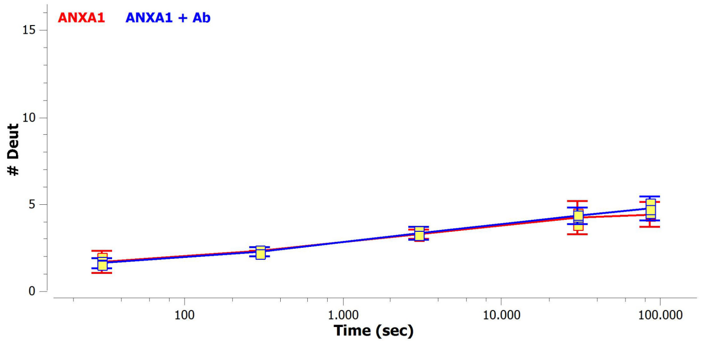


67-85: IDILTKRNNAQRQQIKAAY (#46)

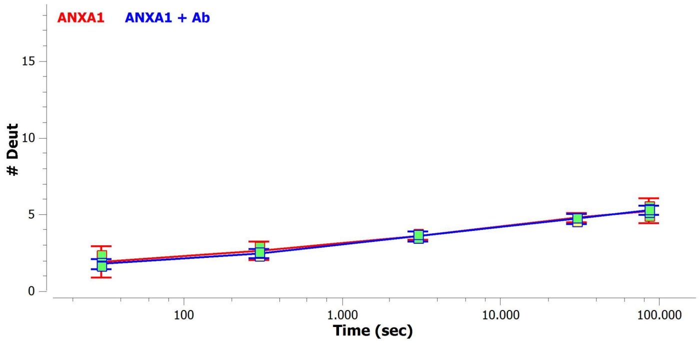


67-86: IDILTKRNNAQRQQIKAAYL (#47)

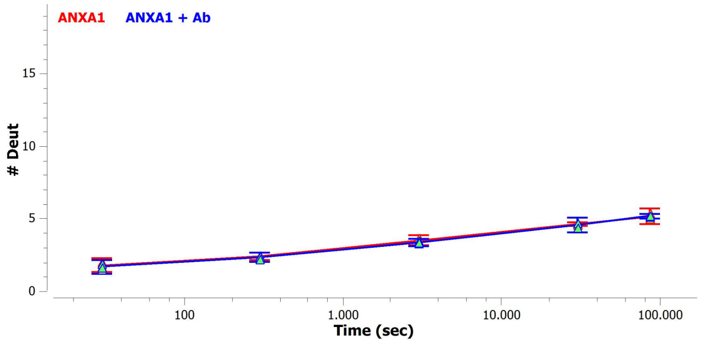


67-107: IDILTKRNNAQRQQIKAAYLQETGKPLDETLKKALTGHLEE (#48)

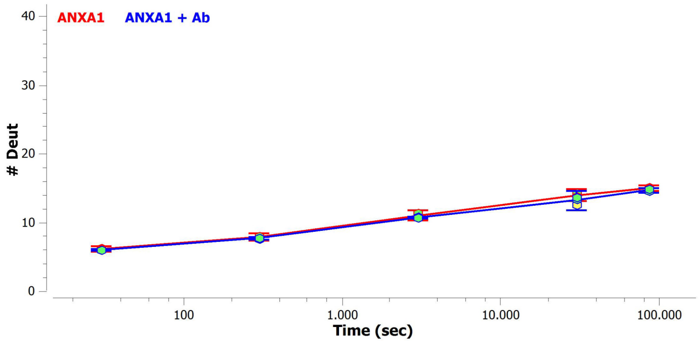


68-86: DILTKRNNAQRQQIKAAYL (#49)

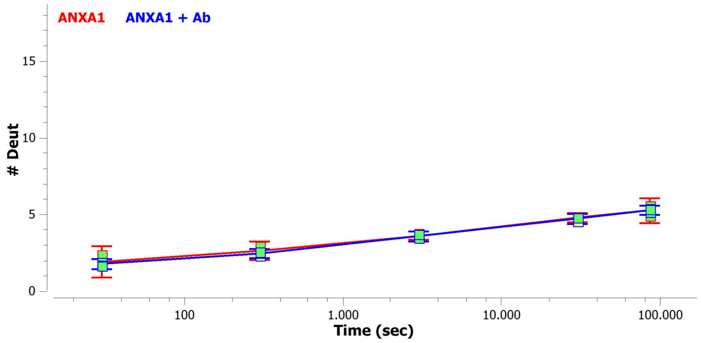


69-85: ILTKRNNAQRQQIKAAY (#50)

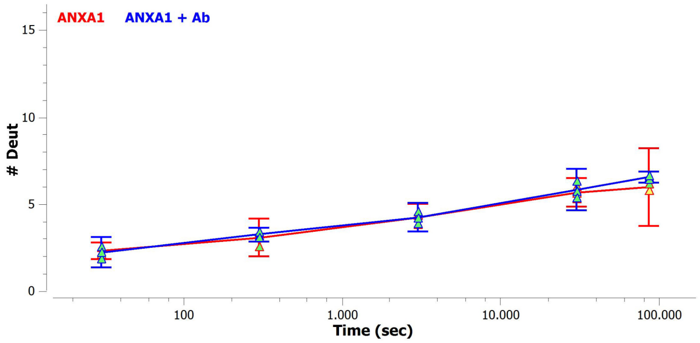

69-86: ILTKRNNAQRQQIKAAYL (#51)

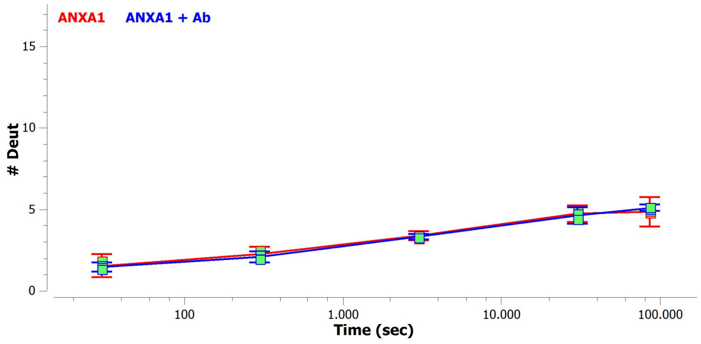


69-97: ILTKRNNAQRQQIKAAYLQETGKPLDETL (#52)

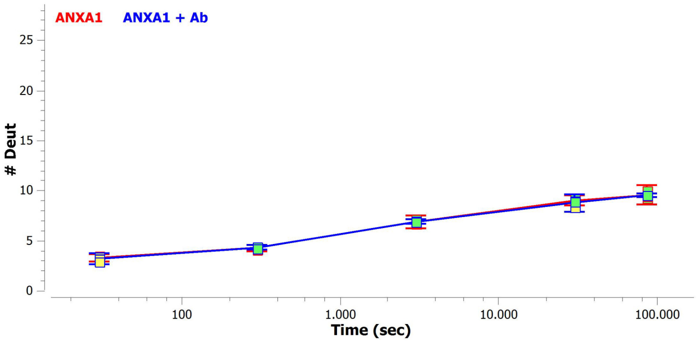


69-107: ILTKRNNAQRQQIKAAYLQETGKPLDETLKKALTGHLEE (#53)

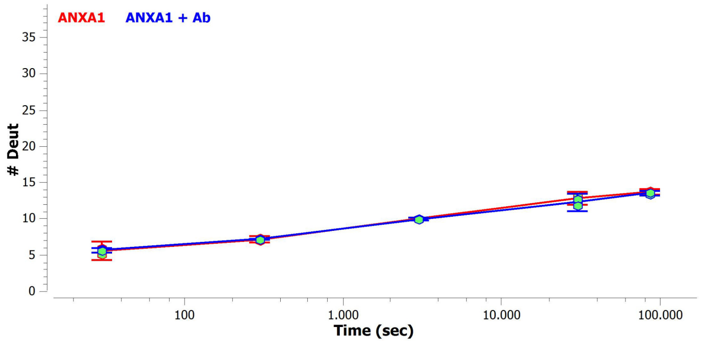


70-86: LTKRNNAQRQQIKAAYL (#54)

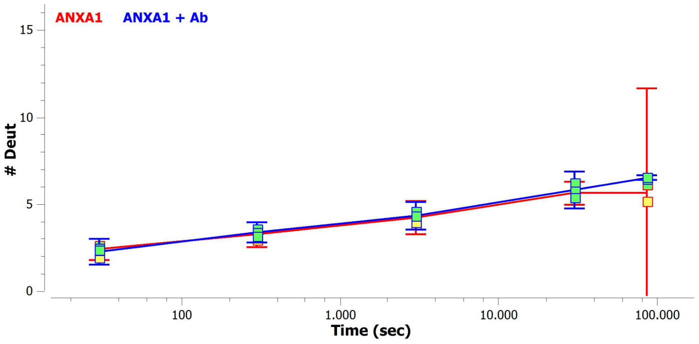


71-86: TKRNNAQRQQIKAAYL (#55)

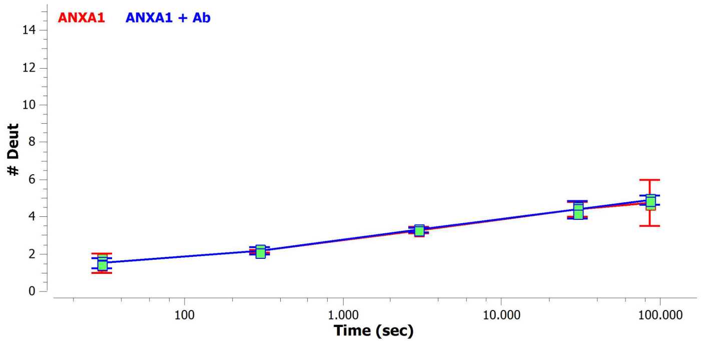


71-97: TKRNNAQRQQIKAAYLQETGKPLDETL (#56)

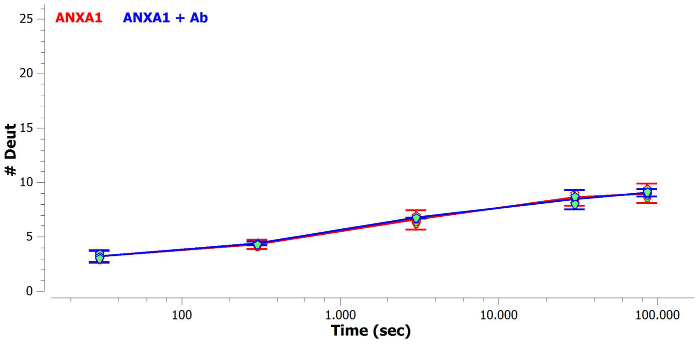


71-107: TKRNNAQRQQIKAAYLQETGKPLDETLKKALTGHLEE (#57)

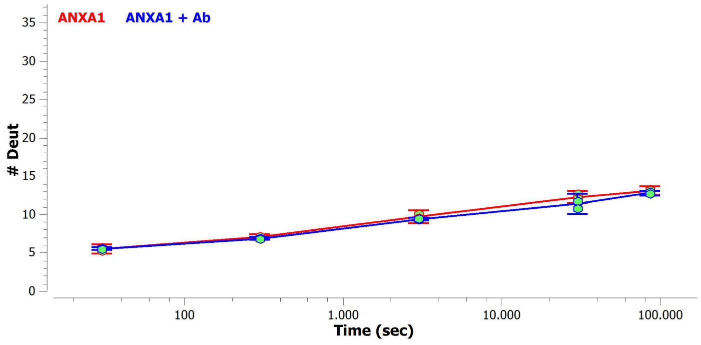


84-107: AYLQETGKPLDETLKKALTGHLEE (#58)

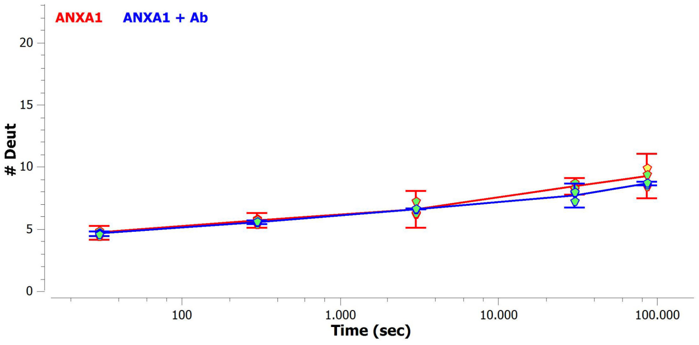


86-97: LQETGKPLDETL (#59)

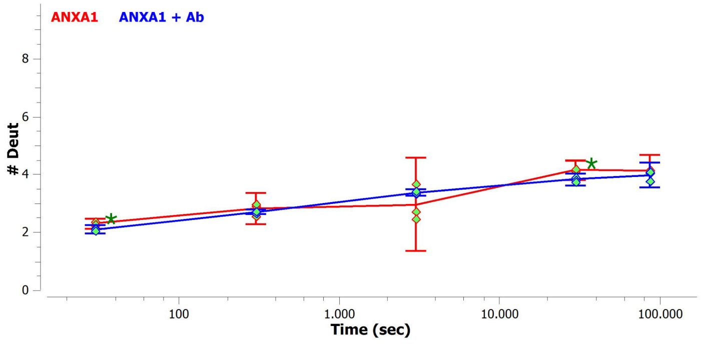


86-107: LQETGKPLDETLKKALTGHLEE (#60)

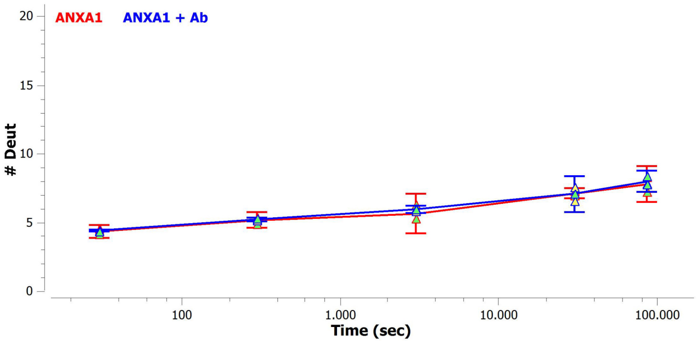

**86-110: LQETGKPLDETLKKALTGHLEEVVL (#61)**

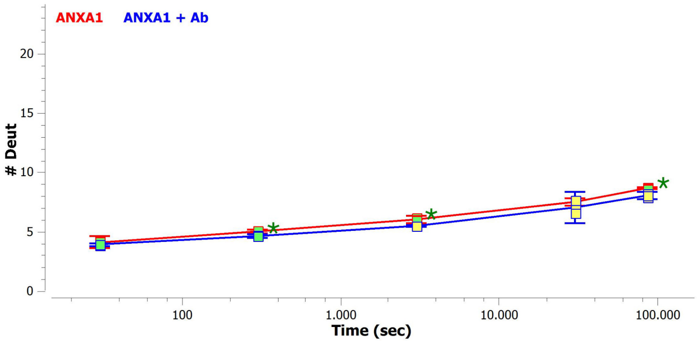


**86-112: LQETGKPLDETLKKALTGHLEEVVLAL (#62)**

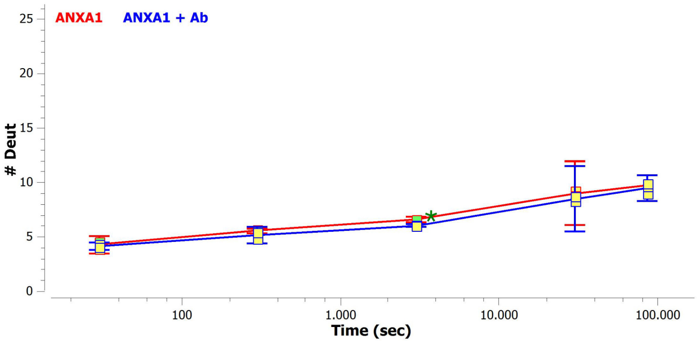


**86-138: LQETGKPLDETLKKALTGHLEEVVLALLKTPAQFDADELRAAMKGLGTDEDTL (#63)**

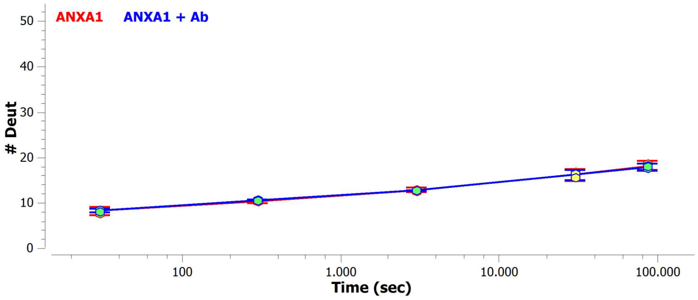


**87-107: QETGKPLDETLKKALTGHLEE (#64)**

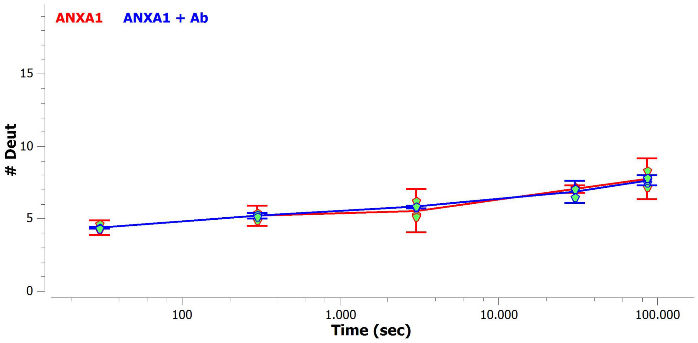


**108-112: VVLAL (#65)**

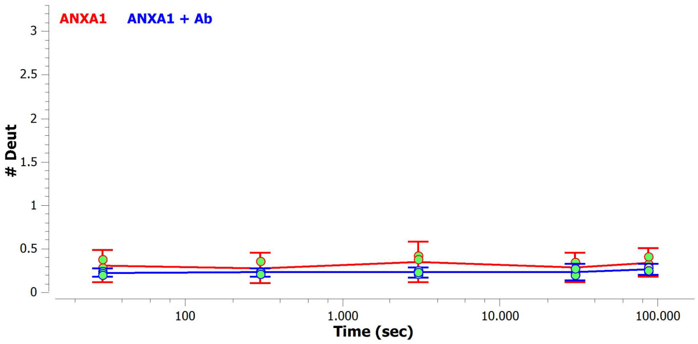


**108-138: VVLALLKTPAQFDADELRAAMKGLGTDEDTL (#66)**

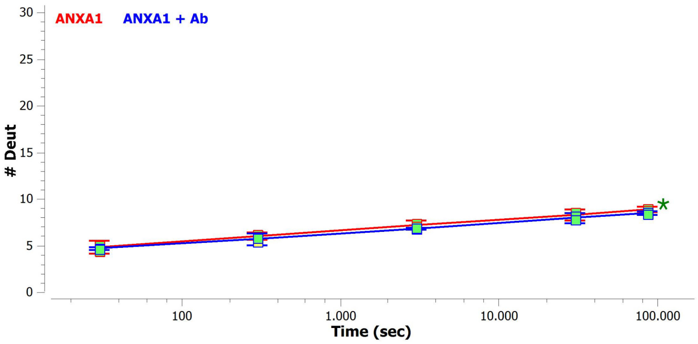


**110-138: LALLKTPAQFDADELRAAMKGLGTDEDTL (#67)**

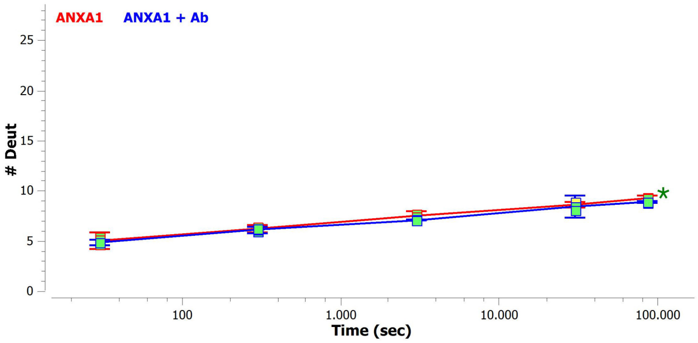


**111-118: ALLKTPAQ (#68)**

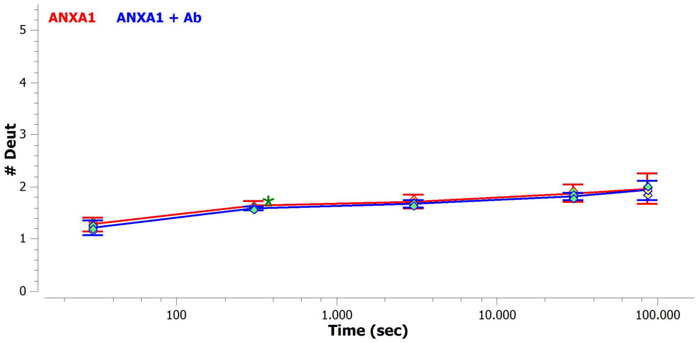


**111-119: ALLKTPAQF (#69)**

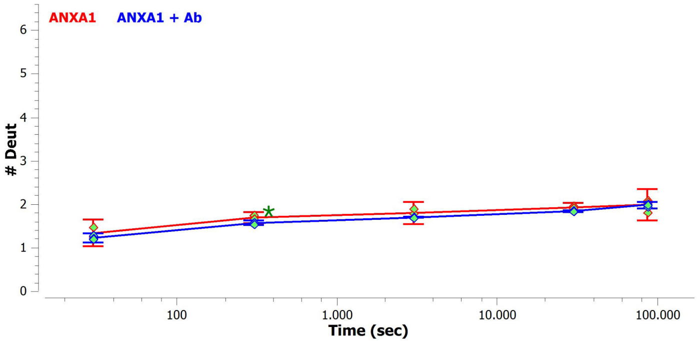


**111-123: ALLKTPAQFDADE (#70)**

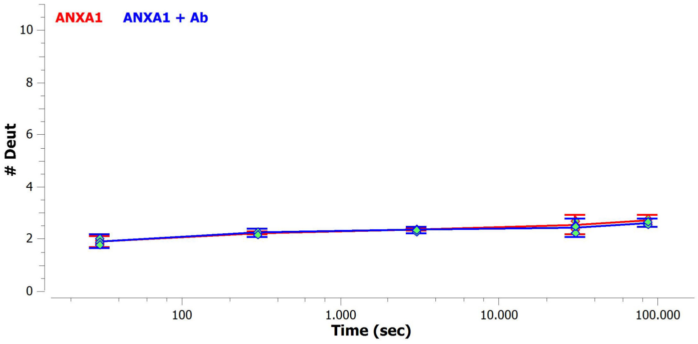

**111-124: ALLKTPAQFDADEL (#71)**

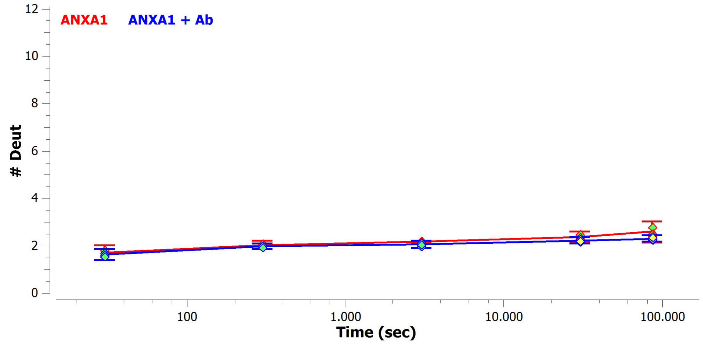


**111-126: ALLKTPAQFDADELRA (#72)**

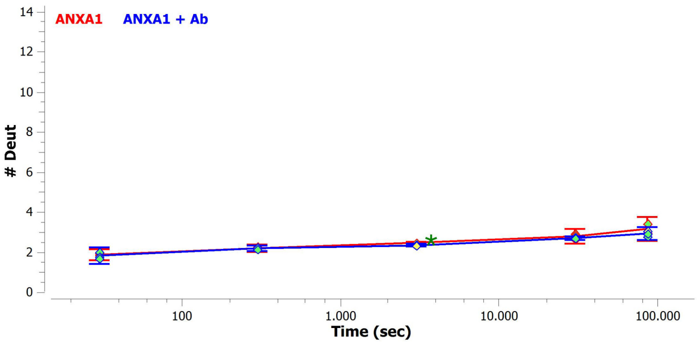


**111-137: ALLKTPAQFDADELRAAMKGLGTDEDT (#73)**

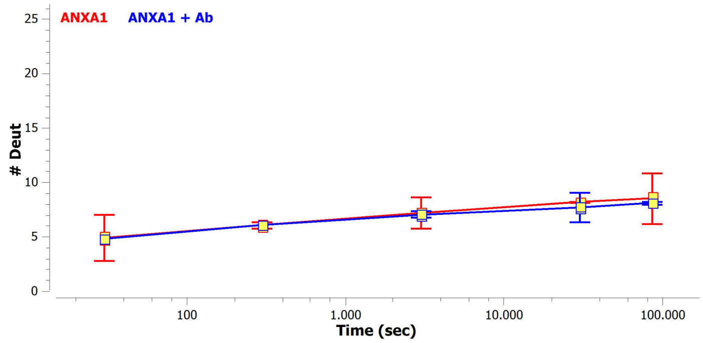


**111-138: ALLKTPAQFDADELRAAMKGLGTDEDTL (#74)**

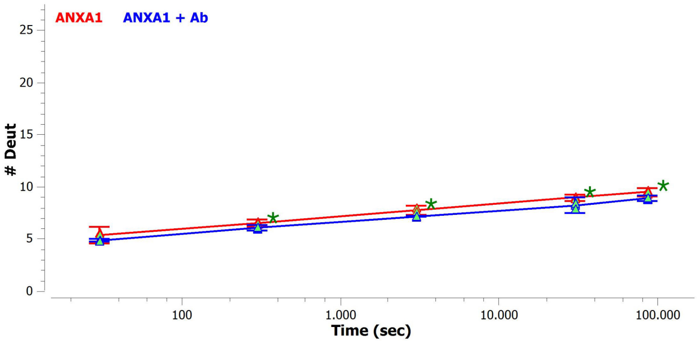


**111-140: ALLKTPAQFDADELRAAMKGLGTDEDTLIE (#75)**

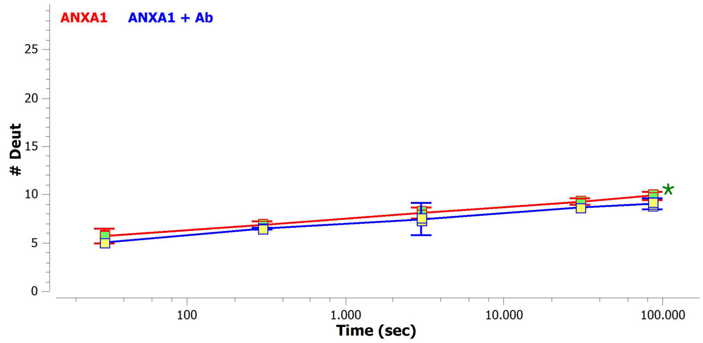


**113-123: LKTPAQFDADE (#76)**

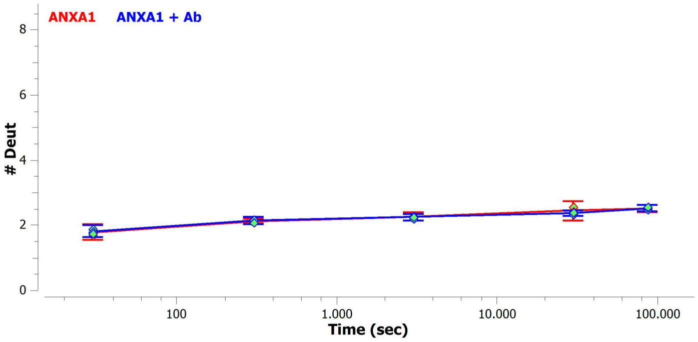


**113-124: LKTPAQFDADEL (#77)**

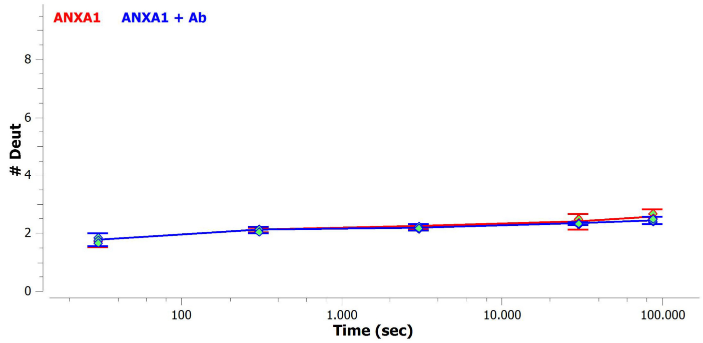


**113-126: LKTPAQFDADELRA (#78)**

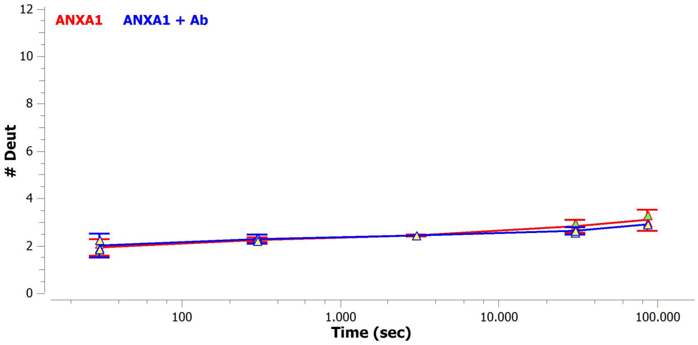


**113-137: LKTPAQFDADELRAAMKGLGTDEDT (#79)**

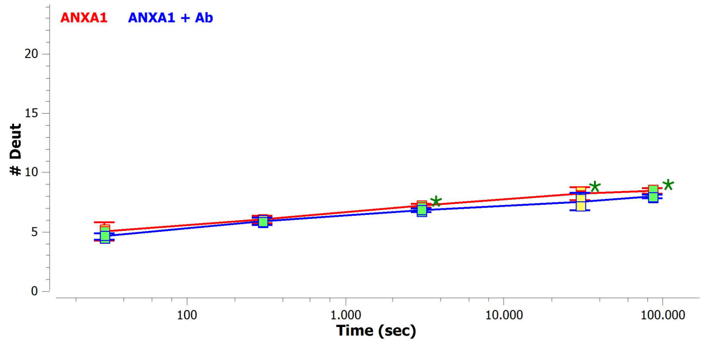


**113-138: LKTPAQFDADELRAAMKGLGTDEDTL (#80)**

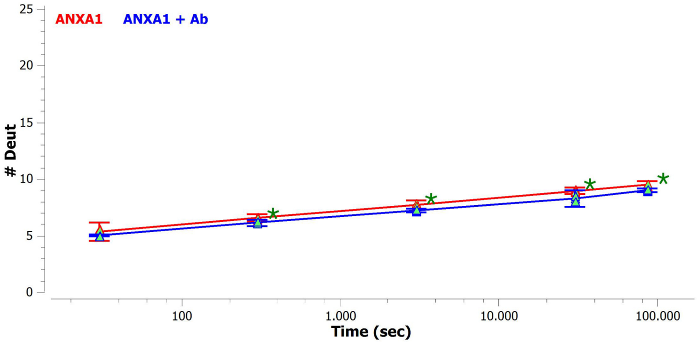

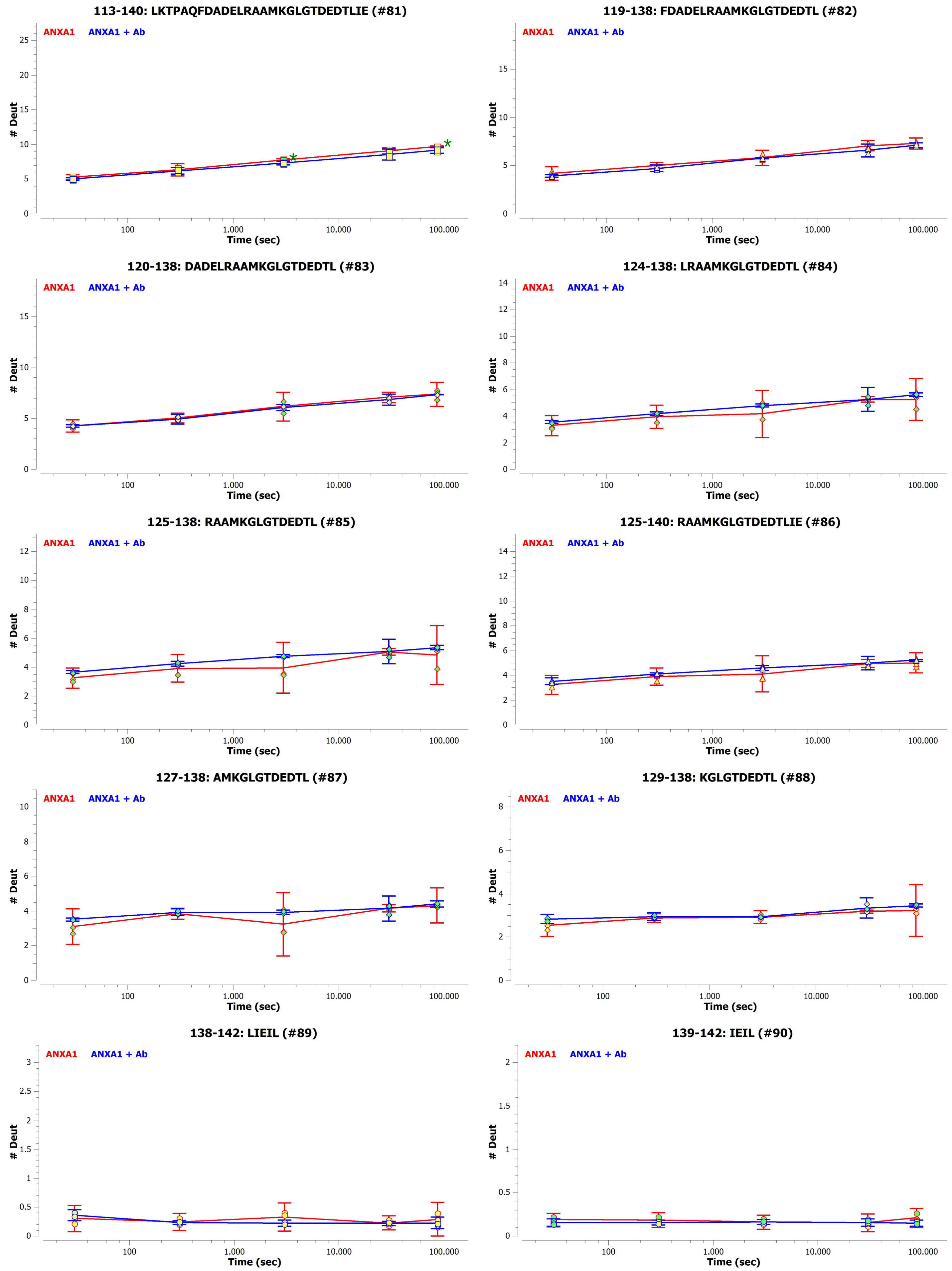
113-140: LKTPAQFDADELRAAMKGLGTDEDTLIE (#81)
ANXA1
ANXA1 + Ab
# Deut
Time (sec)
119-138: FDADELRAAMKGLGTDEDTL (#82)
ANXA1
ANXA1 + Ab
# Deut
Time (sec)
120-138: DADELRAAMKGLGTDEDTL (#83)
ANXA1
ANXA1 + Ab
# Deut
Time (sec)
124-138: LRAAMKGLGTDEDTL (#84)
ANXA1
ANXA1 + Ab
# Deut
Time (sec)
125-138: RAAMKGLGTDEDTL (#85)
ANXA1
ANXA1 + Ab
# Deut
Time (sec)
125-140: RAAMKGLGTDEDTLIE (#86)
ANXA1
ANXA1 + Ab
# Deut
Time (sec)
127-138: AMKGLGTDEDTL (#87)
ANXA1
ANXA1 + Ab
# Deut
Time (sec)
129-138: KGLGTDEDTL (#88)
ANXA1
ANXA1 + Ab
# Deut
Time (sec)
138-142: LIEIL (#89)
ANXA1
ANXA1 + Ab
# Deut
Time (sec)
139-142: IEIL (#90)
ANXA1
ANXA1 + Ab
# Deut
Time (sec)
100
1.000
10.000
100.000

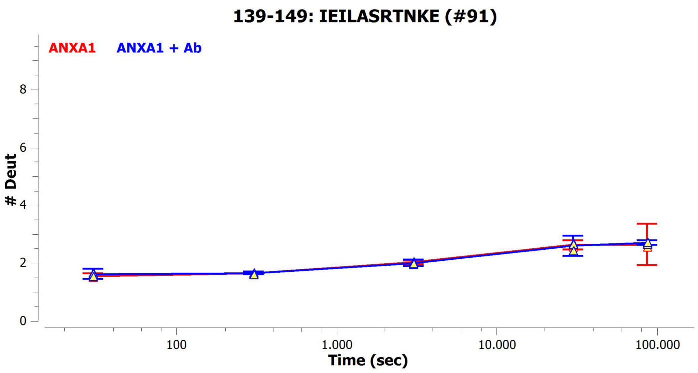
139-149: IEILASRTNKE (#91)
ANXA1
ANXA1 + Ab
# Deut
Time (sec)

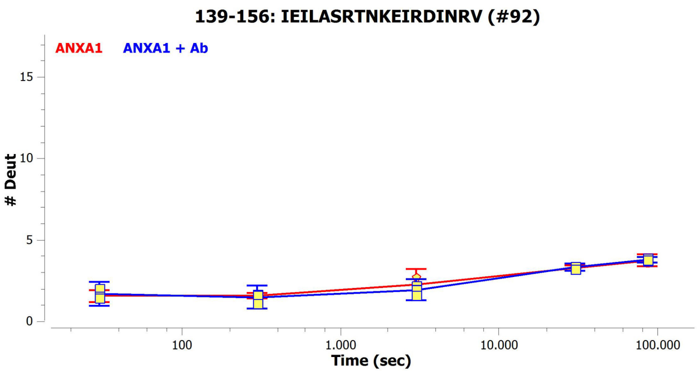
139-156: IEILASRTNKEIRDINRV (#92)
ANXA1
ANXA1 + Ab
# Deut
Time (sec)

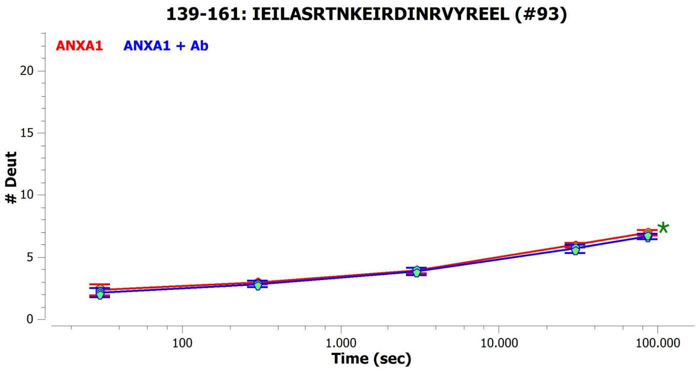
139-161: IEILASRTNKEIRDINRVYREEL (#93)
ANXA1
ANXA1 + Ab
# Deut
Time (sec)

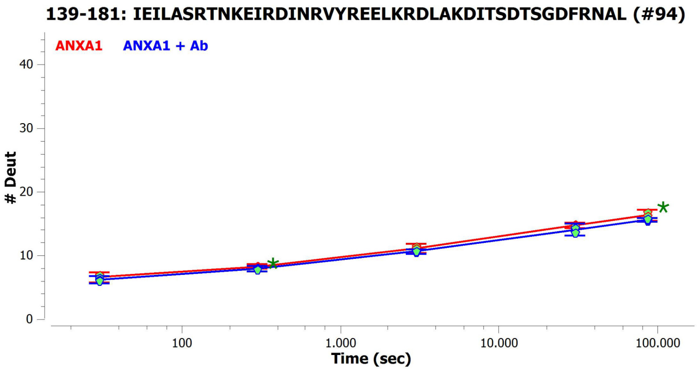
139-181: IEILASRTNKEIRDINRVYREELKRDLAKDITSDTSGDFRNAL (#94)
ANXA1
ANXA1 + Ab
# Deut
Time (sec)

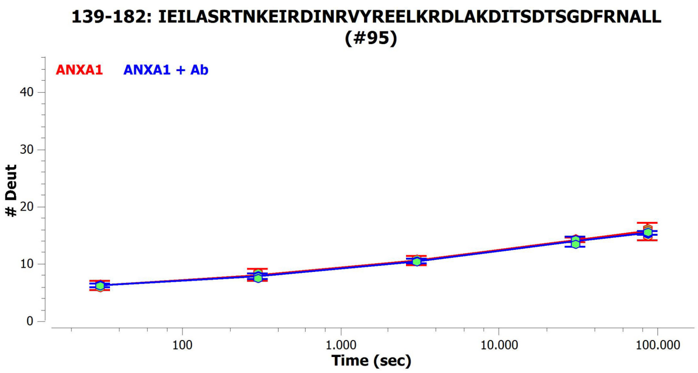
139-182: IEILASRTNKEIRDINRVYREELKRDLAKDITSDTSGDFRNALL (#95)
ANXA1
ANXA1 + Ab
# Deut
Time (sec)

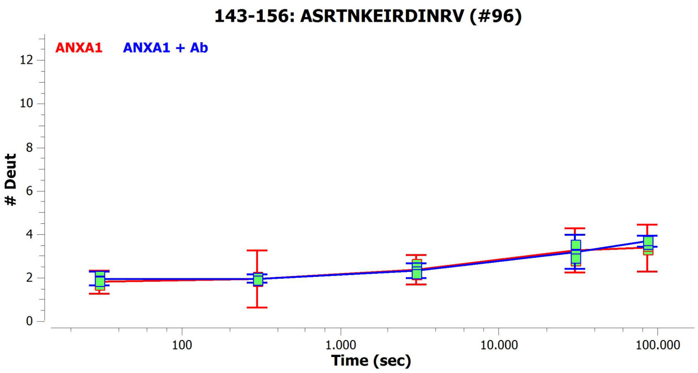
143-156: ASRTNKEIRDINRV (#96)
ANXA1
ANXA1 + Ab
# Deut
Time (sec)

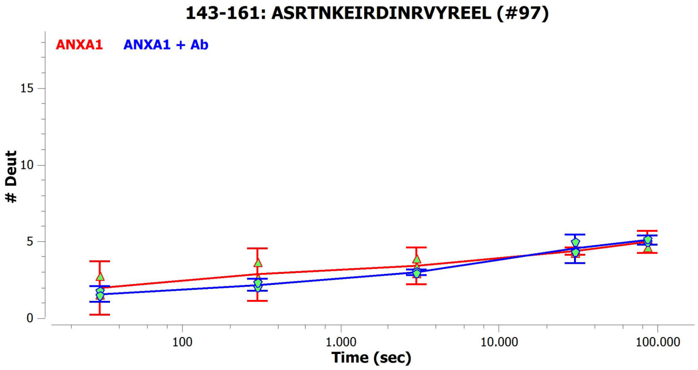
143-161: ASRTNKEIRDINRVYREEL (#97)
ANXA1
ANXA1 + Ab
# Deut
Time (sec)

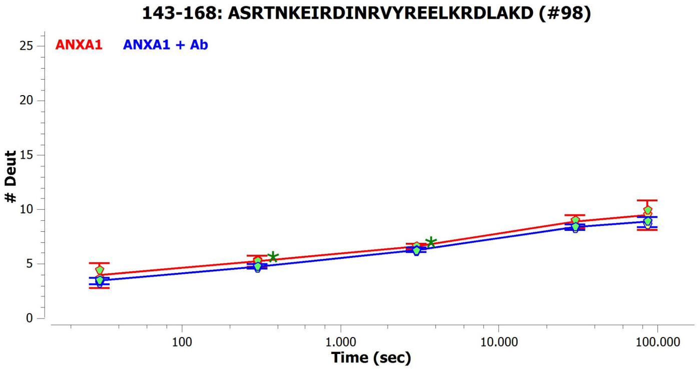
143-168: ASRTNKEIRDINRVYREELKRDLAKD (#98)
ANXA1
ANXA1 + Ab
# Deut
Time (sec)

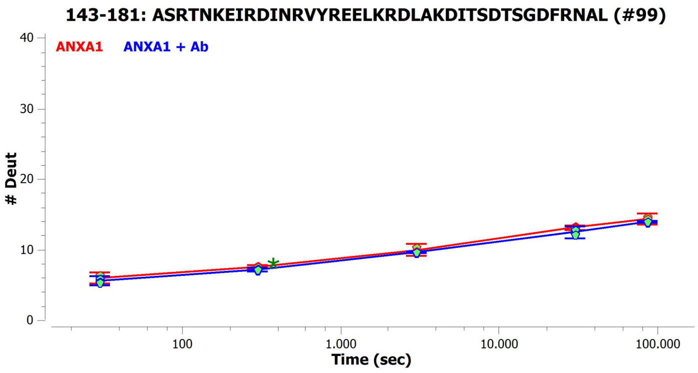
143-181: ASRTNKEIRDINRVYREELKRDLAKDITSDTSGDFRNAL (#99)
ANXA1
ANXA1 + Ab
# Deut
Time (sec)

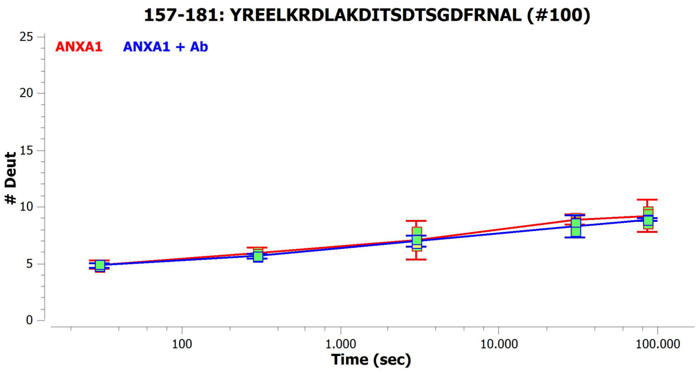
157-181: YREELKRDLAKDITSDTSGDFRNAL (#100)
ANXA1
ANXA1 + Ab
# Deut
Time (sec)

162-181: KRDLAKDITSDTSGDFRNAL (#101)

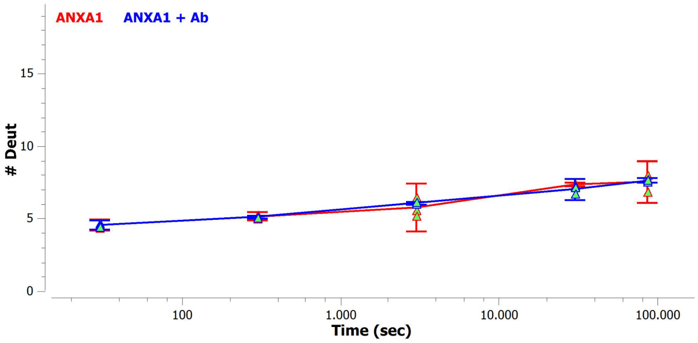


169-181: ITSDTSGDFRNAL (#102)

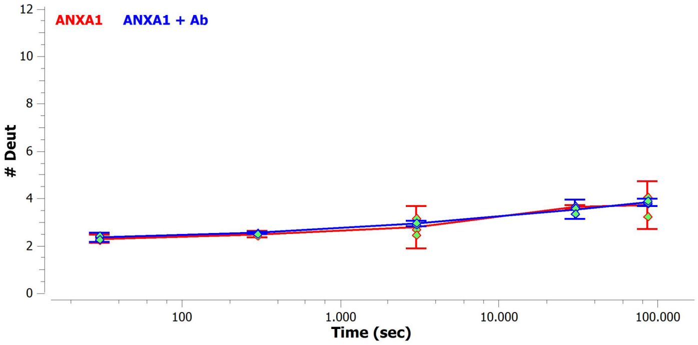


177-181: FRNAL (#103)

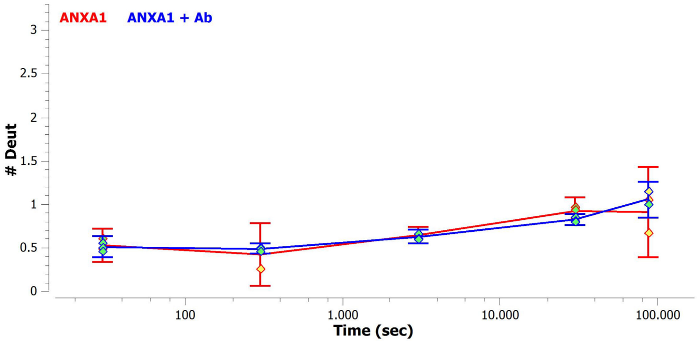


182-199: LSLAKGDRSEDFGVNEDL (#104)

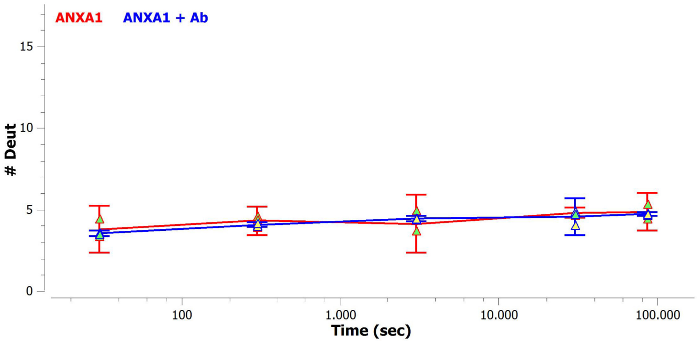


182-203: LSLAKGDRSEDFGVNEDLADSD (#105)

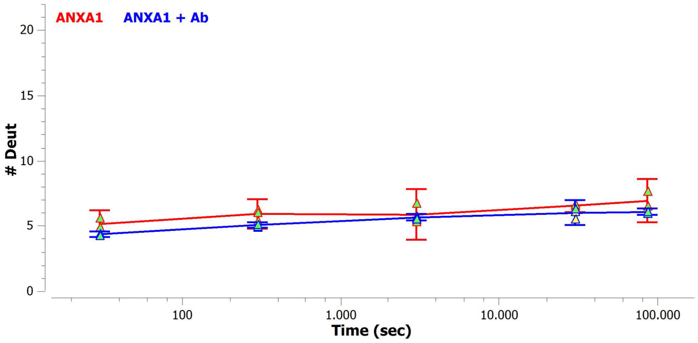


182-204: LSLAKGDRSEDFGVNEDLADSDA (#106)

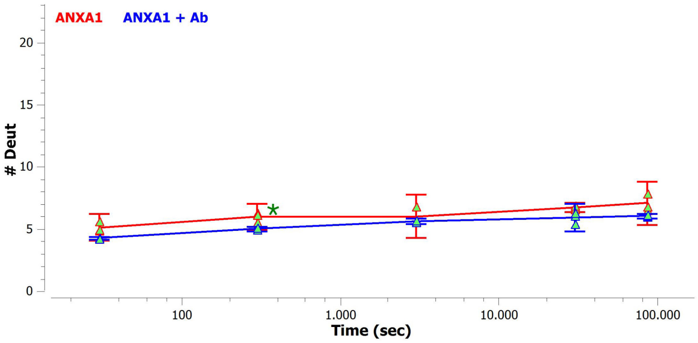


182-206: LSLAKGDRSEDFGVNEDLADSDARA (#107)

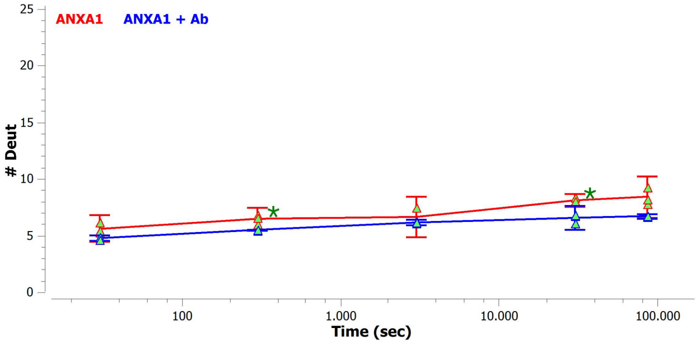


182-207: LSLAKGDRSEDFGVNEDLADSDARAL (#108)

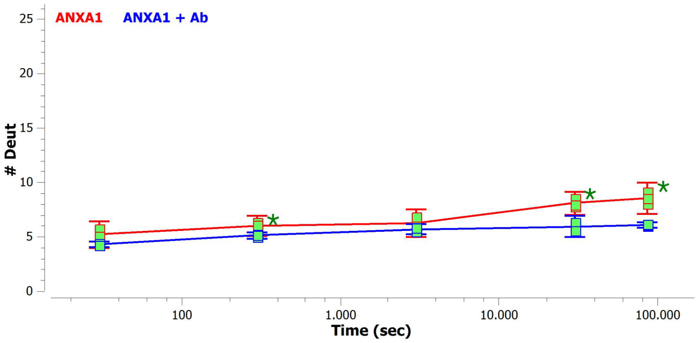


182-221: LSLAKGDRSEDFGVNEDLADSDARALYEAGERRKGTDVNV (#109)

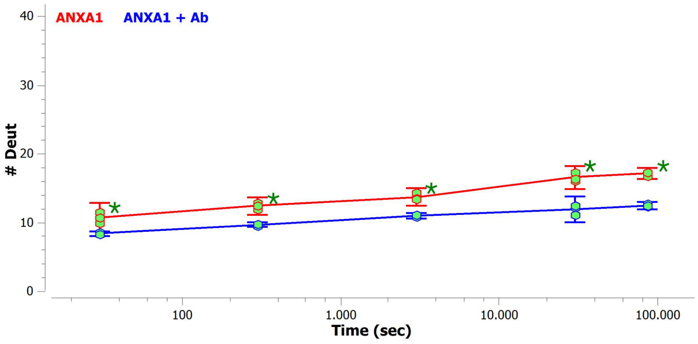


182-222: LSLAKGDRSEDFGVNEDLADSDARALYEAGERRKGTDVNVF (#110)

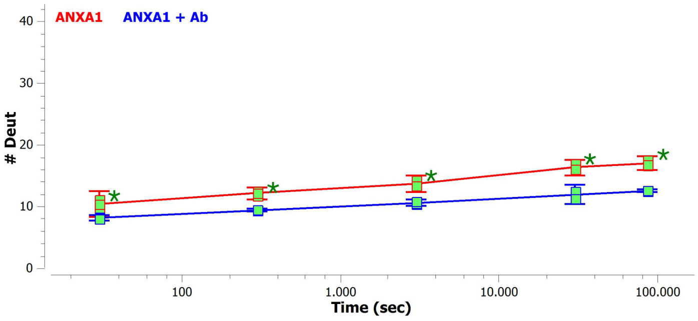

182-226: LSLAKGDRSEDFGVNEDLADSDARALYEAGERRKGTDVNVFNTIL (#111)

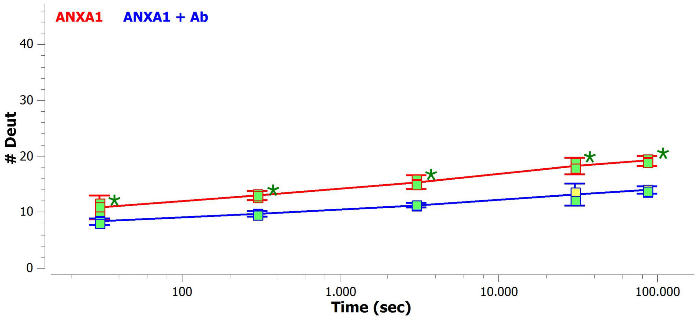


183-204: SLAKGDRSEDFGVNEDLADSDA (#112)

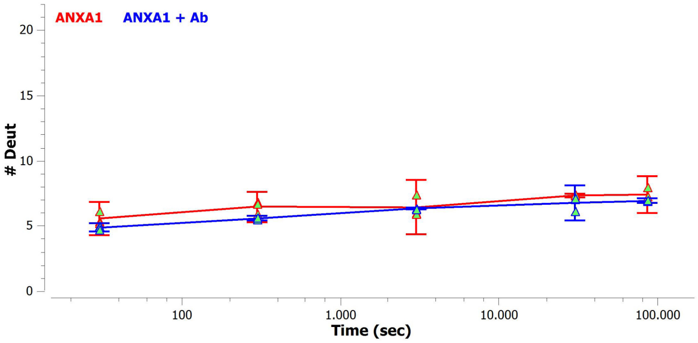


183-206: SLAKGDRSEDFGVNEDLADSDARA (#113)

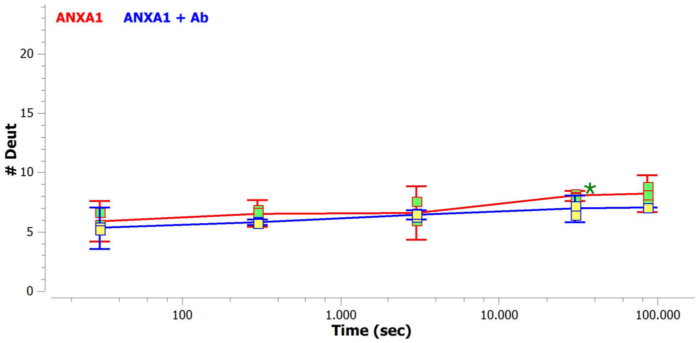


183-222: SLAKGDRSEDFGVNEDLADSDARALYEAGERRKGTDVNVF (#114)

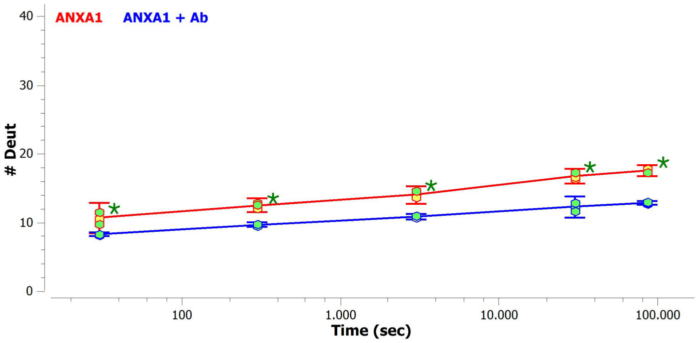


185-204: AKGDRSEDFGVNEDLADSDA (#115)

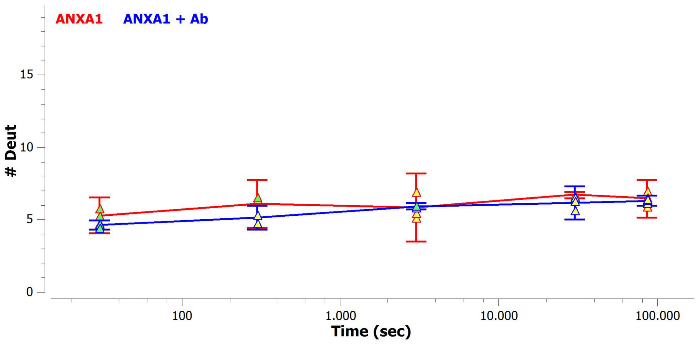


185-222: AKGDRSEDFGVNEDLADSDARALYEAGERRKGTDVNVF (#116)

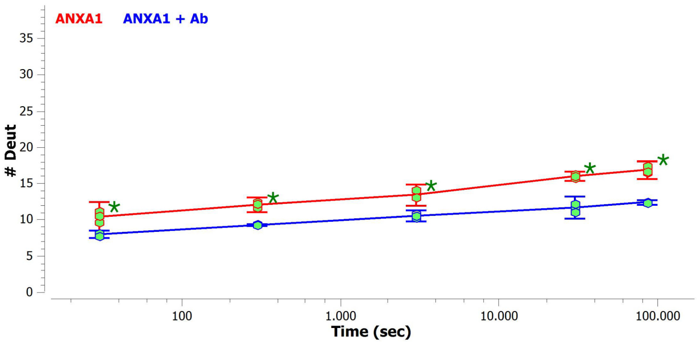


185-226: AKGDRSEDFGVNEDLADSDARALYEAGERRKGTDVNVFNTIL (#117)

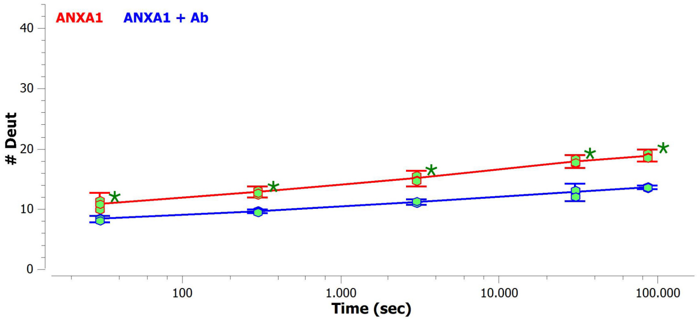


193-206: FGVNEDLADSDARA (#118)

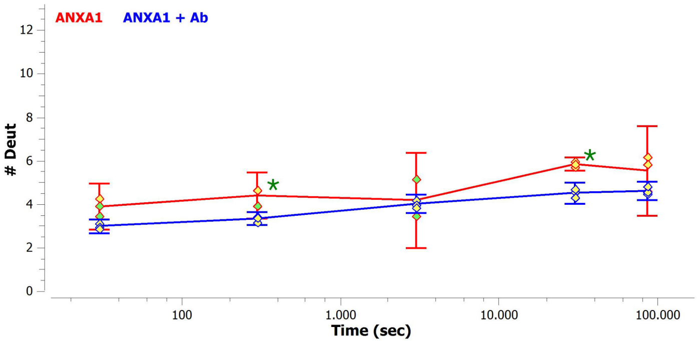


193-226: FGVNEDLADSDARALYEAGERRKGTDVNVFNTIL (#119)

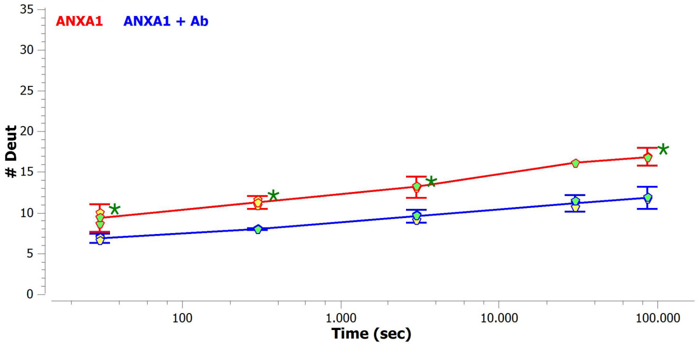


197-226: EDLADSDARALYEAGERRKGTDVNVFNTIL (#120)

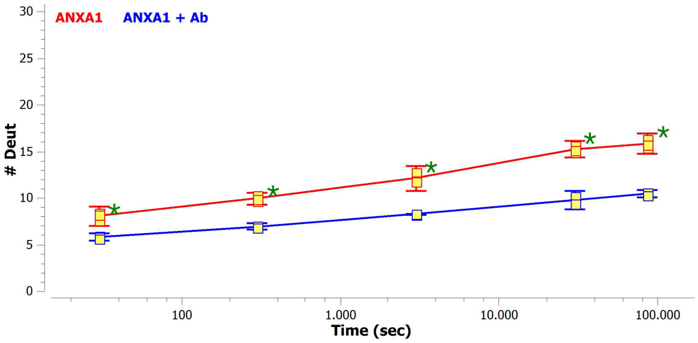

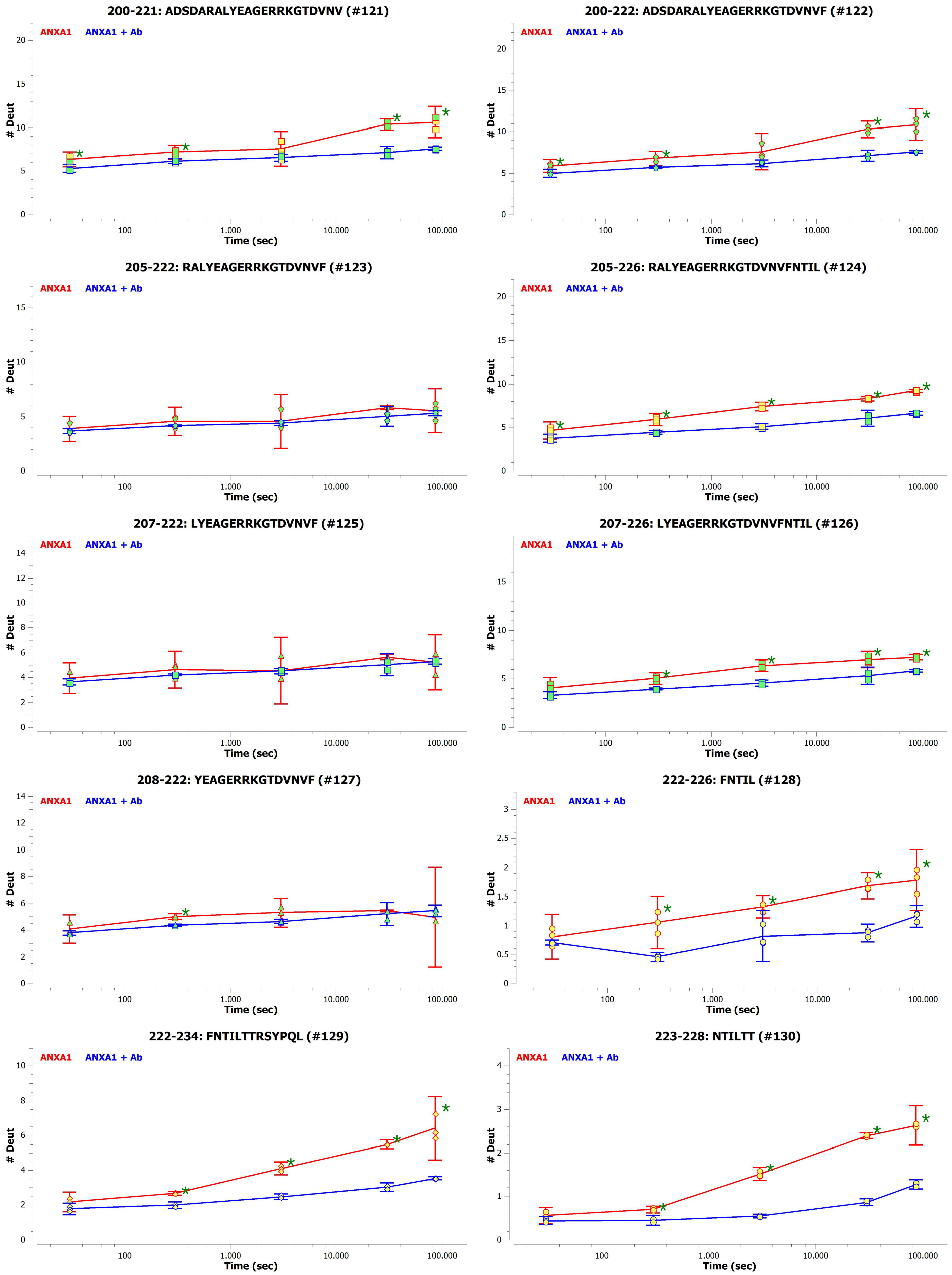
200-221: ADSDARALYEAGERRKGTDVNV (#121)
200-222: ADSDARALYEAGERRKGTDVNVF (#122)
205-222: RALYEAGERRKGTDVNVF (#123)
205-226: RALYEAGERRKGTDVNVFNTIL (#124)
207-222: LYEAGERRKGTDVNVF (#125)
207-226: LYEAGERRKGTDVNVFNTIL (#126)
208-222: YEAGERRKGTDVNVF (#127)
222-226: FNTIL (#128)
222-234: FNTILTTRSYPQL (#129)
223-228: NTILTT (#130)
ANXA1
ANXA1 + Ab
# Deut
Time (sec)
100
1.000
10.000
100.000

223-234: NTILTTRSYPQL (#131)

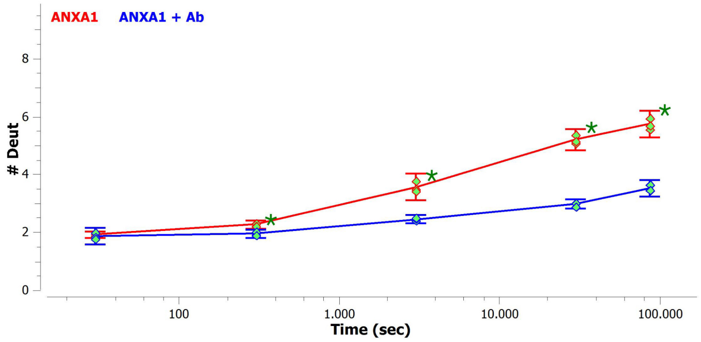


223-257: NTILTTRSYPQLRRVFQKYTKYSKHDMNKVLDLEL (#132)

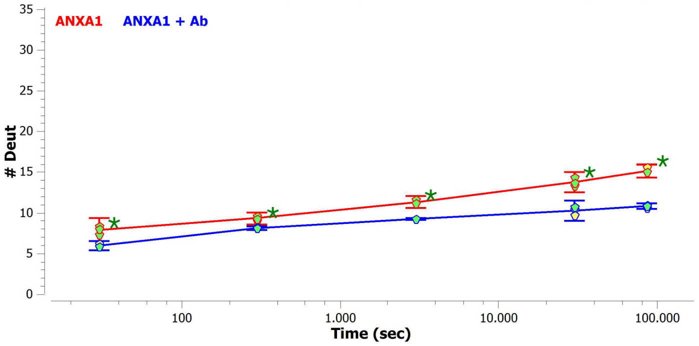


227-234: TTRSYPQL (#133)

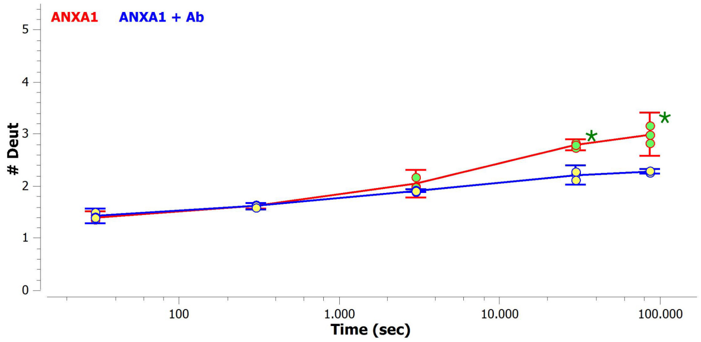


227-254: TTRSYPQLRRVFQKYTKYSKHDMNKVLD (#134)

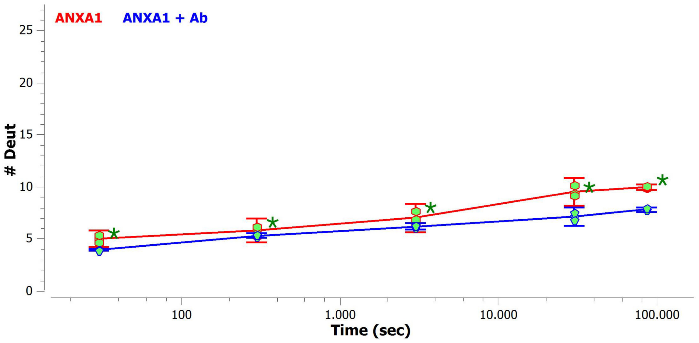


227-255: TTRSYPQLRRVFQKYTKYSKHDMNKVLDL (#135)

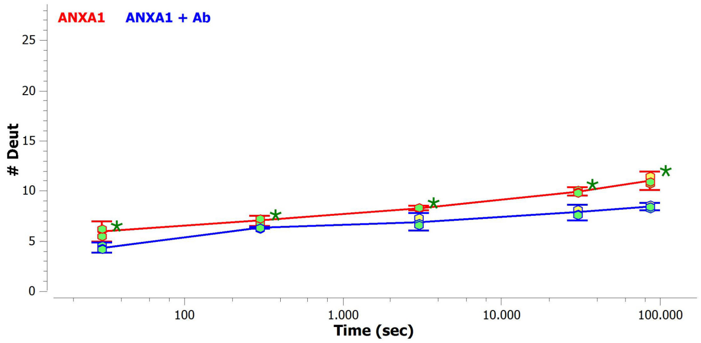


227-257: TTRSYPQLRRVFQKYTKYSKHDMNKVLDLEL (#136)

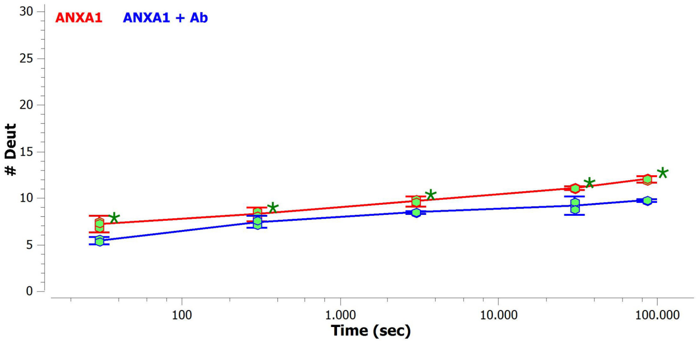


227-267: TTRSYPQLRRVFQKYTKYSKHDMNKVLDLELKGDIEKCLTA (#137)

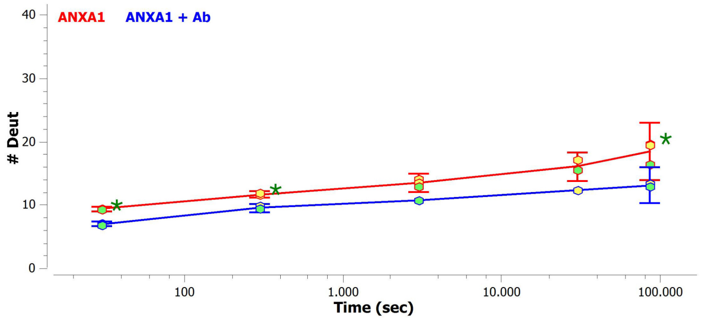


229-234: RSYPQL (#138)

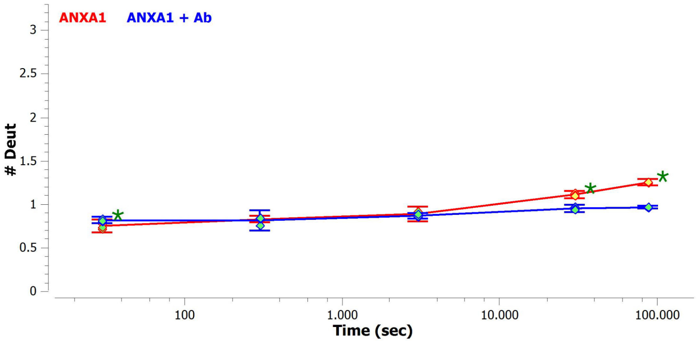


235-254: RRVFQKYTKYSKHDMNKVLD (#139)

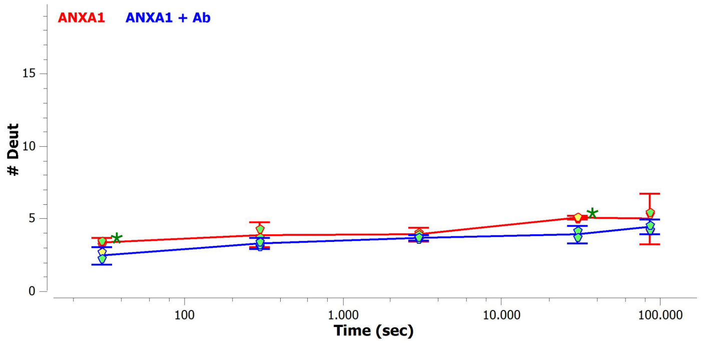


235-255: RRVFQKYTKYSKHDMNKVLDL (#140)

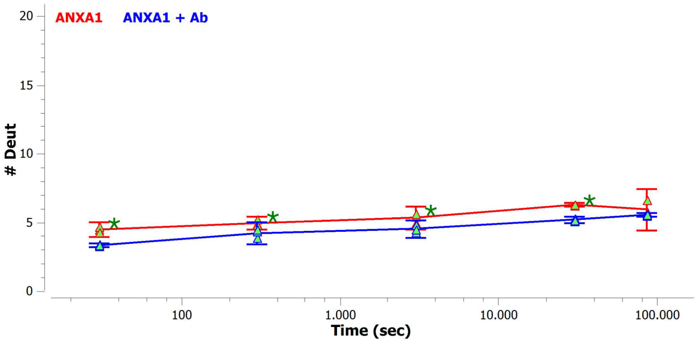

**235-257: RRVFQKYTKYSKHDMNKVLDLEL (#141)**

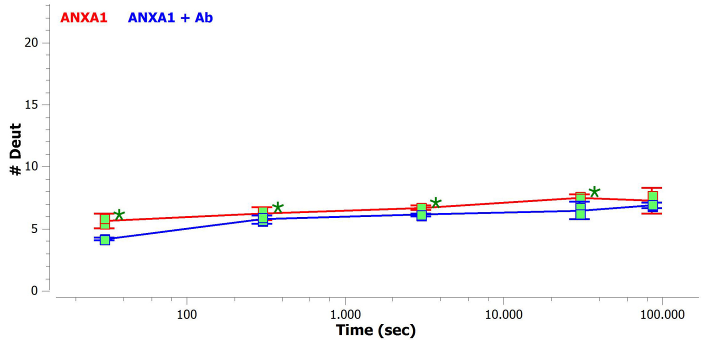


**235-278: RRVFQKYTKYSKHDMNKVLDLELKGDIEKCLTAIVKCATSKPAF (#142)**

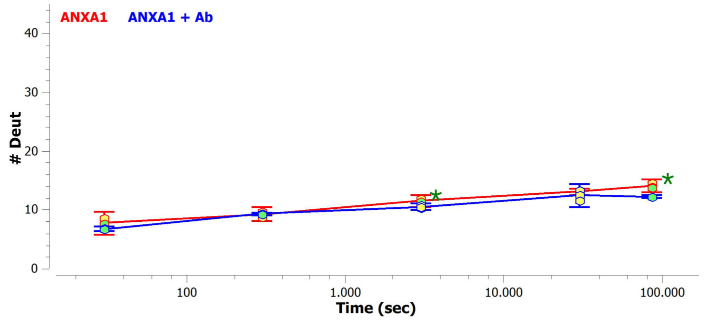


**255-266: LELKGDIEKCLT (#143)**

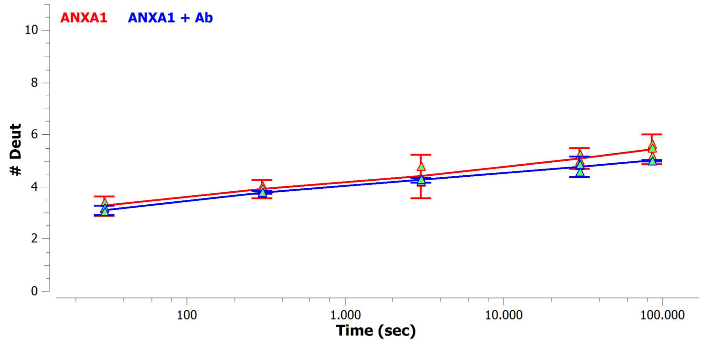


**255-267: LELKGDIEKCLTA (#144)**

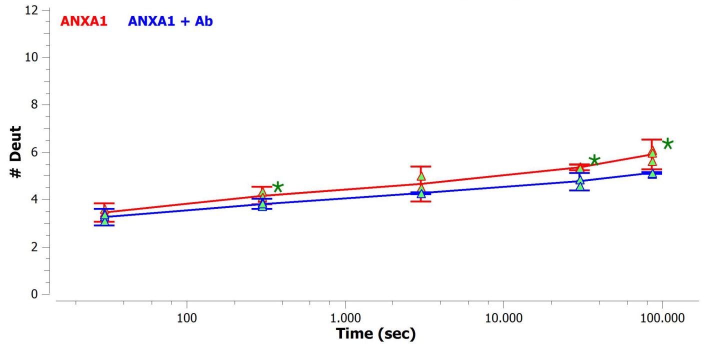


**255-278: LELKGDIEKCLTAIVKCATSKPAF (#145)**

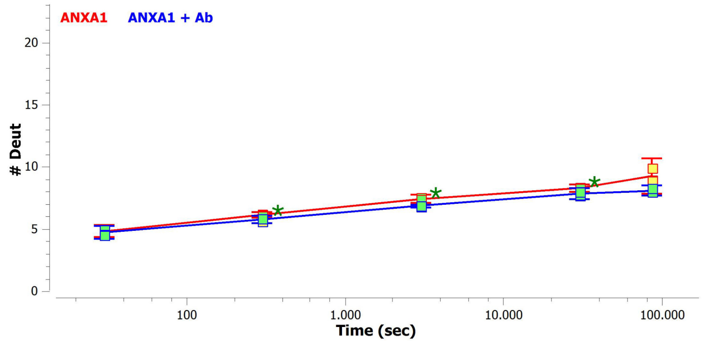


**256-266: ELKGDIEKCLT (#146)**

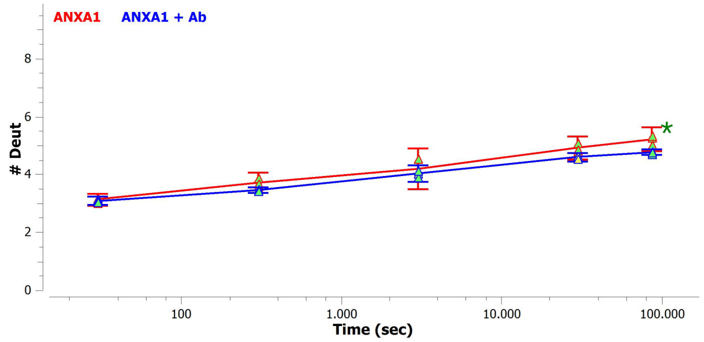


**256-267: ELKGDIEKCLTA (#147)**

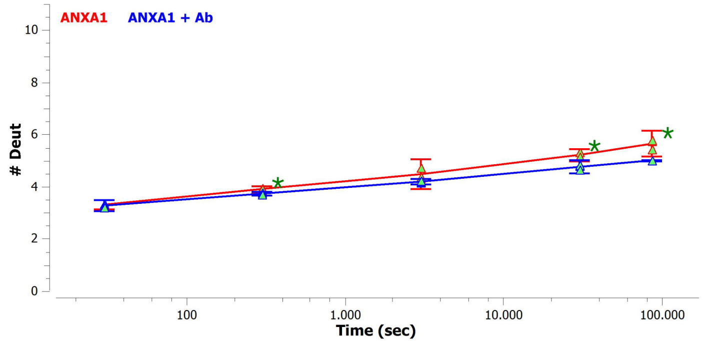


**256-278: ELKGDIEKCLTAIVKCATSKPAF (#148)**

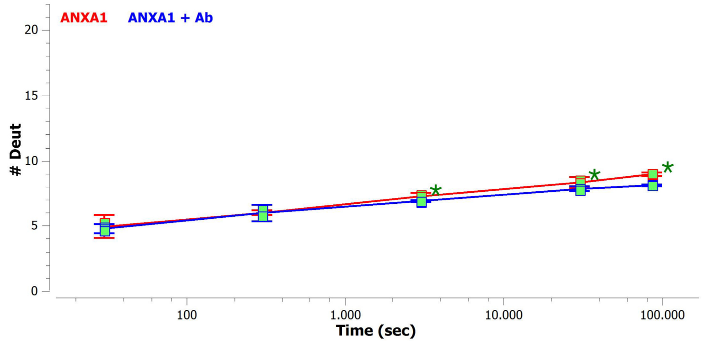


**258-267: KGDIEKCLTA (#149)**

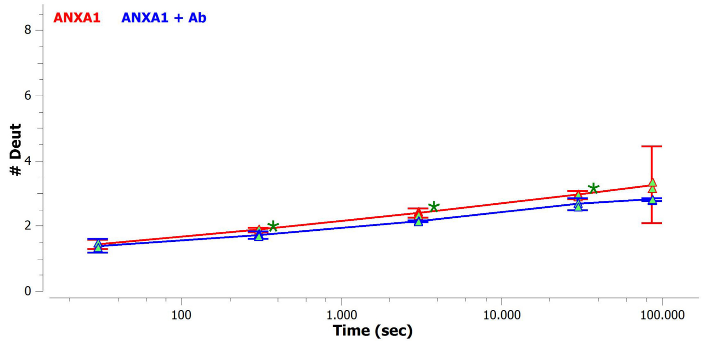


**258-278: KGDIEKCLTAIVKCATSKPAF (#150)**

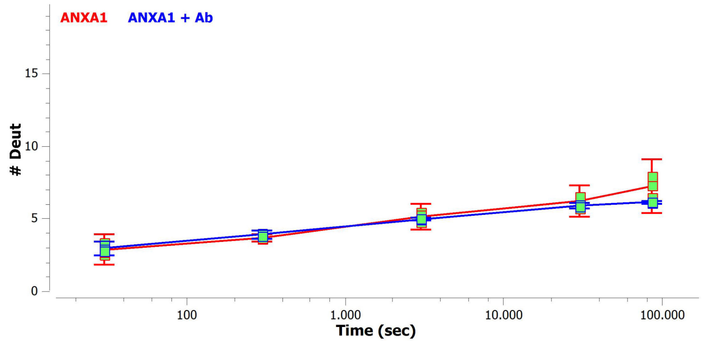

267-278: AIVKCATSKPAF (#151)

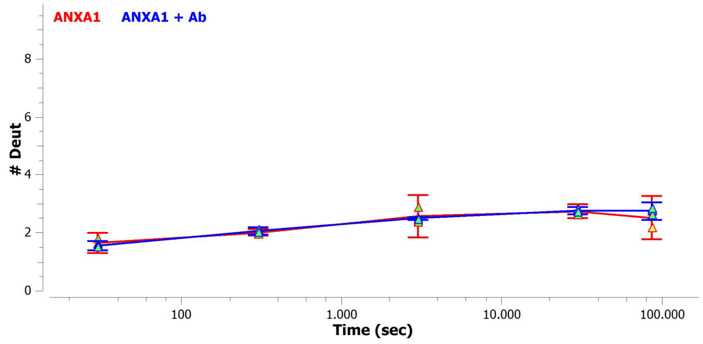


267-279: AIVKCATSKPAFF (#152)

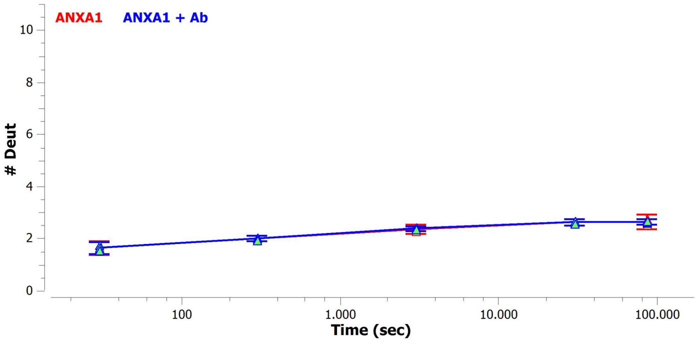


268-279: IVKCATSKPAFF (#153)

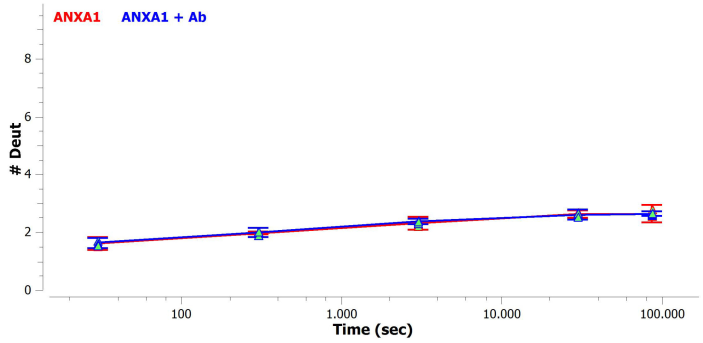


269-278: VKCATSKPAF (#154)

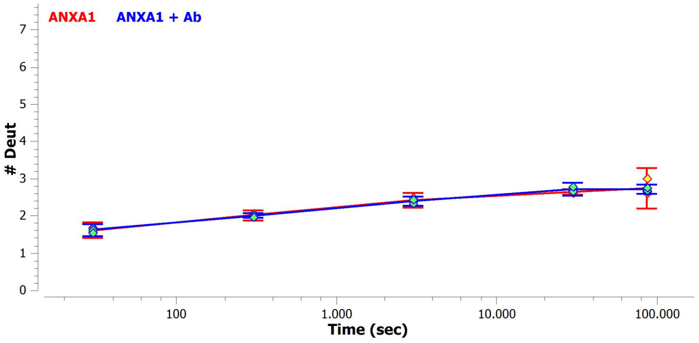


270-278: KCATSKPAF (#155)

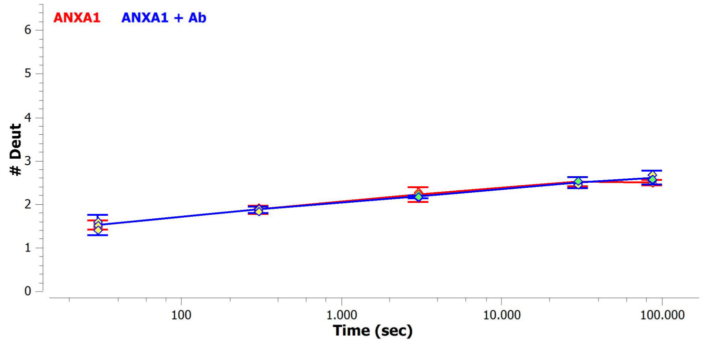


279-297: FAEKLHQAMKGVGTRHKAL (#156)

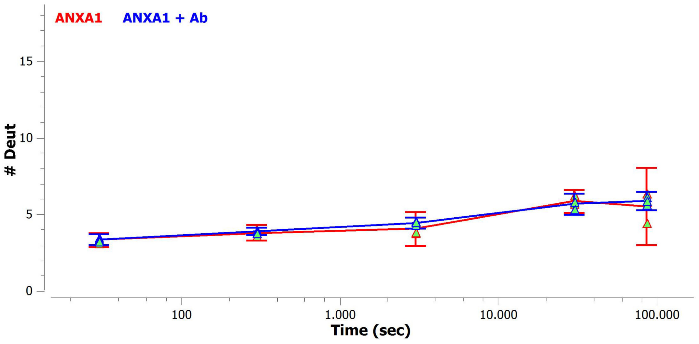


279-300: FAEKLHQAMKGVGTRHKALIRI (#157)

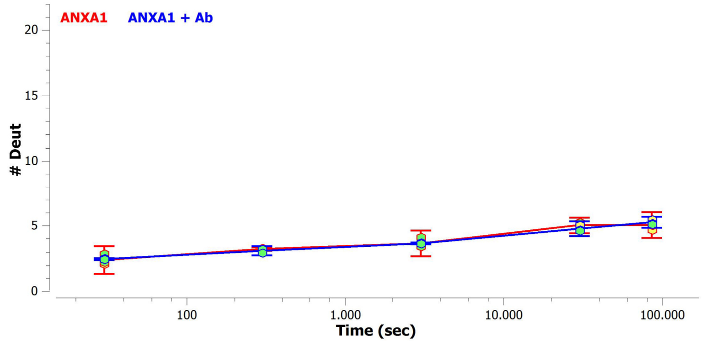


279-301: FAEKLHQAMKGVGTRHKALIRIM (#158)

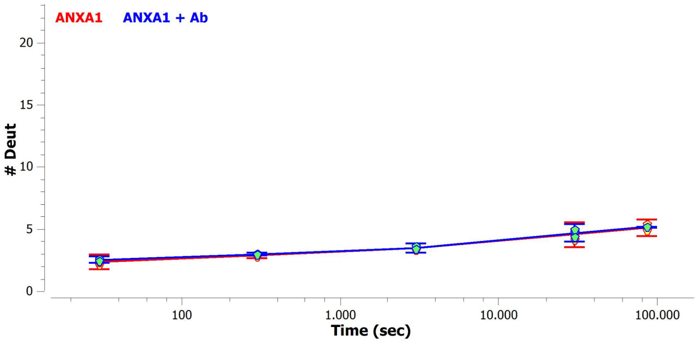


279-309: FAEKLHQAMKGVGTRHKALIRIMVSRSEIDM (#159)

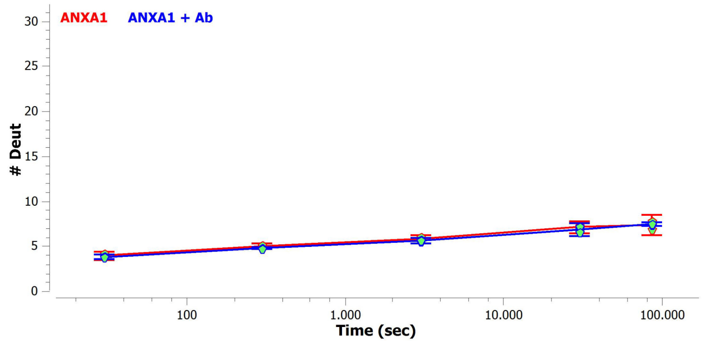


280-309: AEKLHQAMKGVGTRHKALIRIMVSRSEIDM (#160)

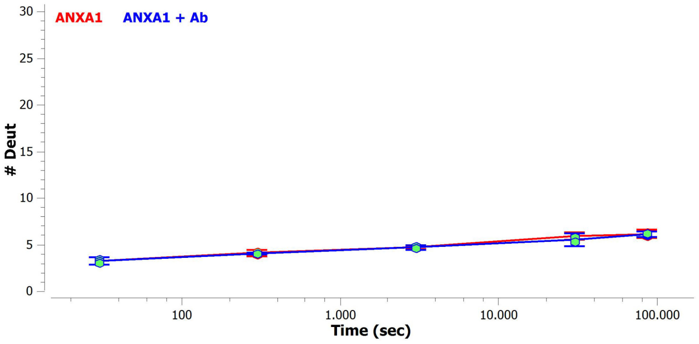

**298-309: IRIMVSRSEIDM (#161)**

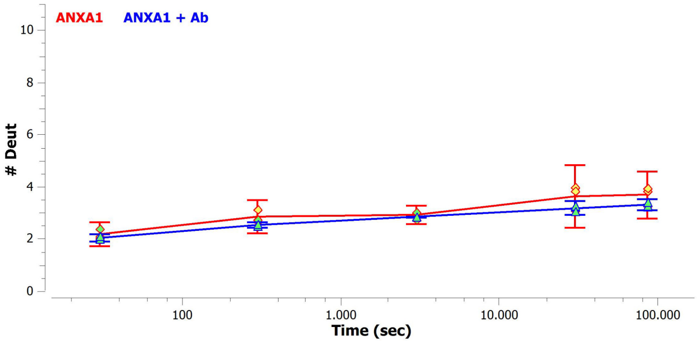


**298-314: IRIMVSRSEIDMNDIKA (#162)**

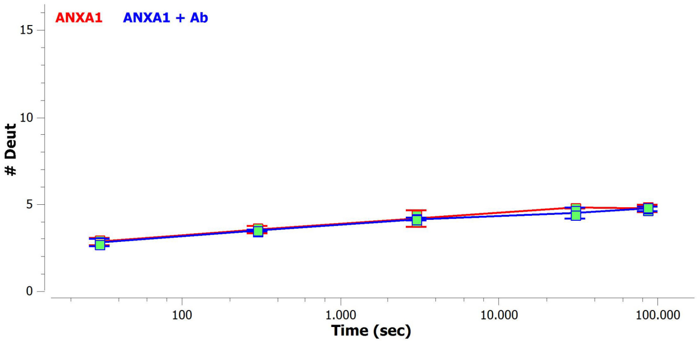


**298-315: IRIMVSRSEIDMNDIKAF (#163)**

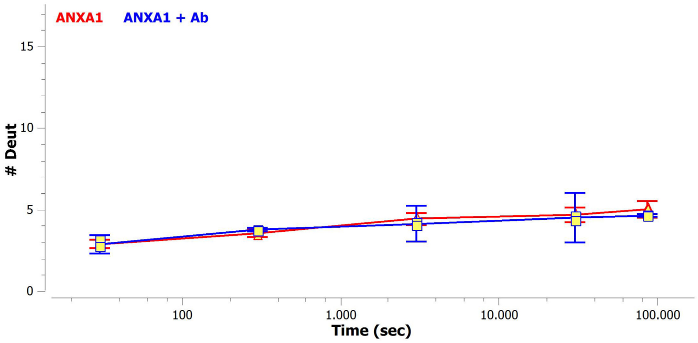


**301-309: MVSRSEIDM (#164)**

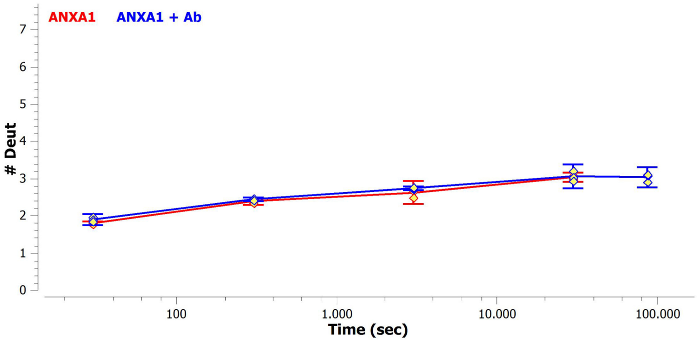


**310-315: NDIKAF (#165)**

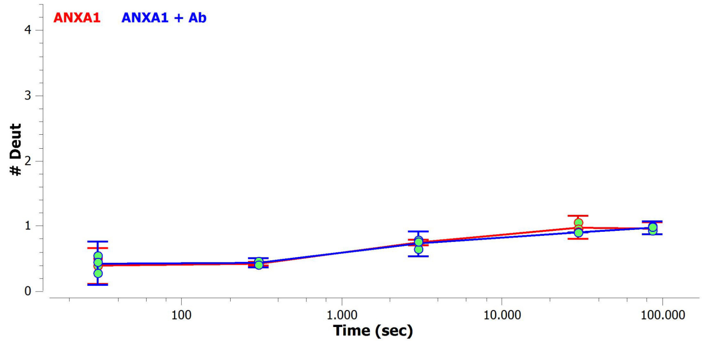


**310-319: NDIKAFYQKM (#166)**

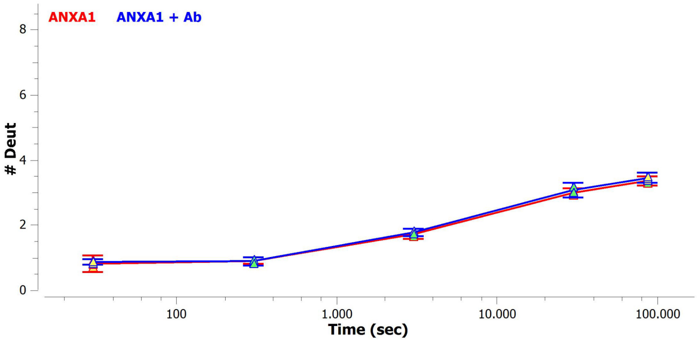


**310-321: NDIKAFYQKMYG (#167)**

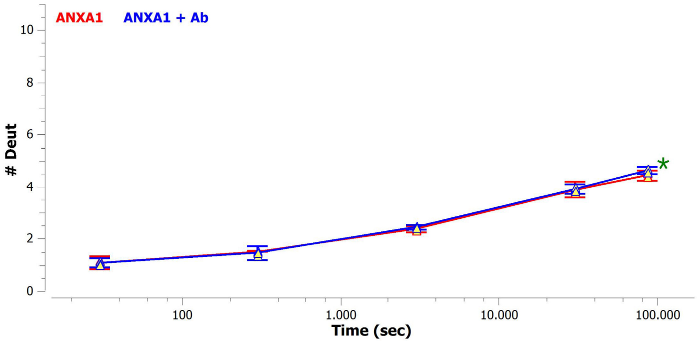


**310-323: NDIKAFYQKMYGIS (#168)**

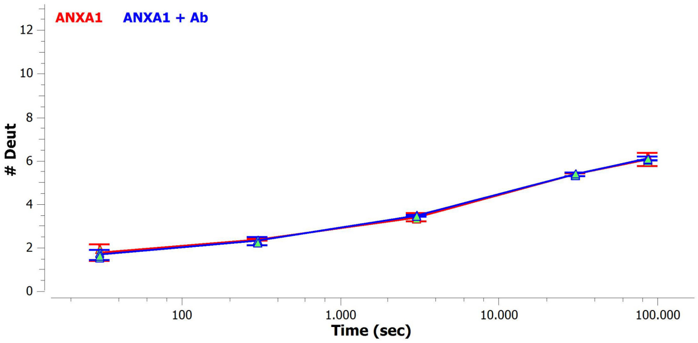


**310-324: NDIKAFYQKMYGISL (#169)**

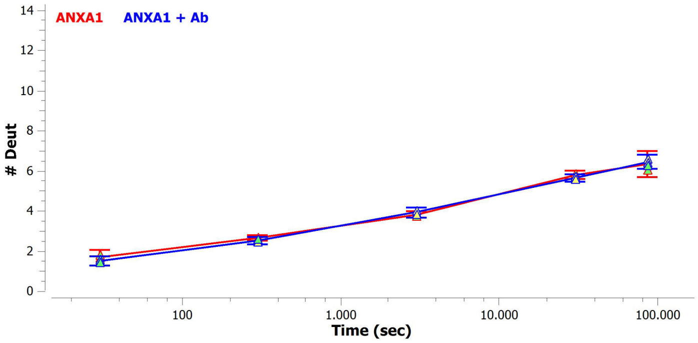


**315-323: FYQKMYGIS (#170)**

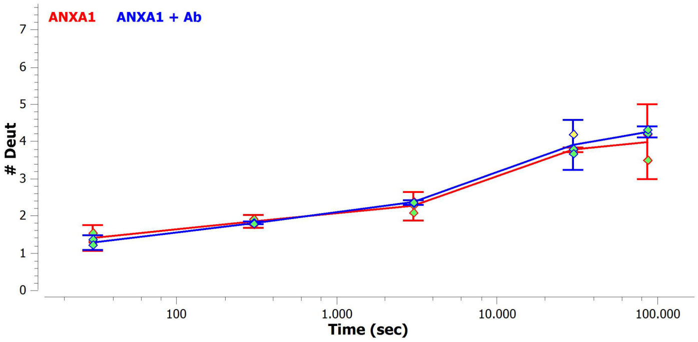

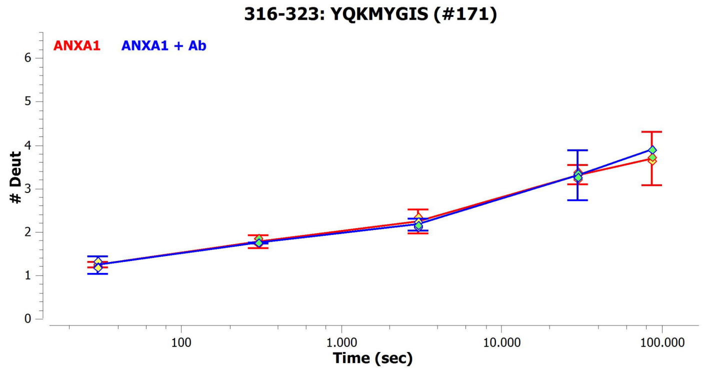
316-323: YQKMYGIS (#171)
ANXA1
ANXA1 + Ab
# Deut
Time (sec)

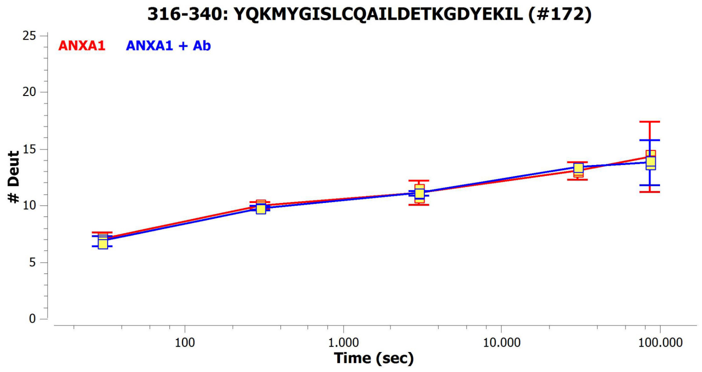
316-340: YQKMYGISLCQAILDETKGDYEKIL (#172)
ANXA1
ANXA1 + Ab
# Deut
Time (sec)

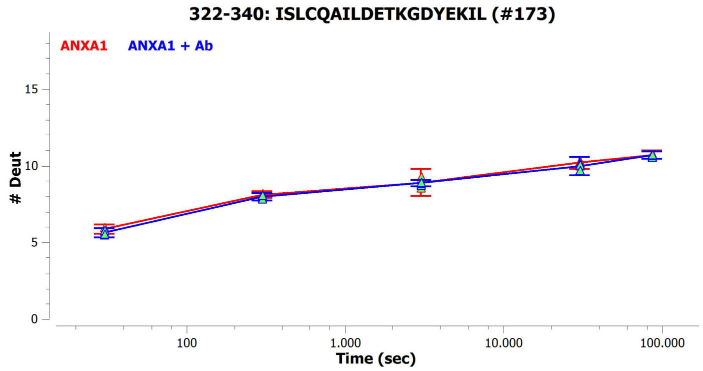
322-340: ISLCQAILDETKGDYEKIL (#173)
ANXA1
ANXA1 + Ab
# Deut
Time (sec)

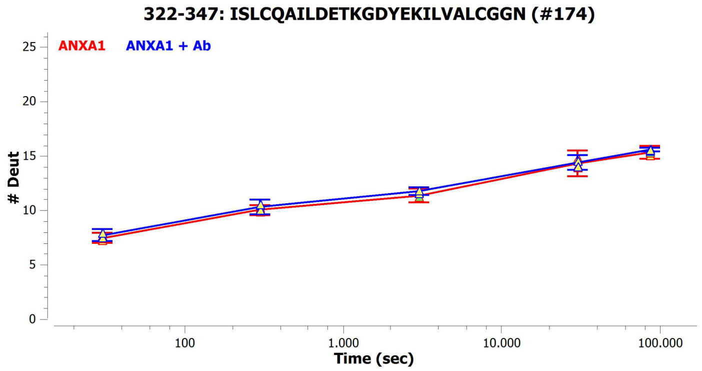
322-347: ISLCQAILDETKGDYEKILVALCGGN (#174)
ANXA1
ANXA1 + Ab
# Deut
Time (sec)

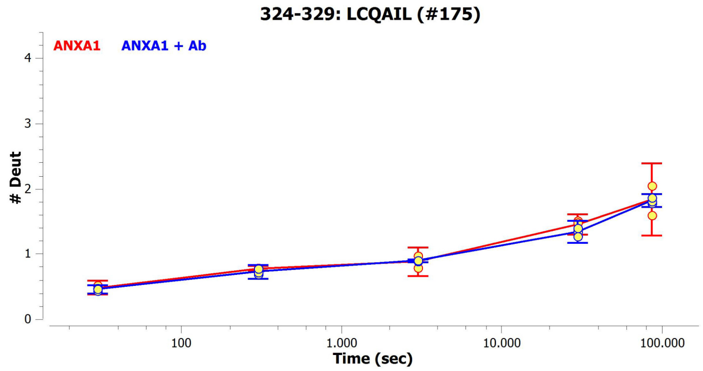
324-329: LCQAIL (#175)
ANXA1
ANXA1 + Ab
# Deut
Time (sec)

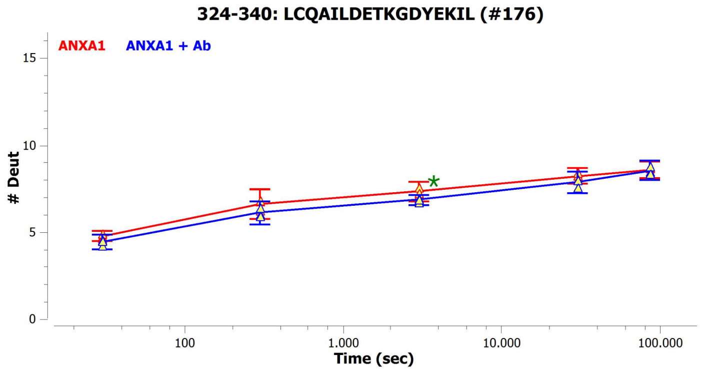
324-340: LCQAILDETKGDYEKIL (#176)
ANXA1
ANXA1 + Ab
# Deut
Time (sec)

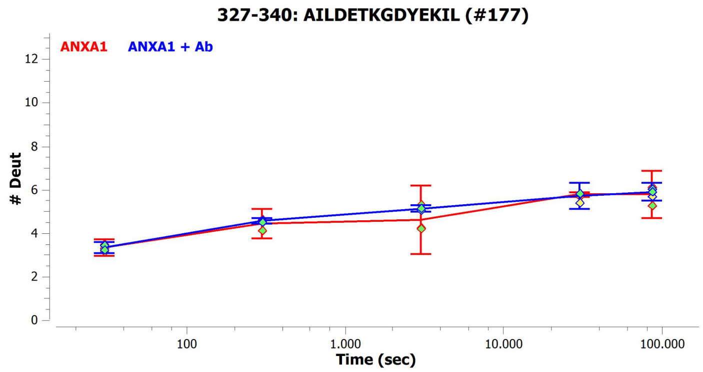
327-340: AILDETKGDYEKIL (#177)
ANXA1
ANXA1 + Ab
# Deut
Time (sec)

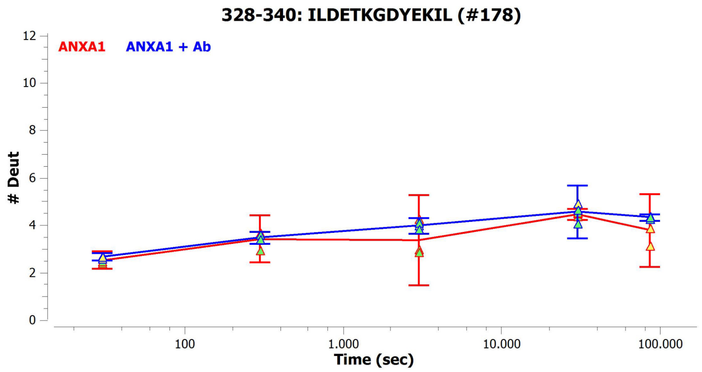
328-340: ILDETKGDYEKIL (#178)
ANXA1
ANXA1 + Ab
# Deut
Time (sec)

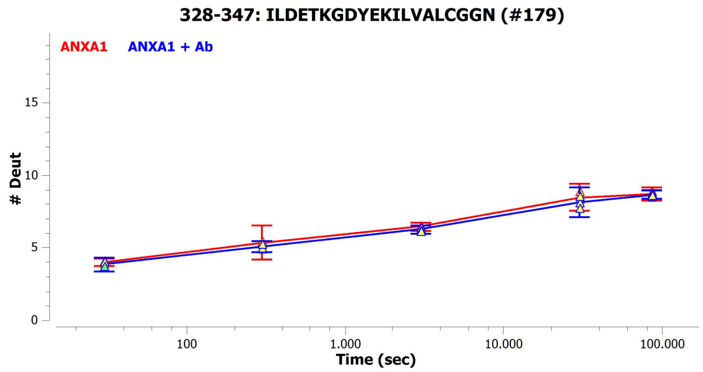
328-347: ILDETKGDYEKILVALCGGN (#179)
ANXA1
ANXA1 + Ab
# Deut
Time (sec)

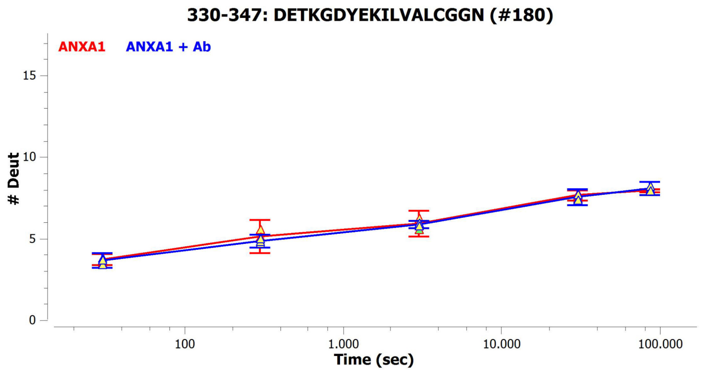
330-347: DETKGDYEKILVALCGGN (#180)
ANXA1
ANXA1 + Ab
# Deut
Time (sec)

Figure S6: All peptide uptake plots of the **in-solution digest**. used in this publication of the unbound state shown in red and the bound state shown in blue. Time points marked with a star show significant differences ($p<0.05$) between both states determined by students t-test. The Y-Axis scaled to 100% possible exchange of each peptide and the error bars are based on the 95% confidence level.

1-7: GPAMVSE (#1)

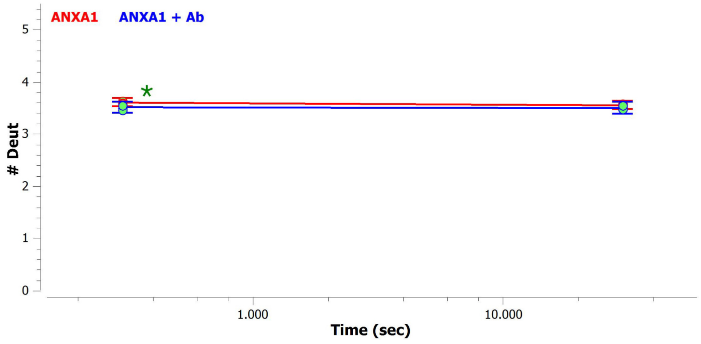


1-13: GPAMVSEFLKQAW (#2)

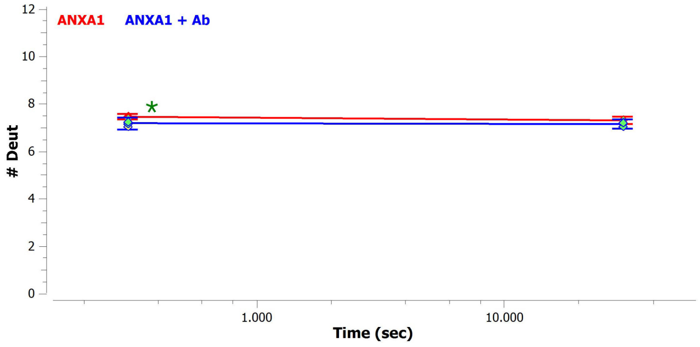


1-21: GPAMVSEFLKQAWFIENEEQE (#3)

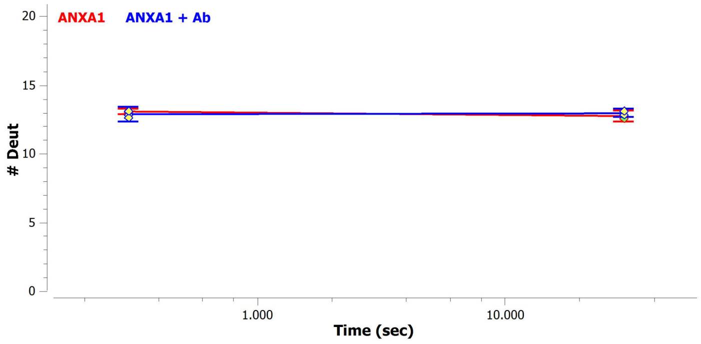


5-8: VSEF (#4)

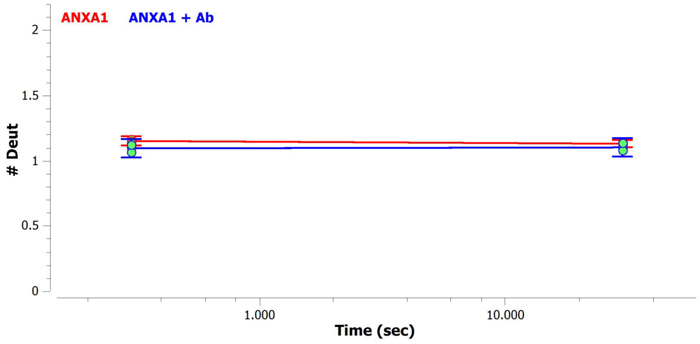


8-13: FLKQAW (#5)

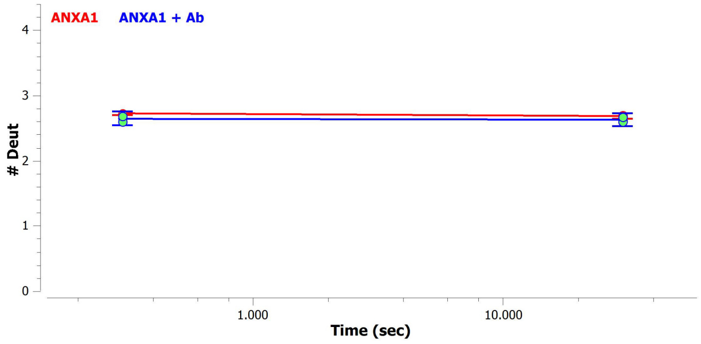


8-14: FLKQAWF (#6)

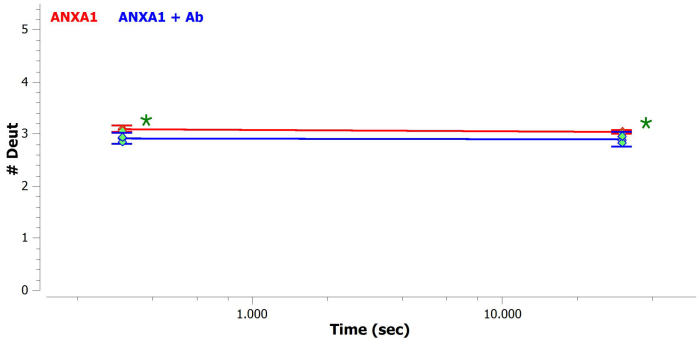


9-13: LKQAW (#7)

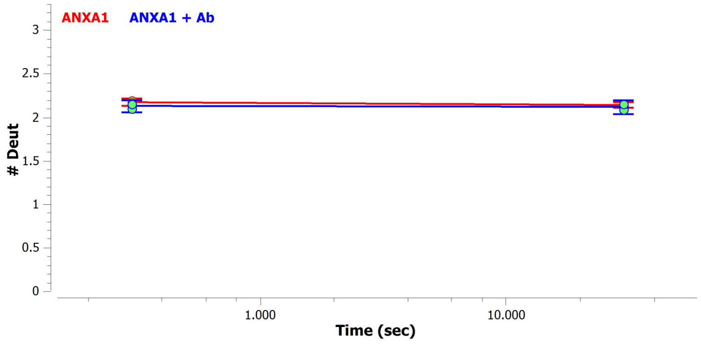


9-14: LKQAWF (#8)

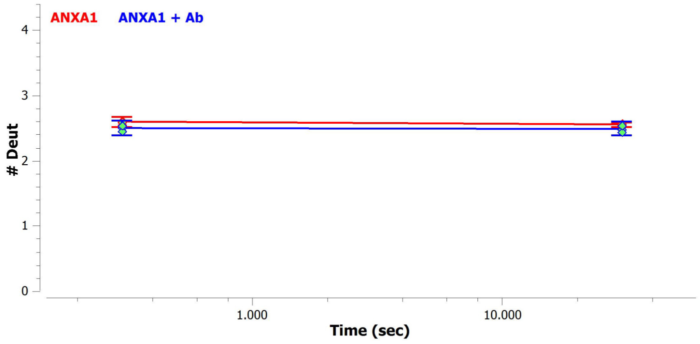


14-18: FIENE (#9)

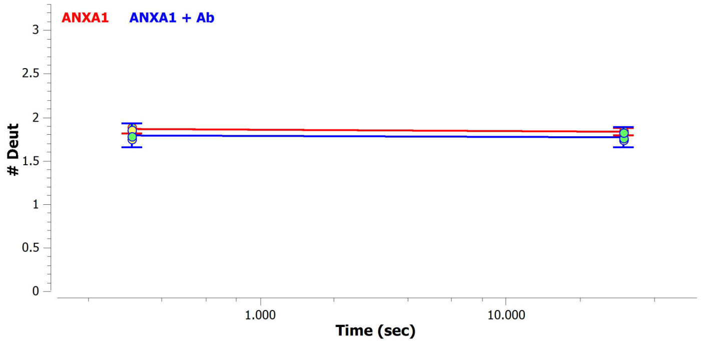


14-21: FIENEEQE (#10)

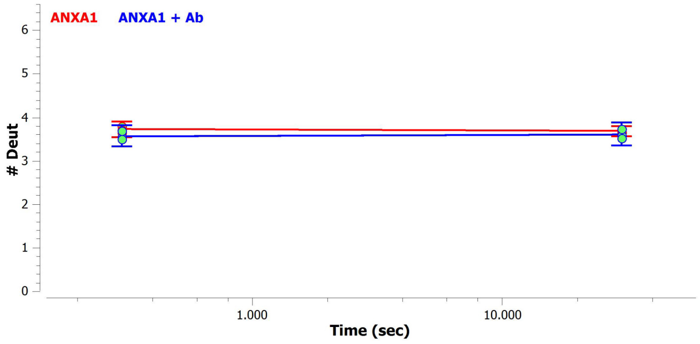

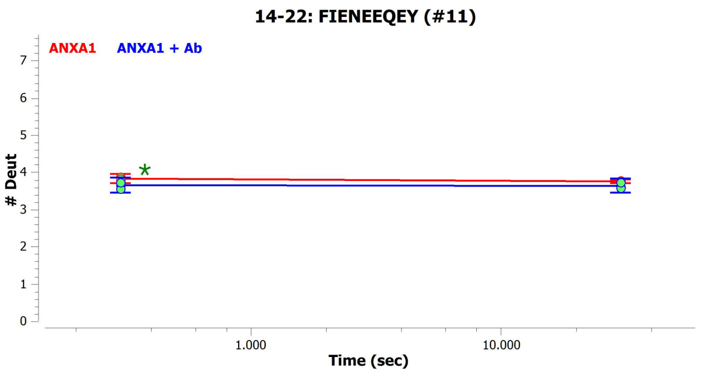
14-22: FIENEEQEY (#11)
ANXA1
ANXA1 + Ab
# Deut
Time (sec)
1.000
10.000

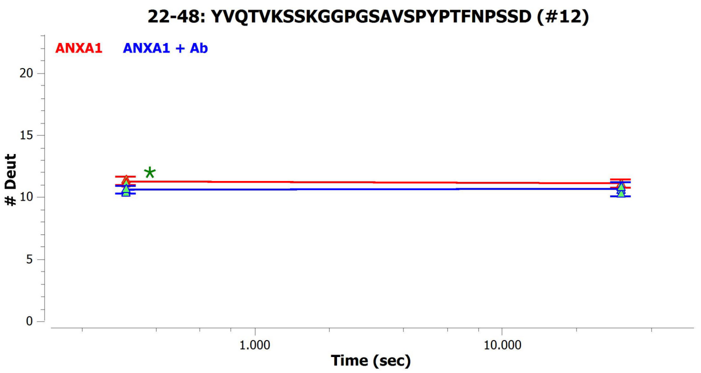
22-48: YVQTVKSSKGGPGSAVSPYPTFNPSSD (#12)
ANXA1
ANXA1 + Ab
# Deut
Time (sec)
1.000
10.000

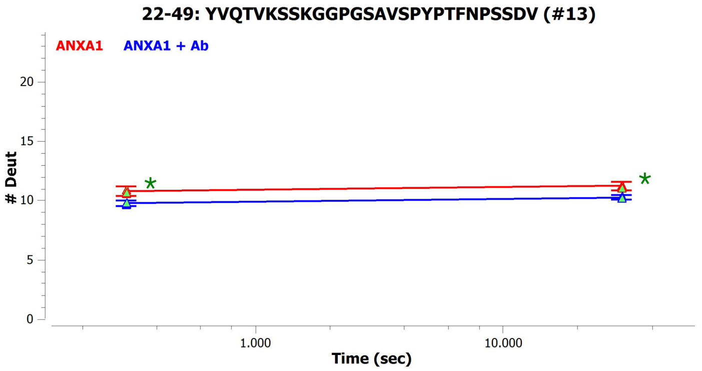
22-49: YVQTVKSSKGGPGSAVSPYPTFNPSSDV (#13)
ANXA1
ANXA1 + Ab
# Deut
Time (sec)
1.000
10.000

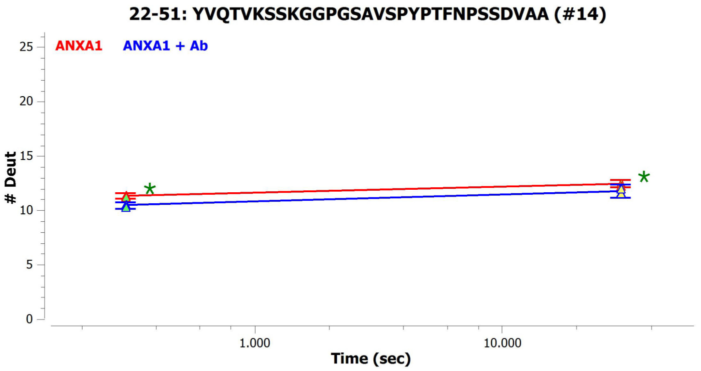
22-51: YVQTVKSSKGGPGSAVSPYPTFNPSSDVAA (#14)
ANXA1
ANXA1 + Ab
# Deut
Time (sec)
1.000
10.000

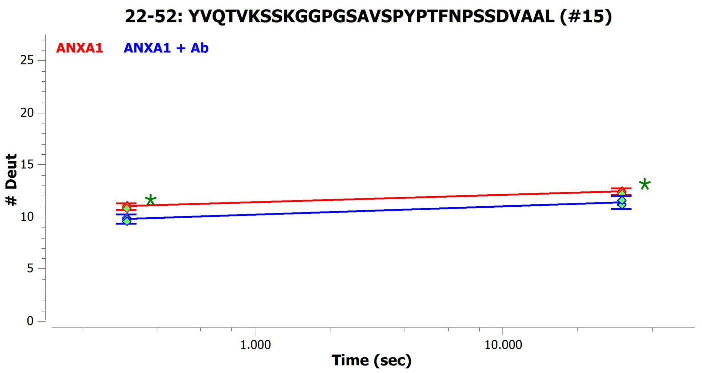
22-52: YVQTVKSSKGGPGSAVSPYPTFNPSSDVAAL (#15)
ANXA1
ANXA1 + Ab
# Deut
Time (sec)
1.000
10.000

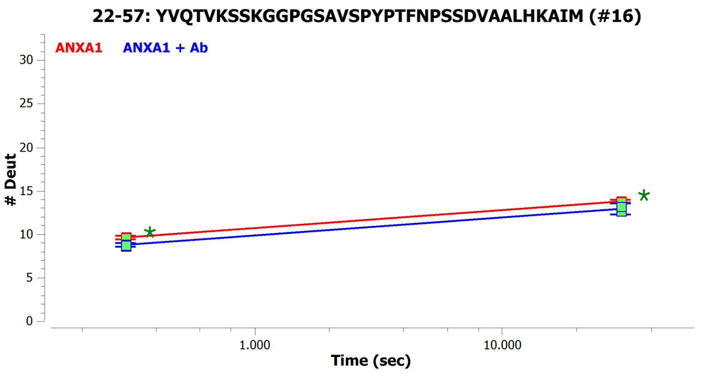
22-57: YVQTVKSSKGGPGSAVSPYPTFNPSSDVAALHKAIM (#16)
ANXA1
ANXA1 + Ab
# Deut
Time (sec)
1.000
10.000

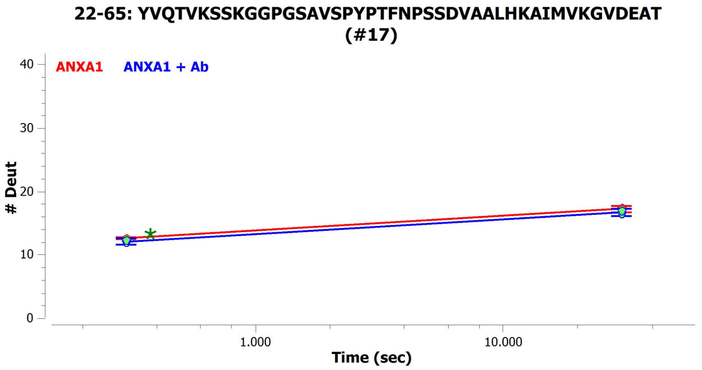
22-65: YVQTVKSSKGGPGSAVSPYPTFNPSSDVAALHKAIMVKGVDEAT (#17)
ANXA1
ANXA1 + Ab
# Deut
Time (sec)
1.000
10.000

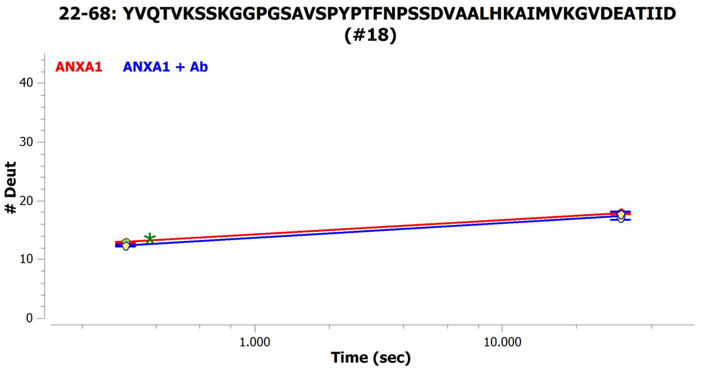
22-68: YVQTVKSSKGGPGSAVSPYPTFNPSSDVAALHKAIMVKGVDEATIID (#18)
ANXA1
ANXA1 + Ab
# Deut
Time (sec)
1.000
10.000

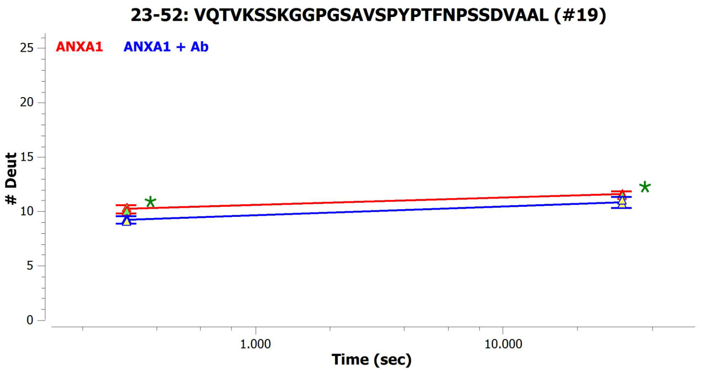
23-52: VQTVKSSKGGPGSAVSPYPTFNPSSDVAAL (#19)
ANXA1
ANXA1 + Ab
# Deut
Time (sec)
1.000
10.000

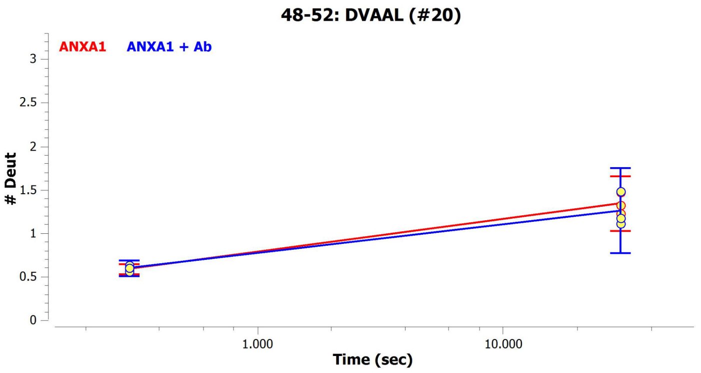
48-52: DVAAL (#20)
ANXA1
ANXA1 + Ab
# Deut
Time (sec)
1.000
10.000

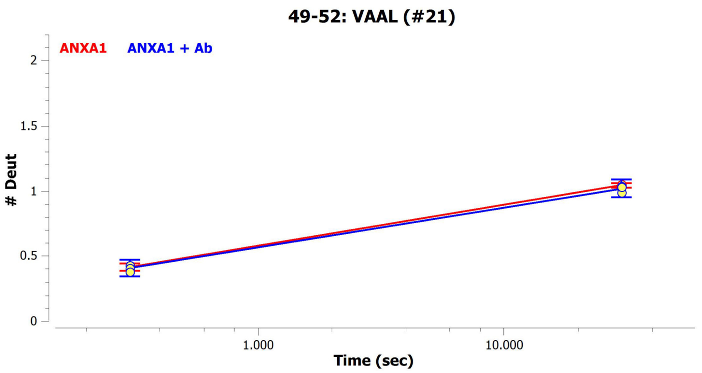
49-52: VAAL (#21)
ANXA1
ANXA1 + Ab
# Deut
0
0.5
1
1.5
2
1.000
10.000
Time (sec)

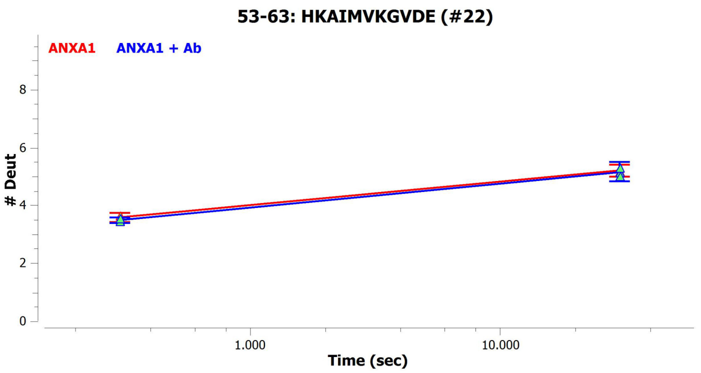
53-63: HKAIMVKGVDE (#22)
ANXA1
ANXA1 + Ab
# Deut
0
2
4
6
8
1.000
10.000
Time (sec)

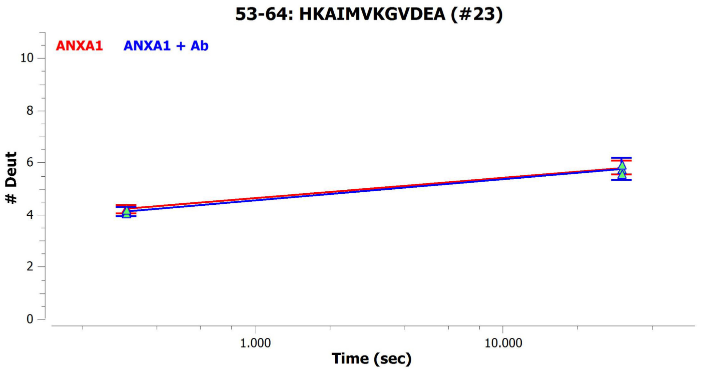
53-64: HKAIMVKGVDEA (#23)
ANXA1
ANXA1 + Ab
# Deut
0
2
4
6
8
10
1.000
10.000
Time (sec)

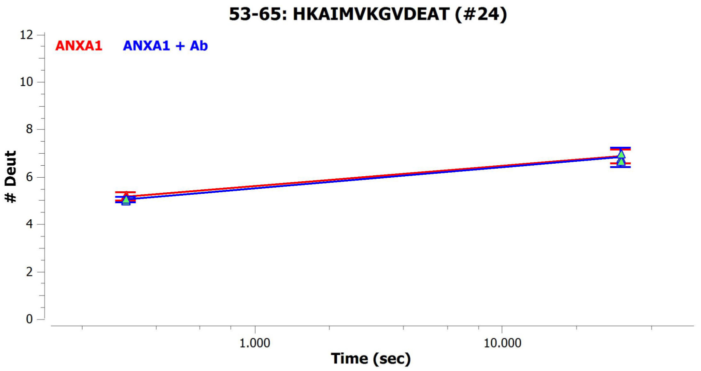
53-65: HKAIMVKGVDEAT (#24)
ANXA1
ANXA1 + Ab
# Deut
0
2
4
6
8
10
12
1.000
10.000
Time (sec)

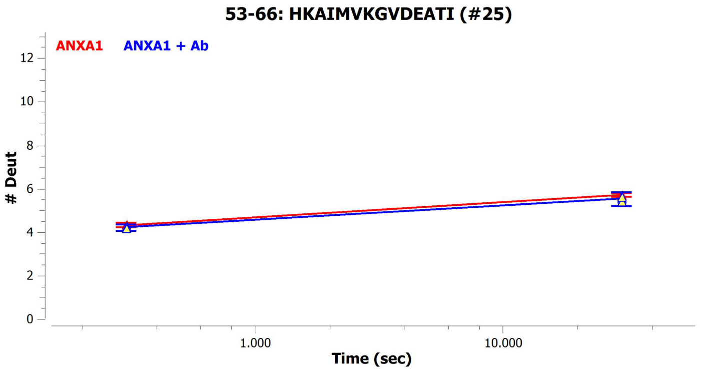
53-66: HKAIMVKGVDEATI (#25)
ANXA1
ANXA1 + Ab
# Deut
0
2
4
6
8
10
12
1.000
10.000
Time (sec)

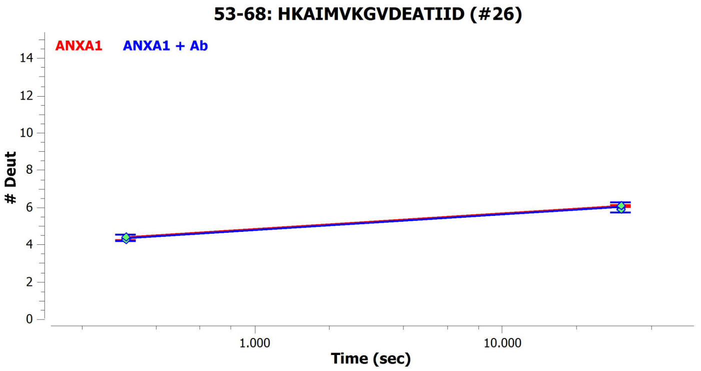
53-68: HKAIMVKGVDEATIID (#26)
ANXA1
ANXA1 + Ab
# Deut
0
2
4
6
8
10
12
14
1.000
10.000
Time (sec)

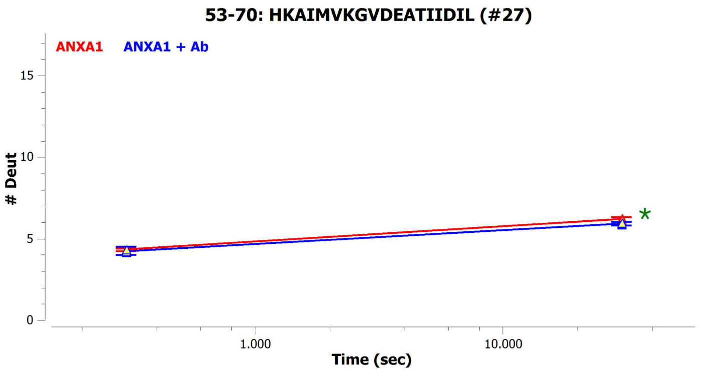
53-70: HKAIMVKGVDEATIIDIL (#27)
ANXA1
ANXA1 + Ab
# Deut
0
5
10
15
*
1.000
10.000
Time (sec)

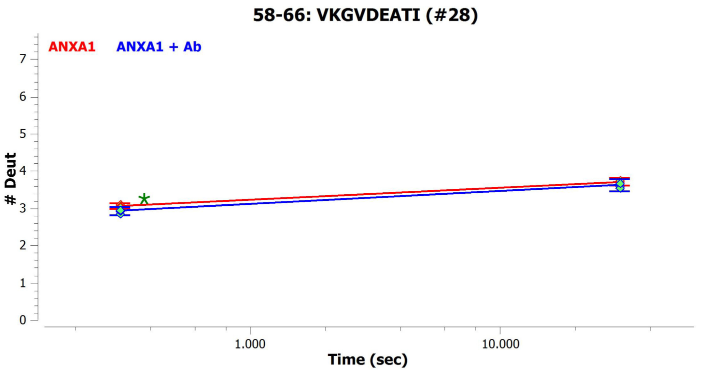
58-66: VKGVDEATI (#28)
ANXA1
ANXA1 + Ab
# Deut
0
1
2
3
4
5
6
7
*
1.000
10.000
Time (sec)

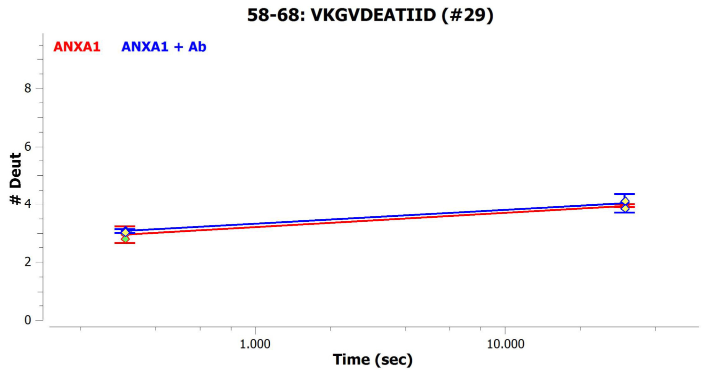
58-68: VKGVDEATIID (#29)
ANXA1
ANXA1 + Ab
# Deut
0
2
4
6
8
1.000
10.000
Time (sec)

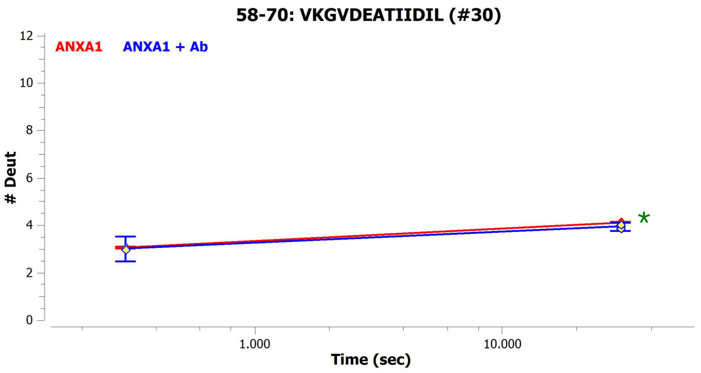
58-70: VKGVDEATIIDIL (#30)
ANXA1
ANXA1 + Ab
# Deut
0
2
4
6
8
10
12
*
1.000
10.000
Time (sec)

58-85: VKGVDEATIIDILTKRNNAQRQQIKAAY (#31)

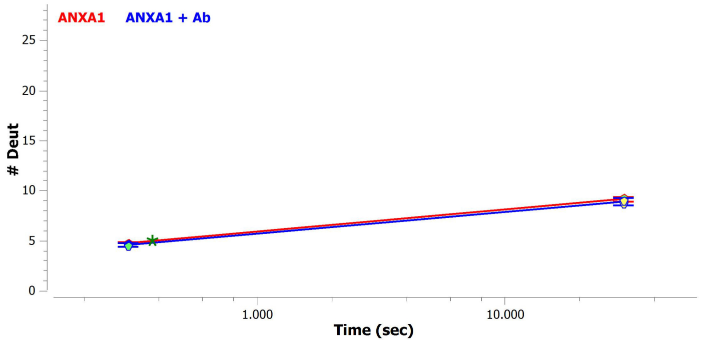


63-68: EATIID (#32)

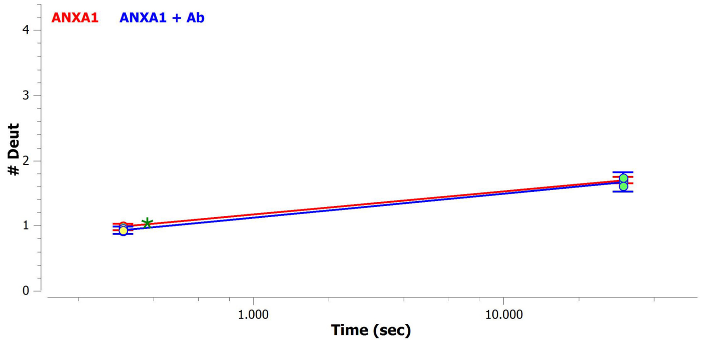


63-70: EATIIDIL (#33)

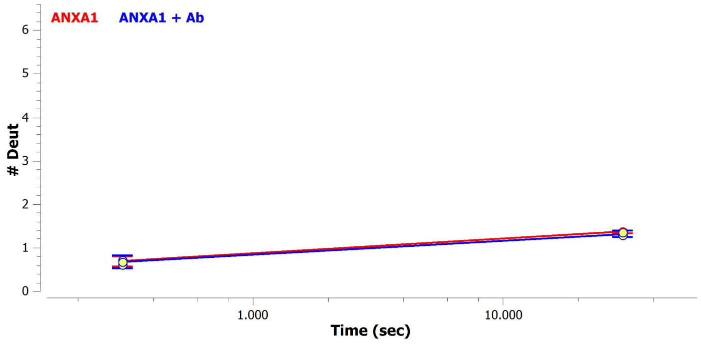


64-68: ATIID (#34)

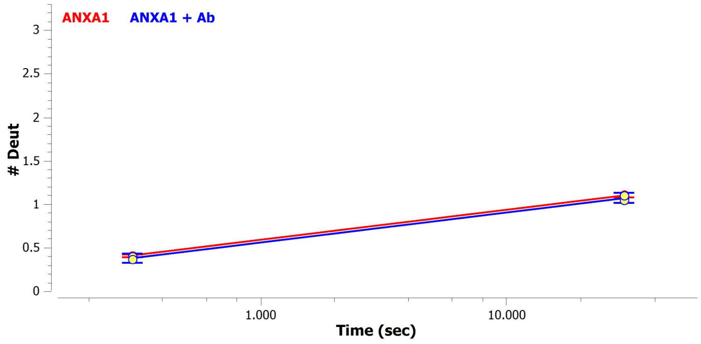


64-70: ATIIDIL (#35)

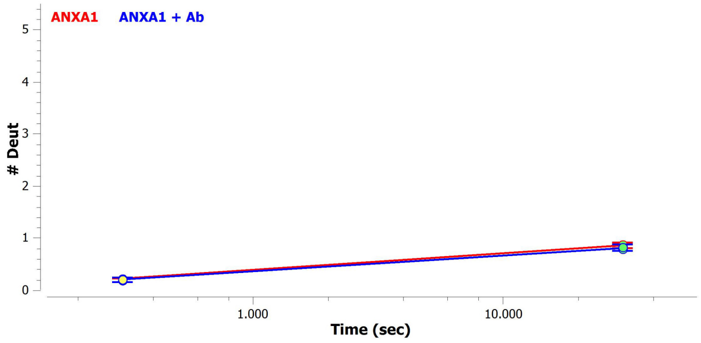


64-85: ATIIDILTKRNNAQRQQIKAAY (#36)

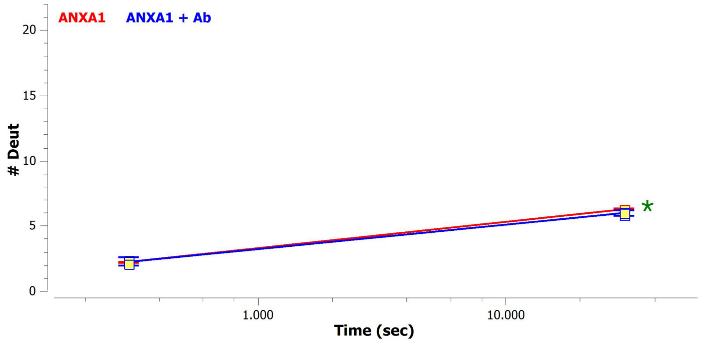


65-70: TIIDIL (#37)

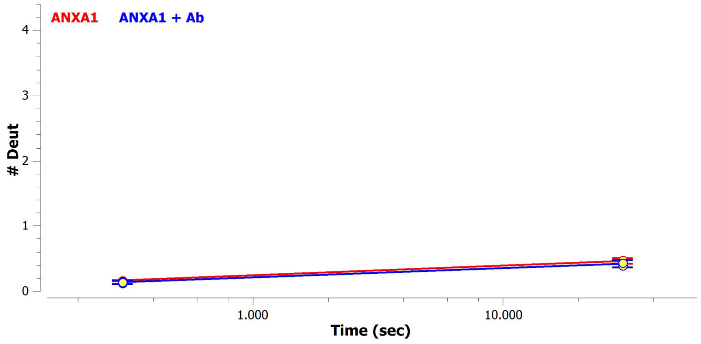


66-70: IIDIL (#38)

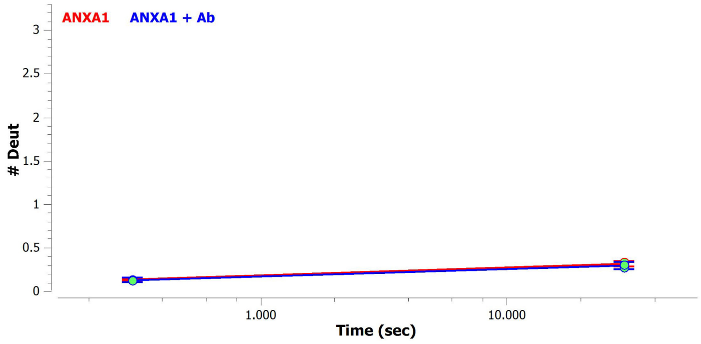


66-85: IIDILTKRNNAQRQQIKAAY (#39)

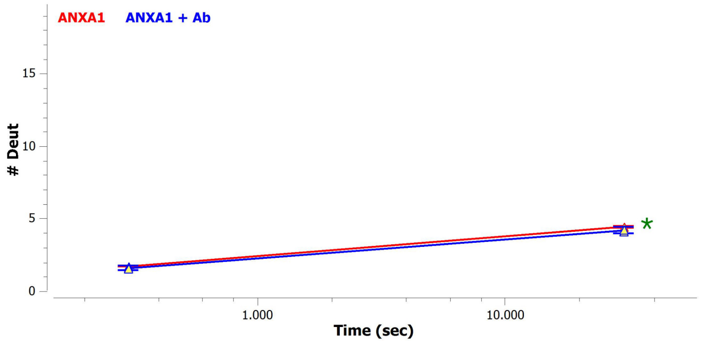


67-70: IDIL (#40)

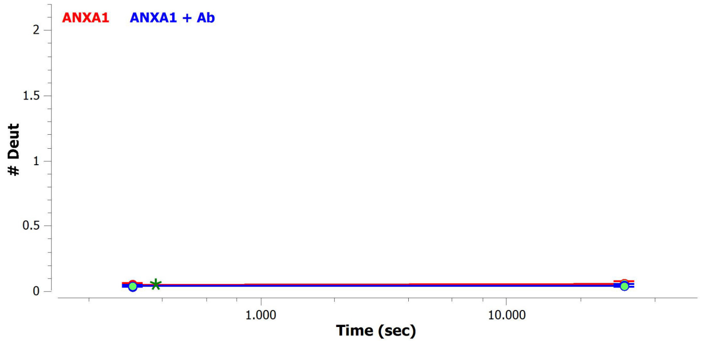

**67-83: IDILTKRNNAQRQQIKA (#41)**

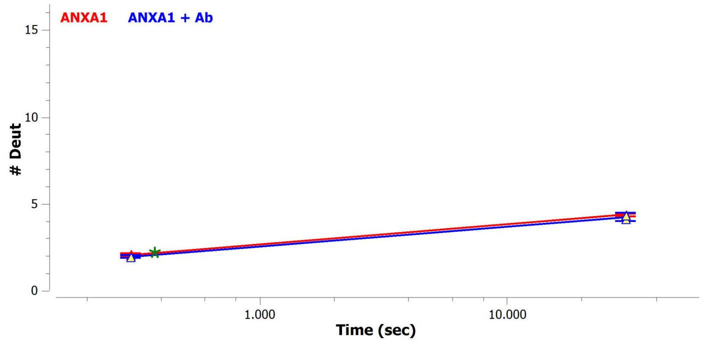


**67-85: IDILTKRNNAQRQQIKAAY (#42)**

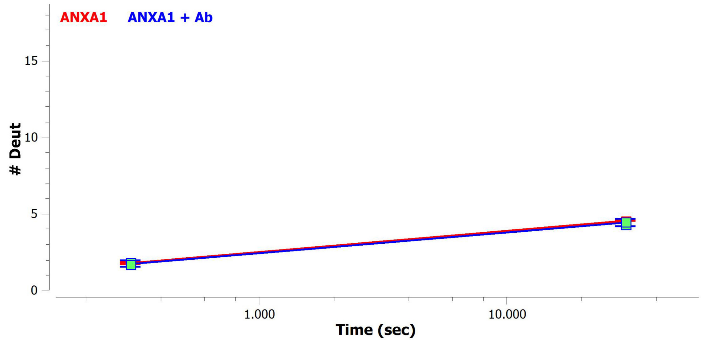


**68-86: DILTKRNNAQRQQIKAAYL (#43)**

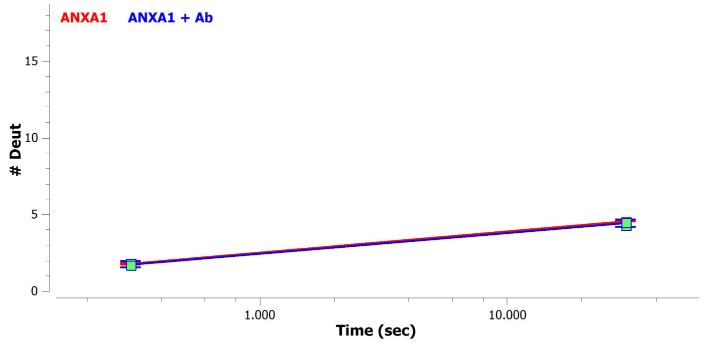


**69-85: ILTKRNNAQRQQIKAAY (#44)**

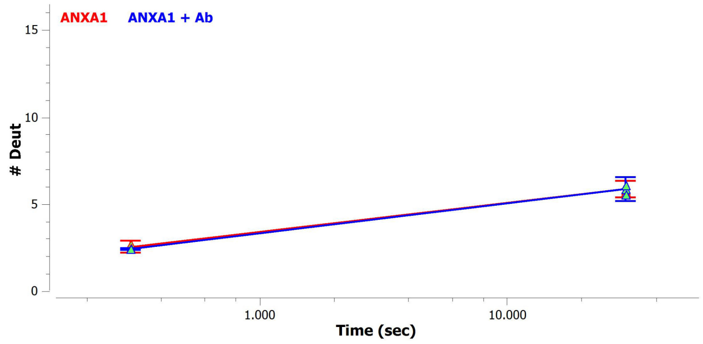


**69-86: ILTKRNNAQRQQIKAAYL (#45)**

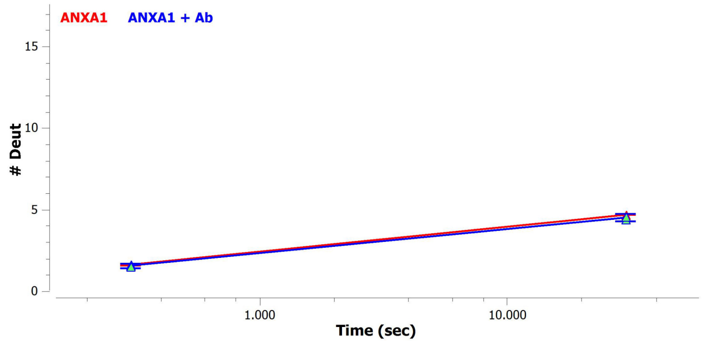


**69-107: ILTKRNNAQRQQIKAAYLQETGKPLDETLKKALTGHLEE (#46)**

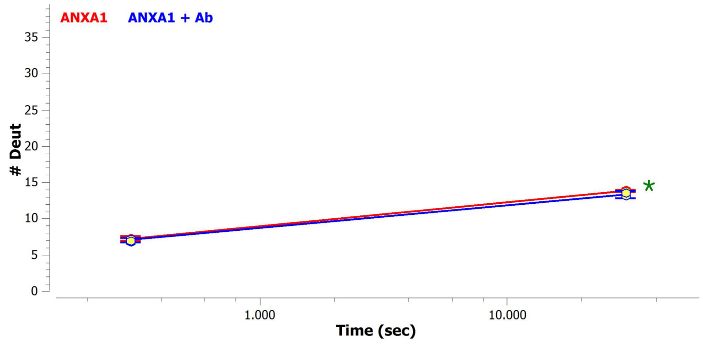


**70-86: LTKRNNAQRQQIKAAYL (#47)**

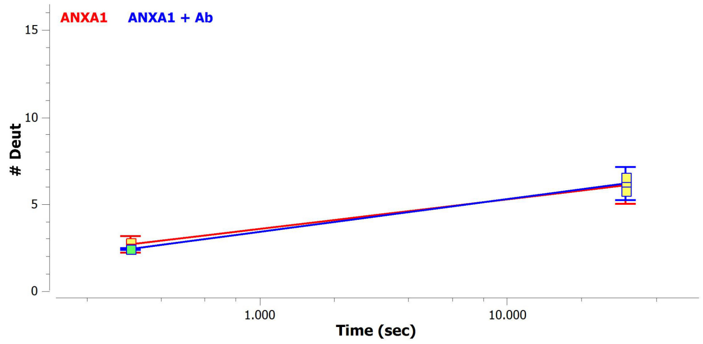


**71-86: TKRNNAQRQQIKAAYL (#48)**

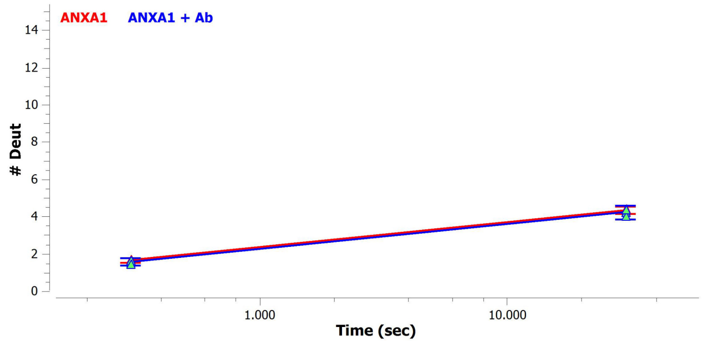


**84-97: AYLQETGKPLDETL (#49)**

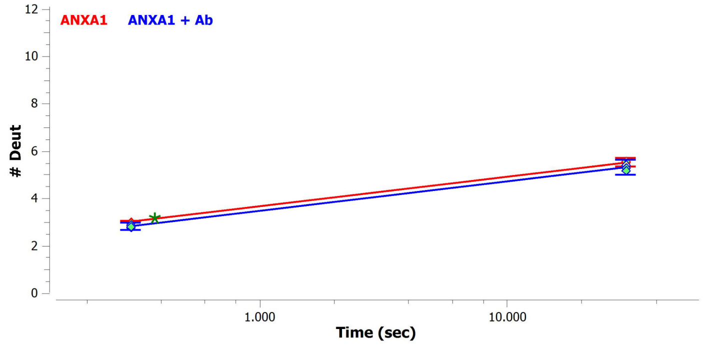


**84-105: AYLQETGKPLDETLKKALTGHL (#50)**

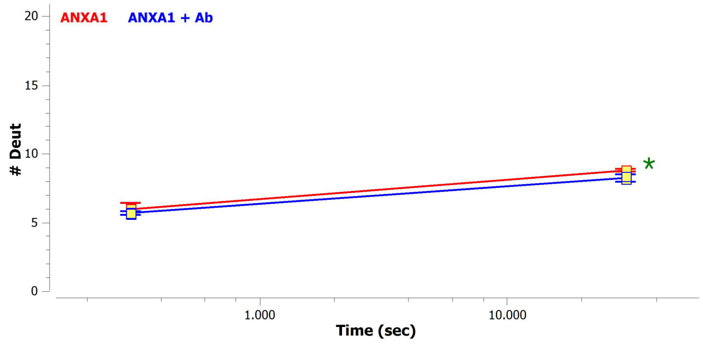

### 84-107: AYLQETGKPLDETLKKALTGHLEE (#51)

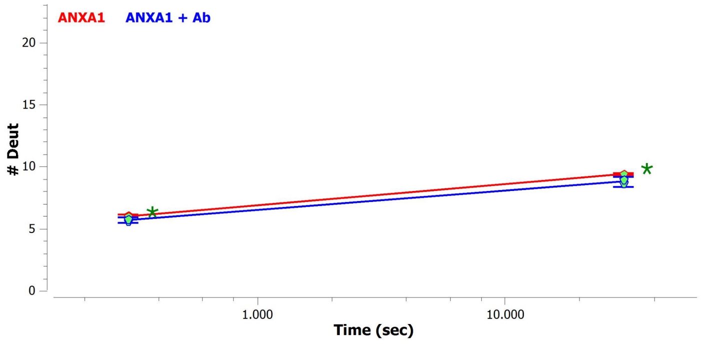


### 86-97: LQETGKPLDETL (#52)

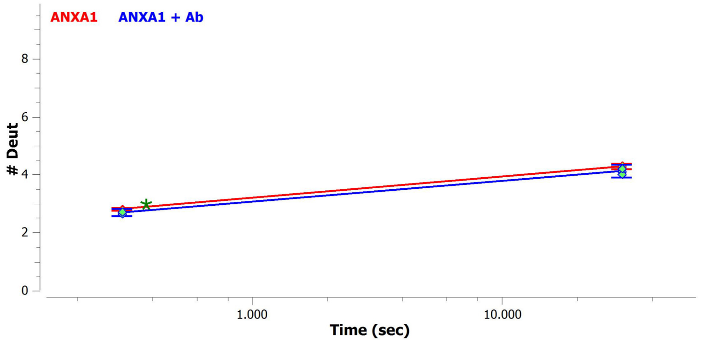


### 86-105: LQETGKPLDETLKKALTGHL (#53)

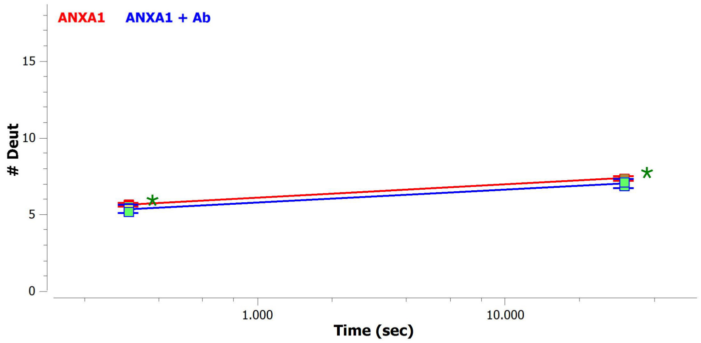


### 86-107: LQETGKPLDETLKKALTGHLEE (#54)

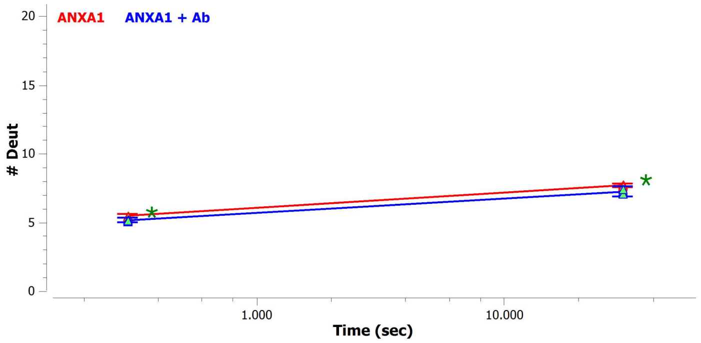


### 87-97: QETGKPLDETL (#55)

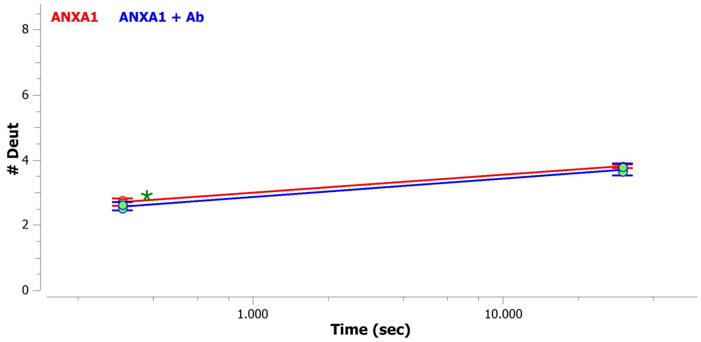


### 87-105: QETGKPLDETLKKALTGHL (#56)

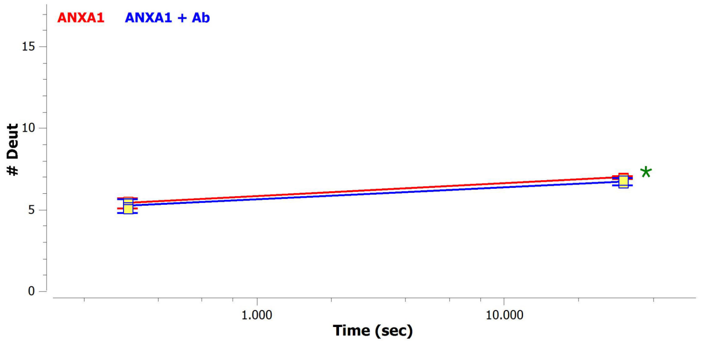


### 87-107: QETGKPLDETLKKALTGHLEE (#57)

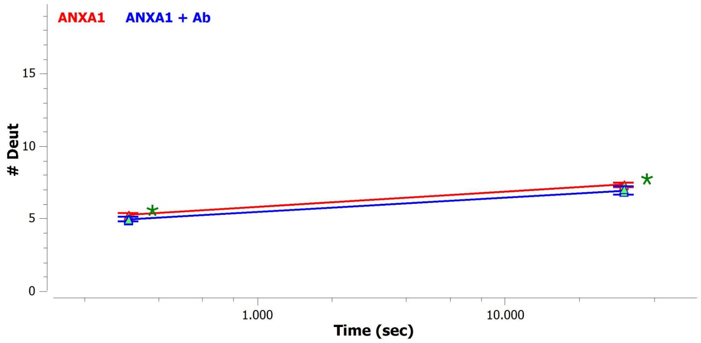


### 96-107: TLKKALTGHLEE (#58)

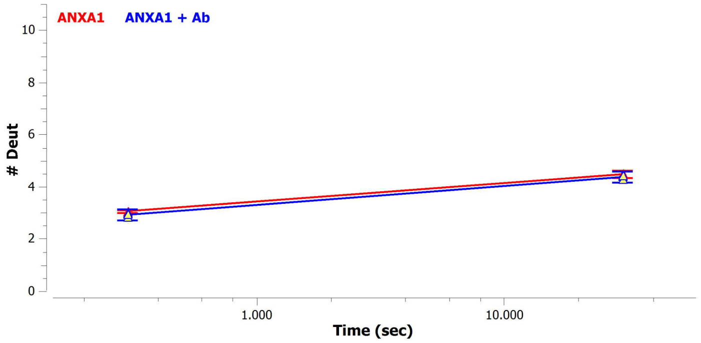


### 106-110: EEVVL (#59)

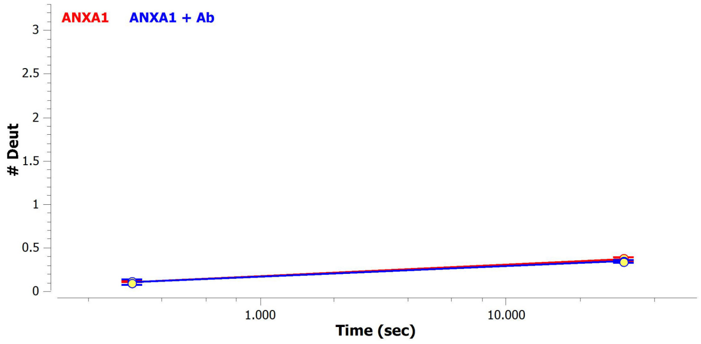


### 108-112: VVLAL (#60)

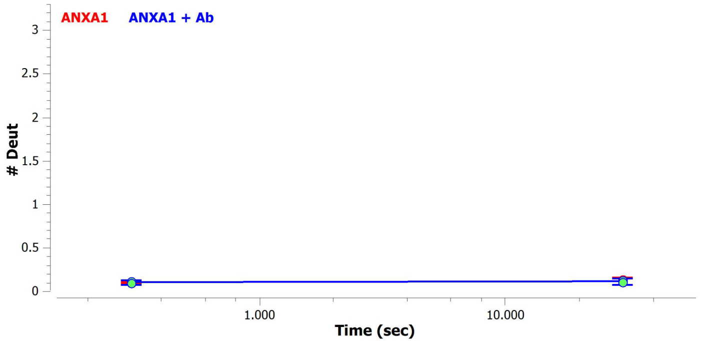

108-138: VVLALLKTPAQFDADELRAAMKGLGTDEDTL (#61)

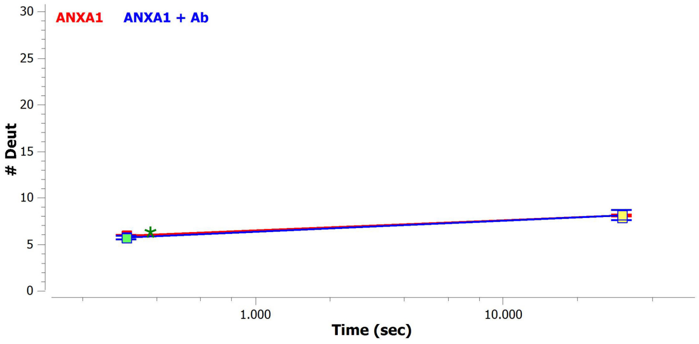


111-118: ALLKTPAQ (#62)

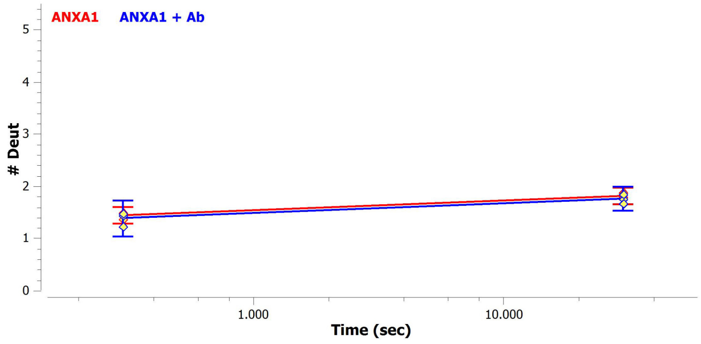


111-119: ALLKTPAQF (#63)

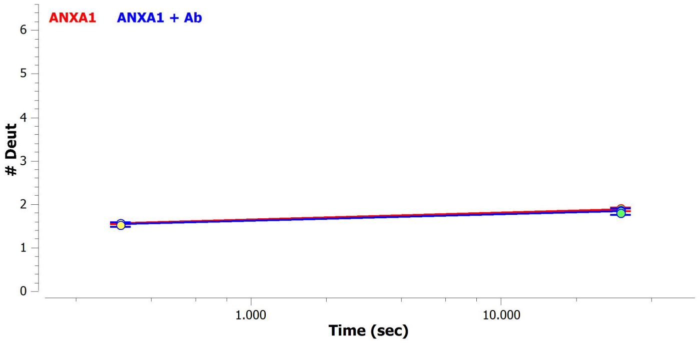


111-123: ALLKTPAQFDADE (#64)

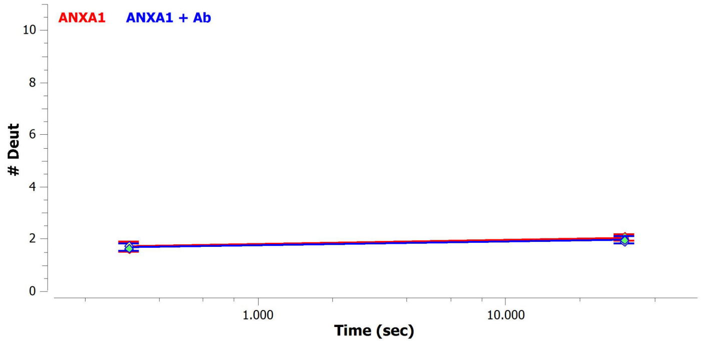


111-124: ALLKTPAQFDADEL (#65)

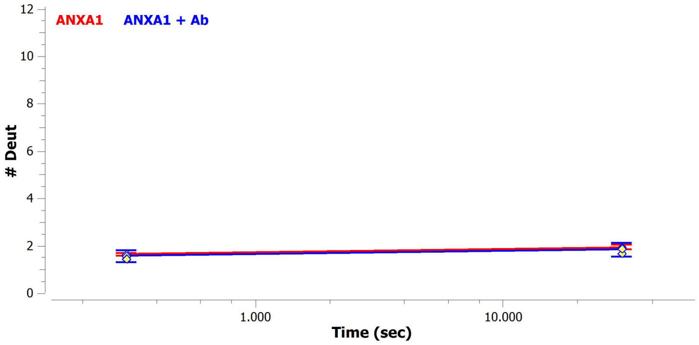


111-138: ALLKTPAQFDADELRAAMKGLGTDEDTL (#66)

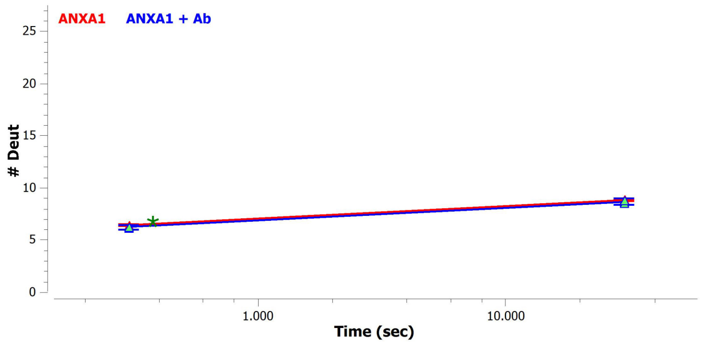


113-119: LKTPAQF (#67)

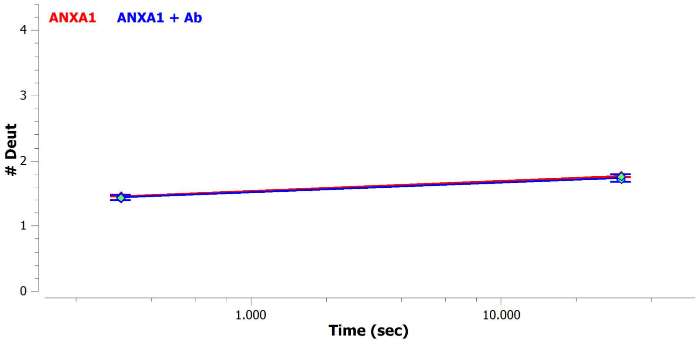


113-124: LKTPAQFDADEL (#68)

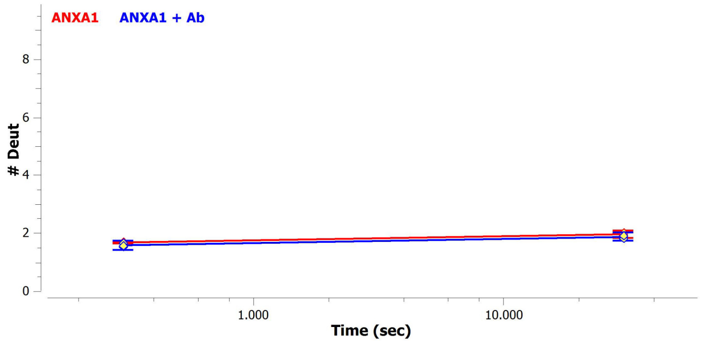


113-138: LKTPAQFDADELRAAMKGLGTDEDTL (#69)

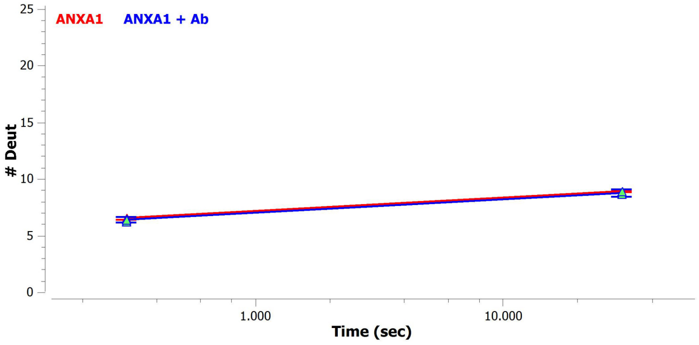


120-124: DADEL (#70)

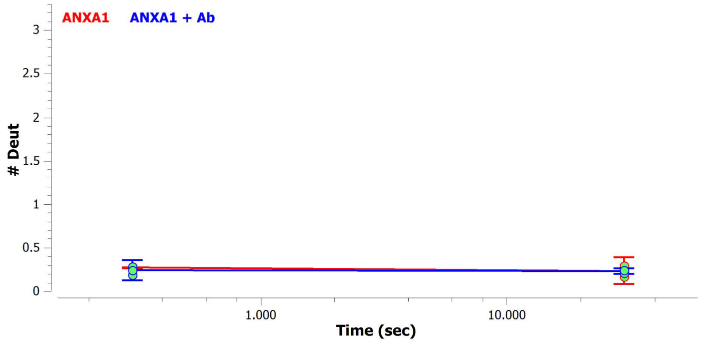

120-126: DADELRA (#71)

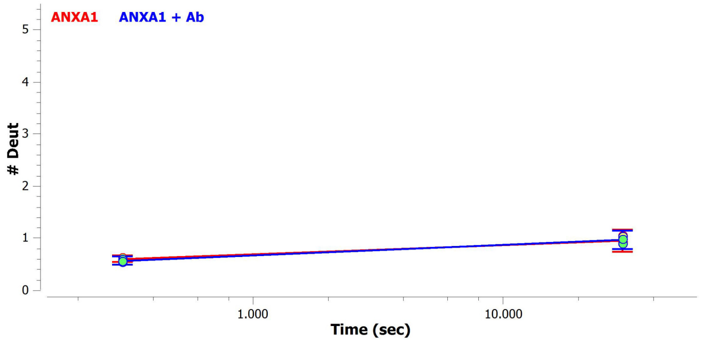


120-138: DADELRAAMKGLGTDEDTL (#72)

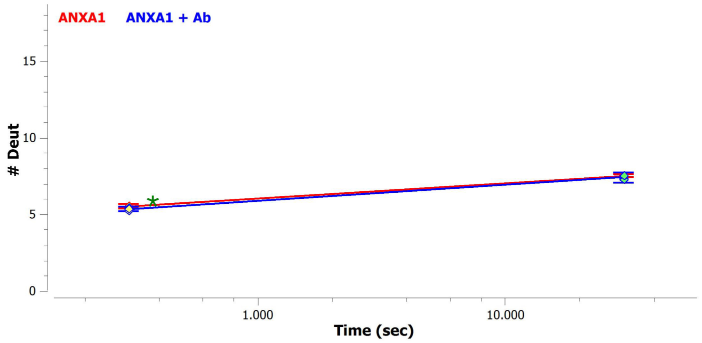


123-138: ELRAAMKGLGTDEDTL (#73)

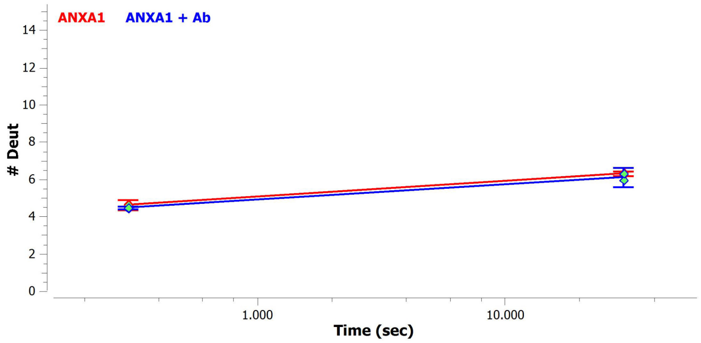


124-138: LRAAMKGLGTDEDTL (#74)

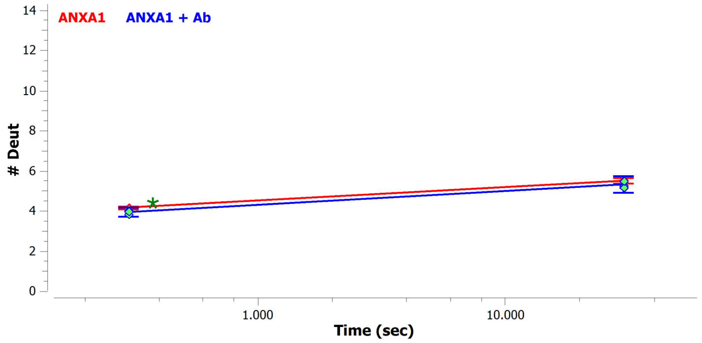


125-138: RAAMKGLGTDEDTL (#75)

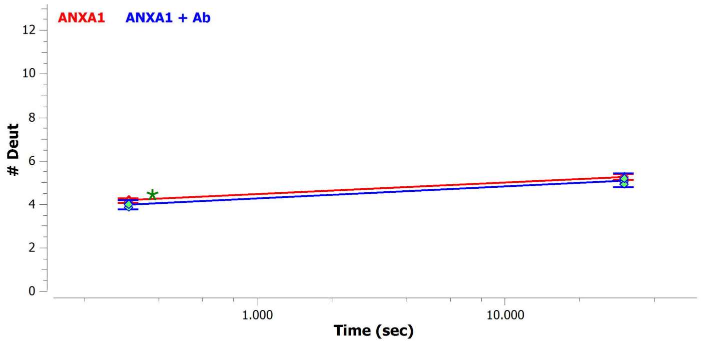


127-138: AMKGLGTDEDTL (#76)

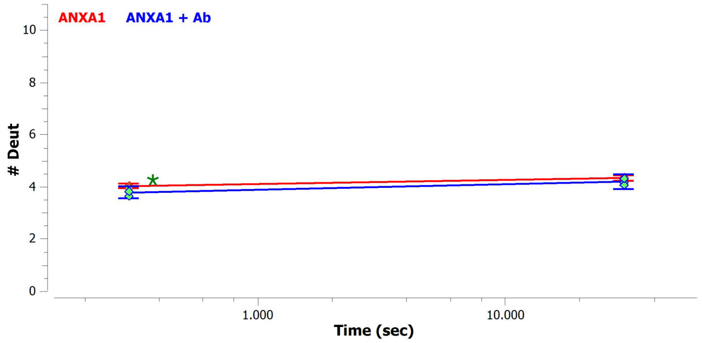


129-138: KGLGTDEDTL (#77)

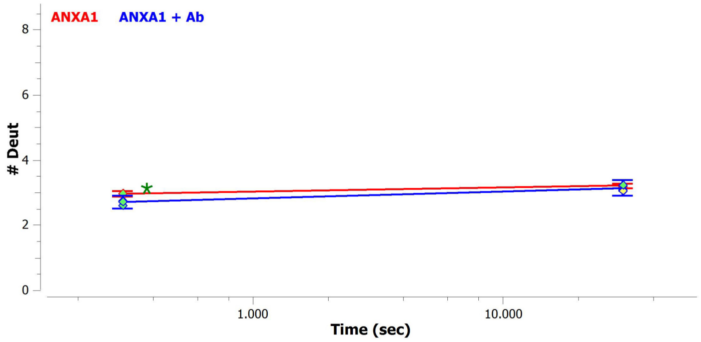


138-142: LIEIL (#78)

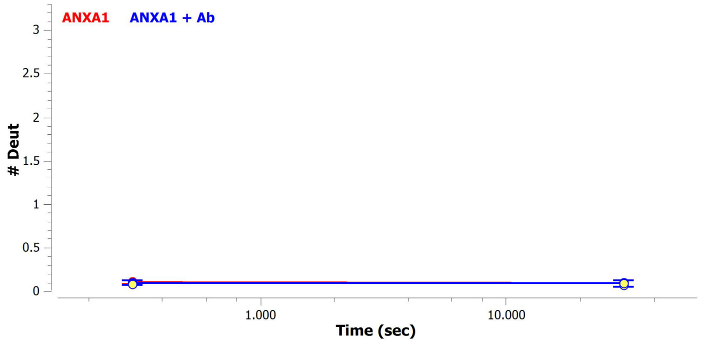


139-142: IEIL (#79)

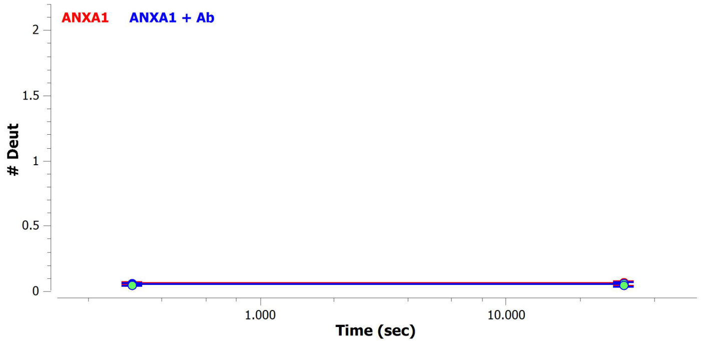


139-181: IEILASRTNKEIRDINRVYREELKRDLAKDITSDTSGDFRNAL (#80)

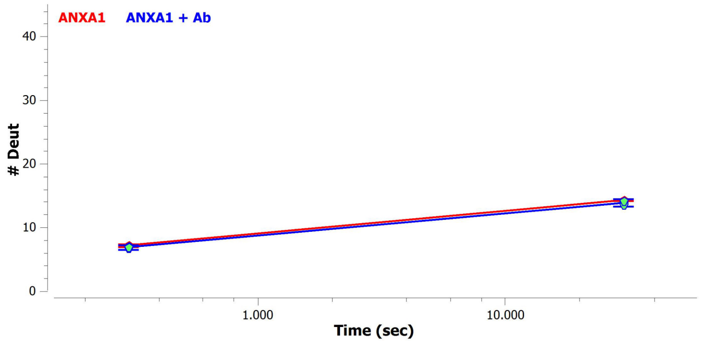

143-156: ASRTNKEIRDINRV (#81)

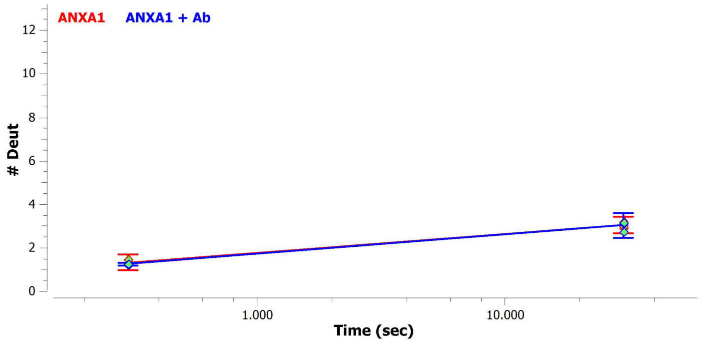


143-157: ASRTNKEIRDINRVY (#82)

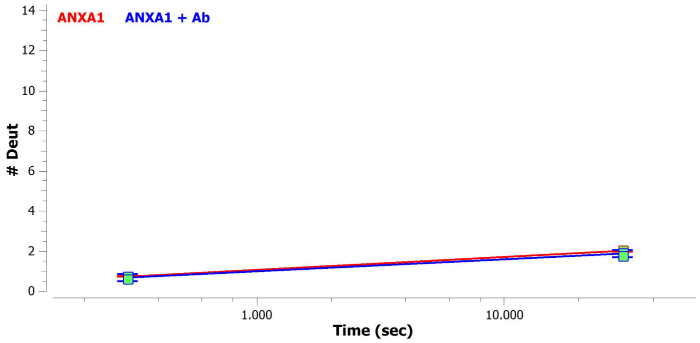


143-159: ASRTNKEIRDINRVYRE (#83)

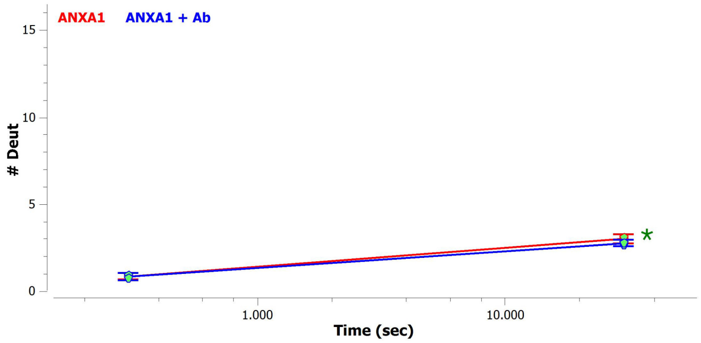


143-161: ASRTNKEIRDINRVYREEL (#84)

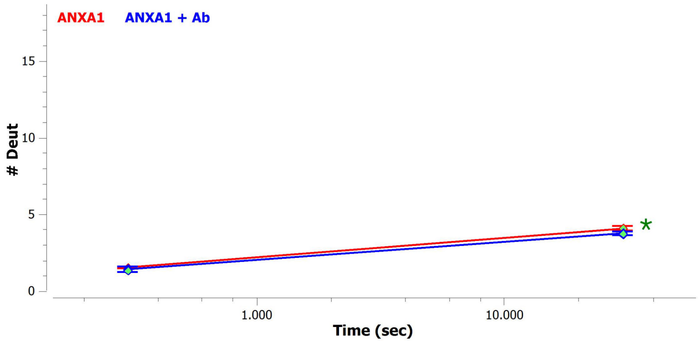


143-168: ASRTNKEIRDINRVYREELKRDLAKD (#85)

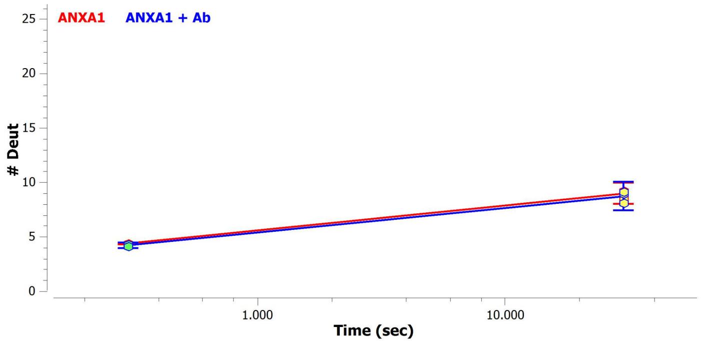


143-176: ASRTNKEIRDINRVYREELKRDLAKDITSDTSGD (#86)

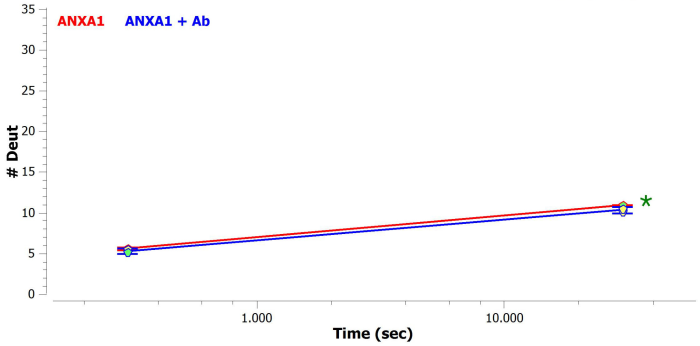


143-177: ASRTNKEIRDINRVYREELKRDLAKDITSDTSGDF (#87)

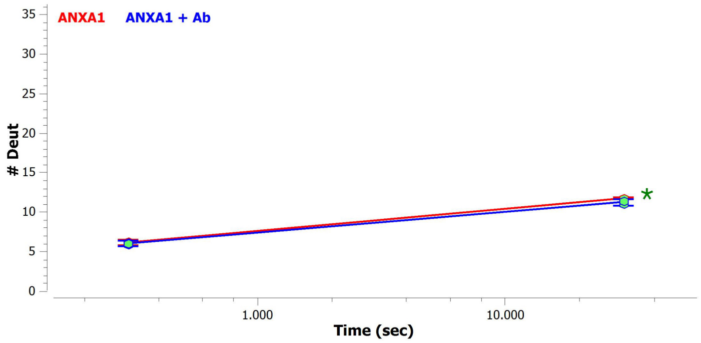


143-181: ASRTNKEIRDINRVYREELKRDLAKDITSDTSGDFRNAL (#88)

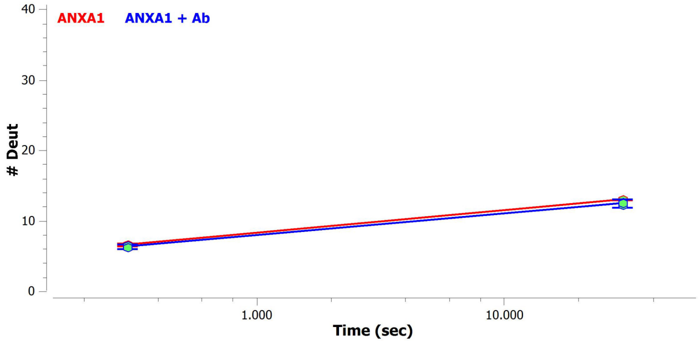


143-182: ASRTNKEIRDINRVYREELKRDLAKDITSDTSGDFRNALL (#89)

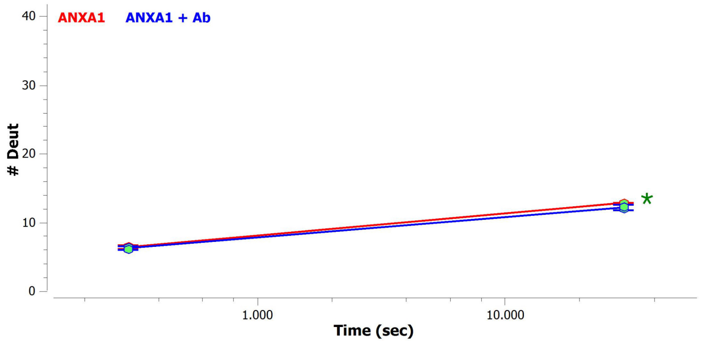


150-161: IRDINRVYREEL (#90)

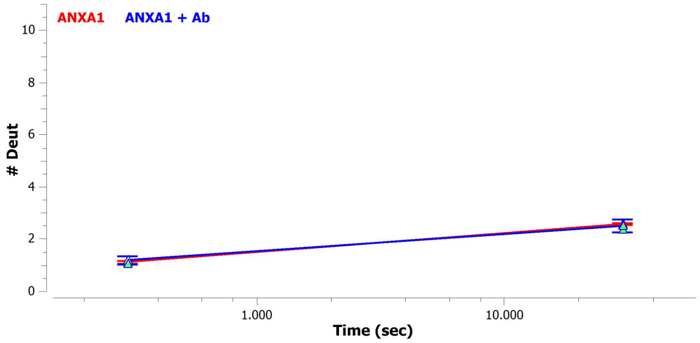

**153-161: INRVYREEL (#91)**

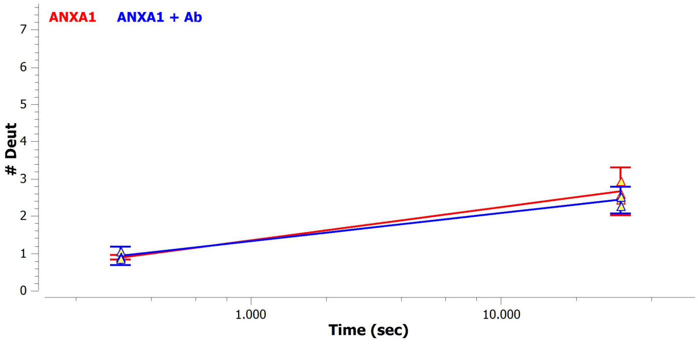


**157-161: YREEL (#92)**

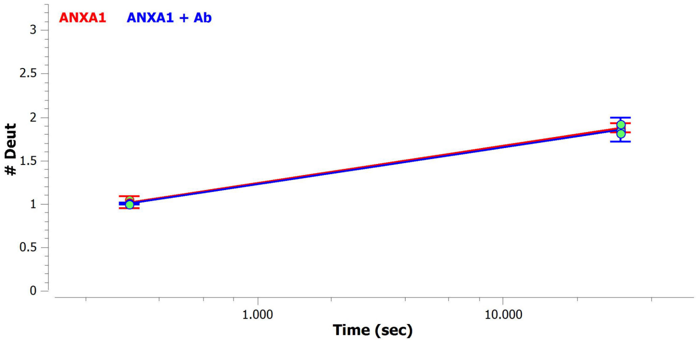


**157-177: YREELKRDLAKDITSDTSGDF (#93)**

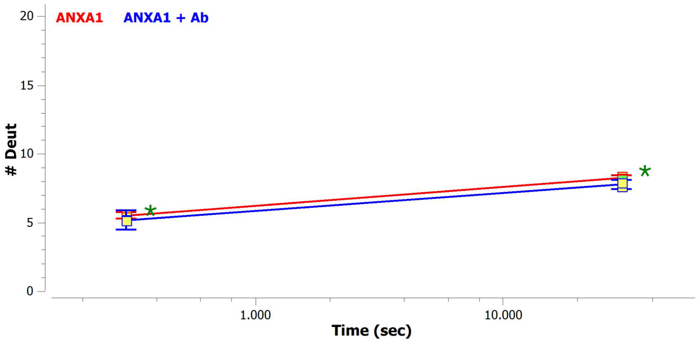


**157-181: YREELKRDLAKDITSDTSGDFRNAL (#94)**

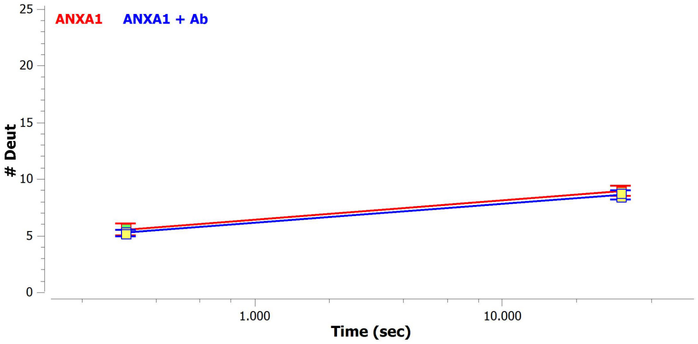


**158-181: REELKRDLAKDITSDTSGDFRNAL (#95)**

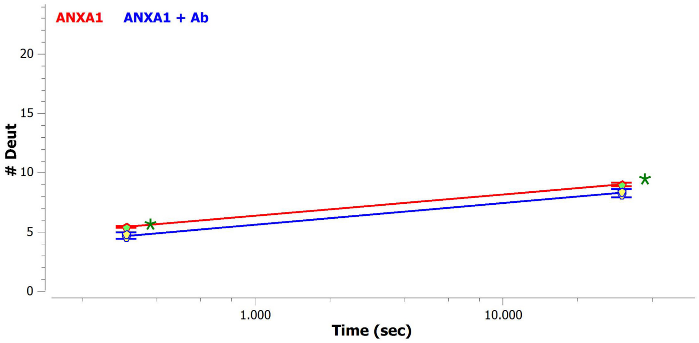


**162-176: KRDLAKDITSDTSGD (#96)**

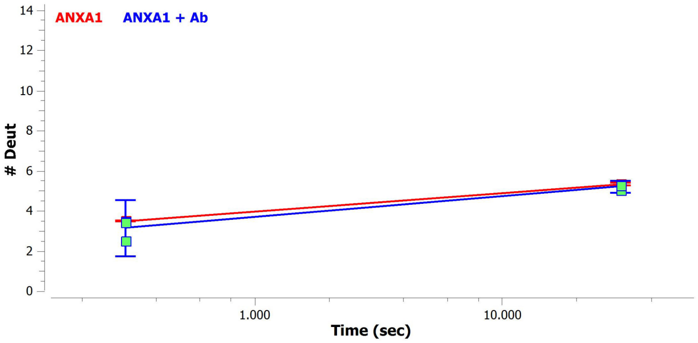


**162-177: KRDLAKDITSDTSGDF (#97)**

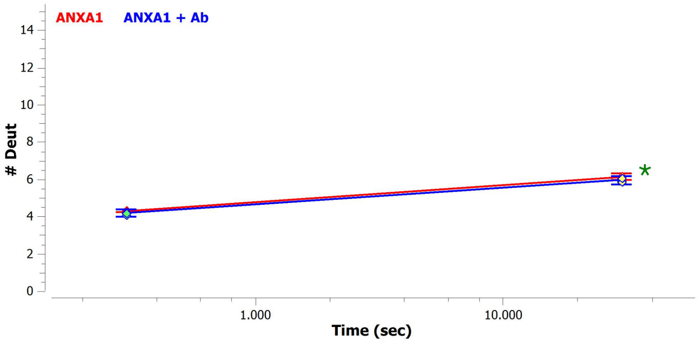


**162-181: KRDLAKDITSDTSGDFRNAL (#98)**

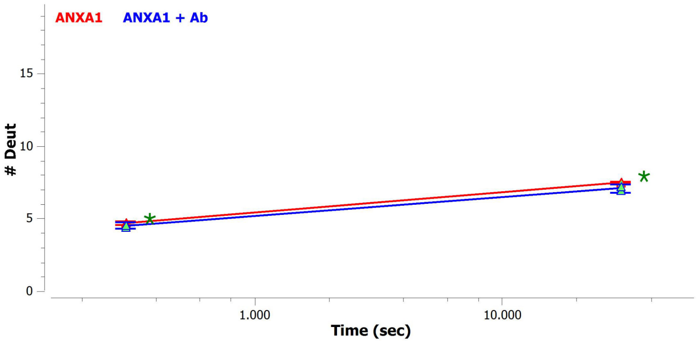


**169-177: ITSDTSGDF (#99)**

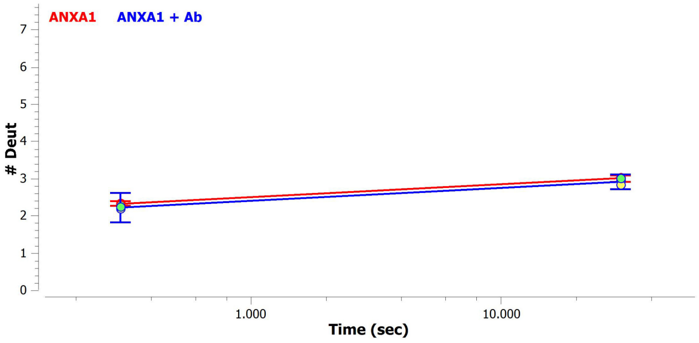


**169-181: ITSDTSGDFRNAL (#100)**

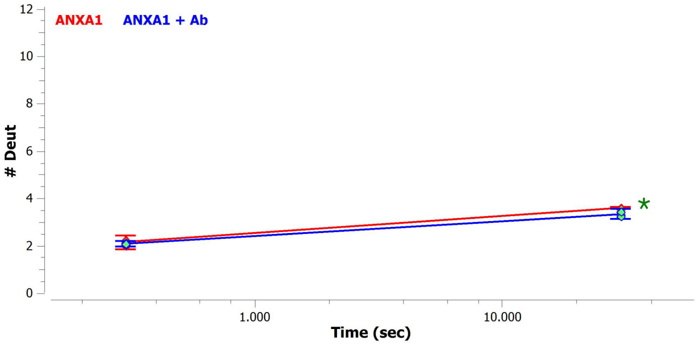

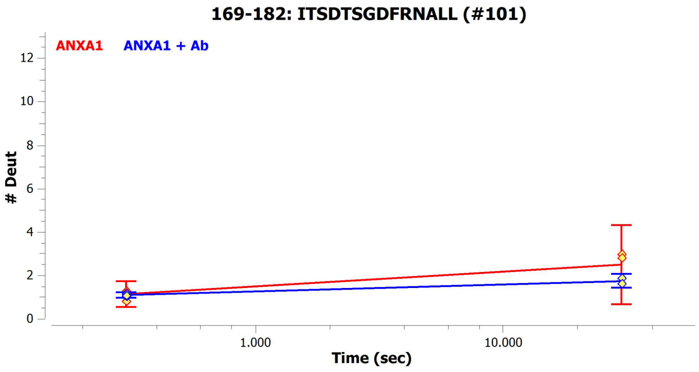
169-182: ITSDTSGDFRNALL (#101)
ANXA1
ANXA1 + Ab
# Deut
0
2
4
6
8
10
12
1.000
10.000
Time (sec)

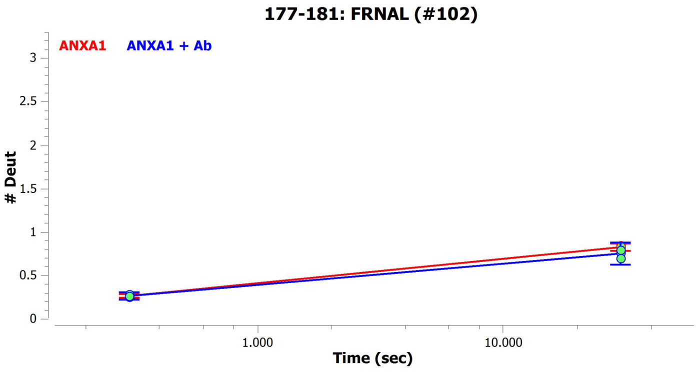
177-181: FRNAL (#102)
ANXA1
ANXA1 + Ab
# Deut
0
0.5
1
1.5
2
2.5
3
1.000
10.000
Time (sec)

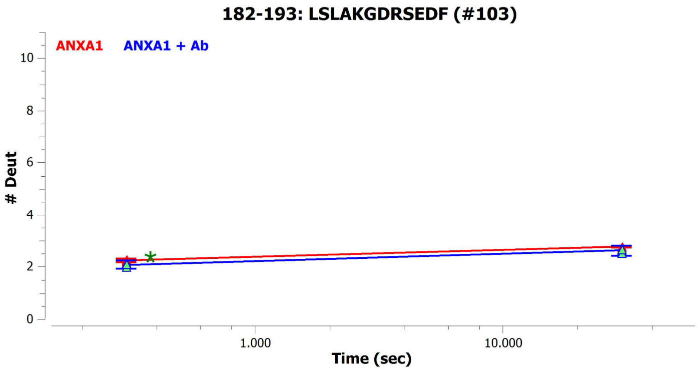
182-193: LSLAKGDRSEDF (#103)
ANXA1
ANXA1 + Ab
# Deut
0
2
4
6
8
10
1.000
10.000
Time (sec)

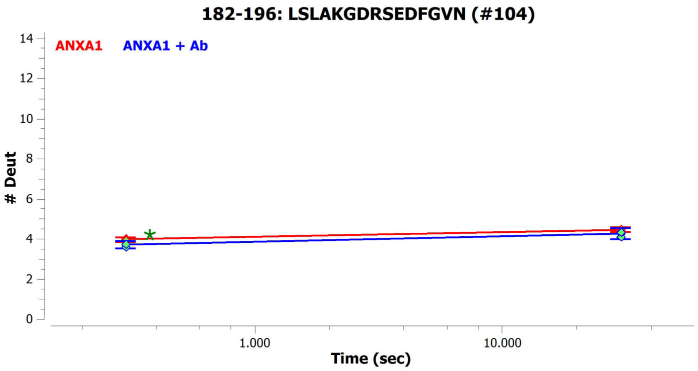
182-196: LSLAKGDRSEDFGVN (#104)
ANXA1
ANXA1 + Ab
# Deut
0
2
4
6
8
10
12
14
1.000
10.000
Time (sec)

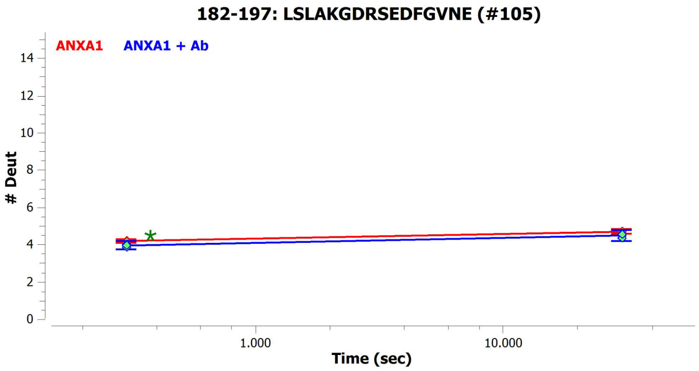
182-197: LSLAKGDRSEDFGVNE (#105)
ANXA1
ANXA1 + Ab
# Deut
0
2
4
6
8
10
12
14
1.000
10.000
Time (sec)

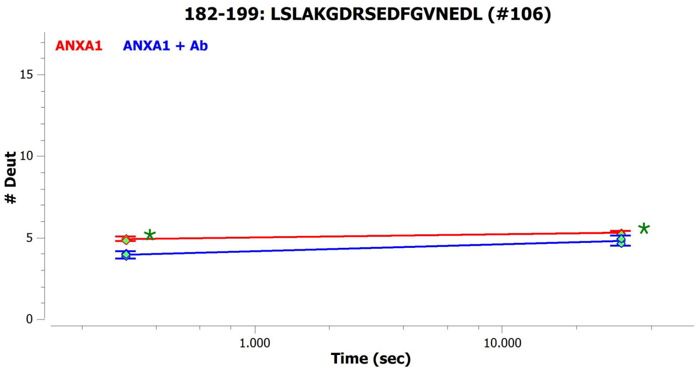
182-199: LSLAKGDRSEDFGVNEDL (#106)
ANXA1
ANXA1 + Ab
# Deut
0
5
10
15
1.000
10.000
Time (sec)

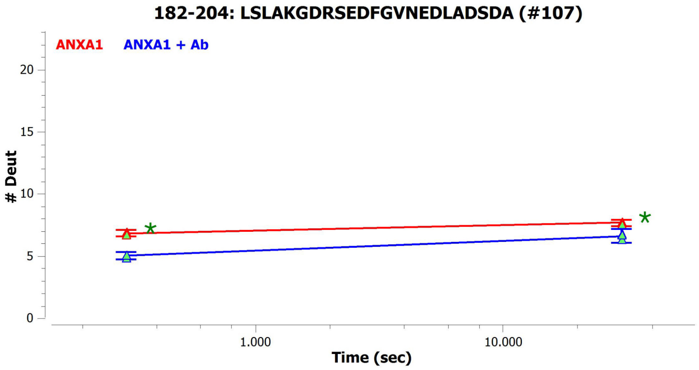
182-204: LSLAKGDRSEDFGVNEDLADSDA (#107)
ANXA1
ANXA1 + Ab
# Deut
0
5
10
15
20
1.000
10.000
Time (sec)

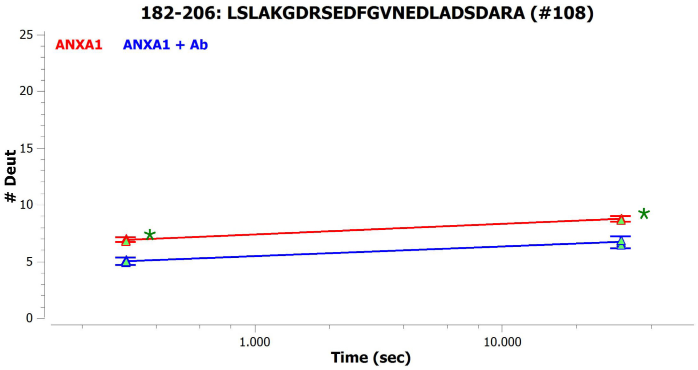
182-206: LSLAKGDRSEDFGVNEDLADSDARA (#108)
ANXA1
ANXA1 + Ab
# Deut
0
5
10
15
20
25
1.000
10.000
Time (sec)

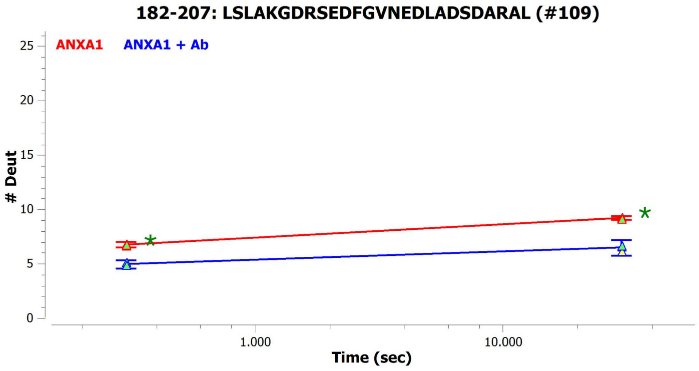
182-207: LSLAKGDRSEDFGVNEDLADSDARAL (#109)
ANXA1
ANXA1 + Ab
# Deut
0
5
10
15
20
25
1.000
10.000
Time (sec)

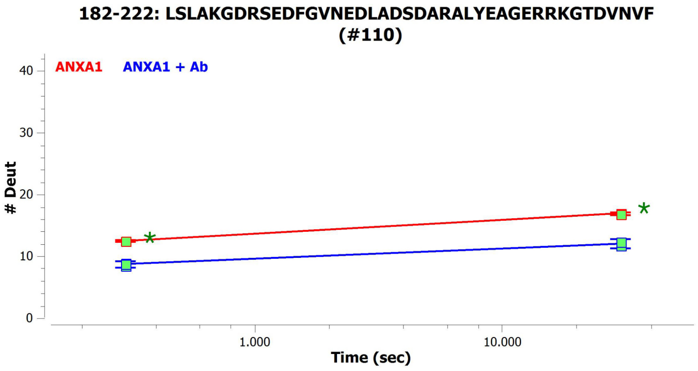
182-222: LSLAKGDRSEDFGVNEDLADSDARALYEAGERRKGTDVNVF (#110)
ANXA1
ANXA1 + Ab
# Deut
0
10
20
30
40
1.000
10.000
Time (sec)

182-226: LSLAKGDRSEDFGVNEDLADSDARALYEAGERRKGTDVNVFNTIL (#111)

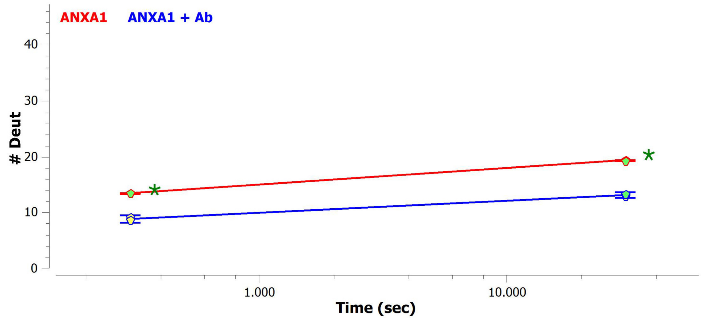


183-196: SLAKGDRSEDFGVN (#112)

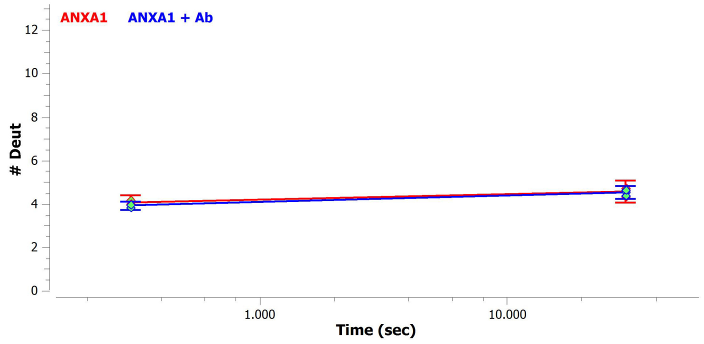


183-199: SLAKGDRSEDFGVNEDL (#113)

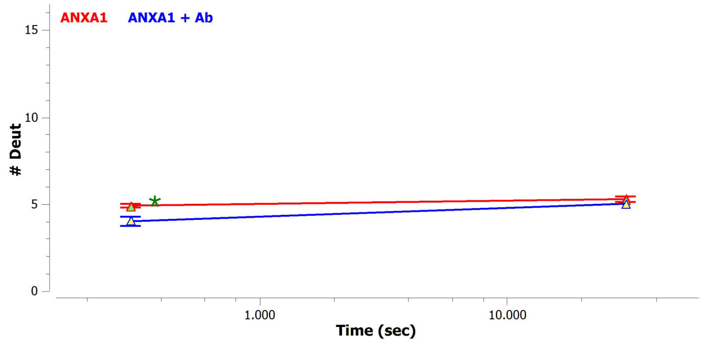


183-204: SLAKGDRSEDFGVNEDLADSDA (#114)

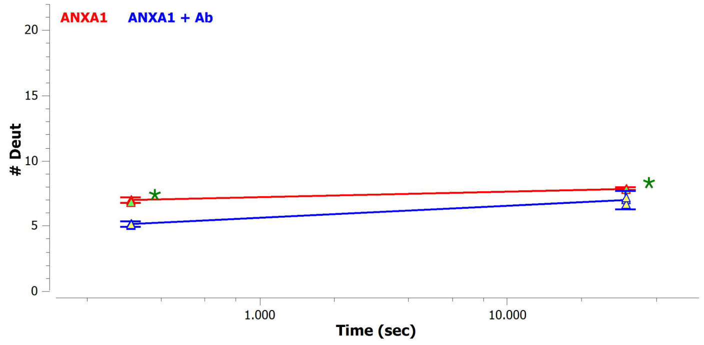


185-196: AKGDRSEDFGVN (#115)

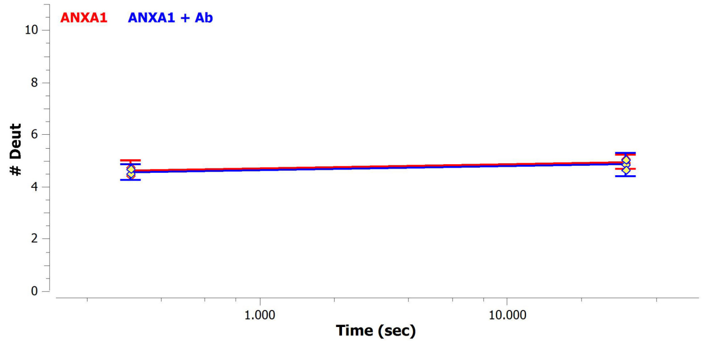


185-197: AKGDRSEDFGVNE (#116)

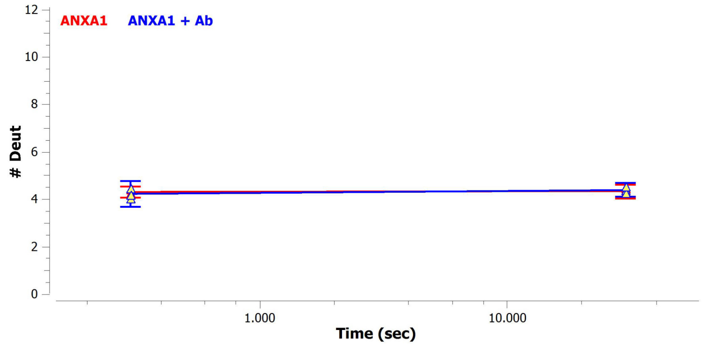


185-199: AKGDRSEDFGVNEDL (#117)

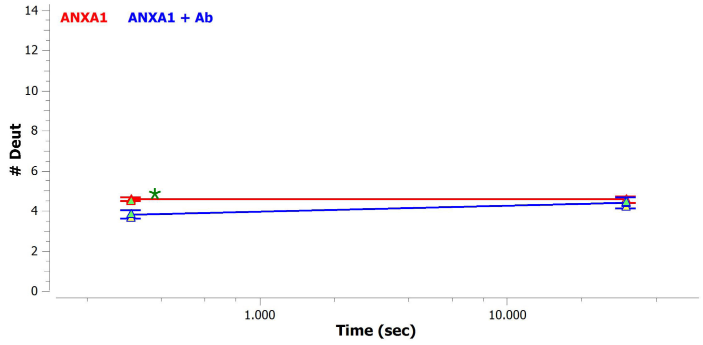


193-196: FGVN (#118)

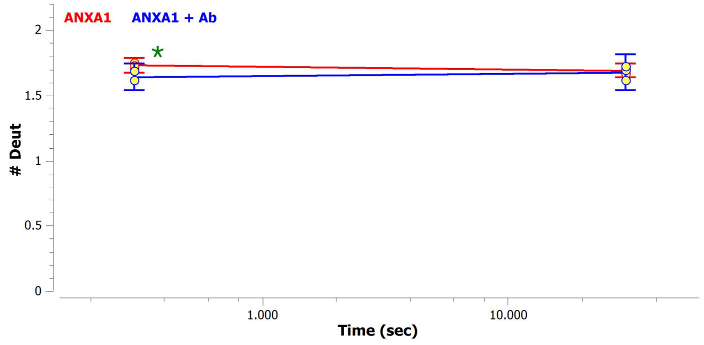


193-197: FGVNE (#119)

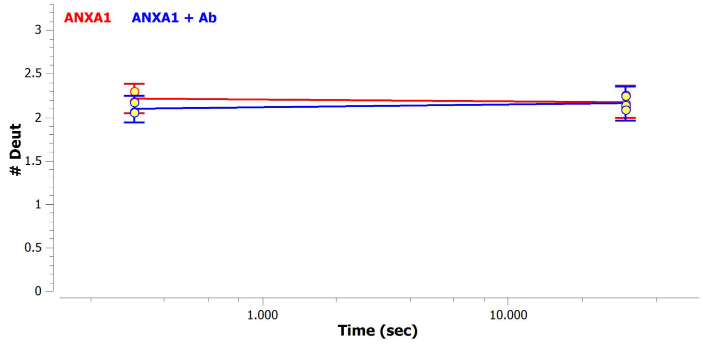


193-199: FGVNEDL (#120)

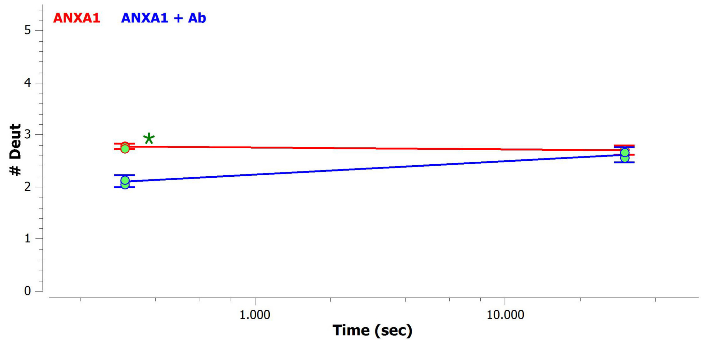

193-204: FGVNEDLADSDA (#121)

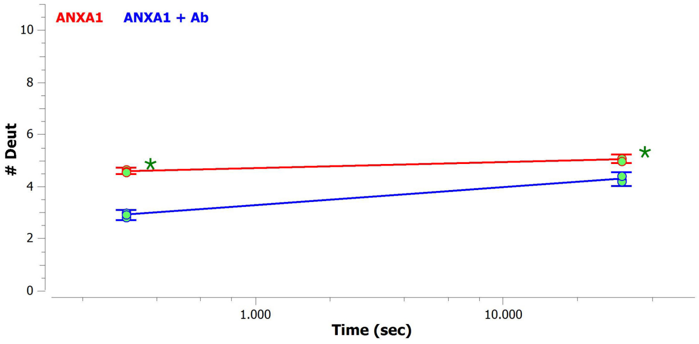


193-206: FGVNEDLADSDARA (#122)

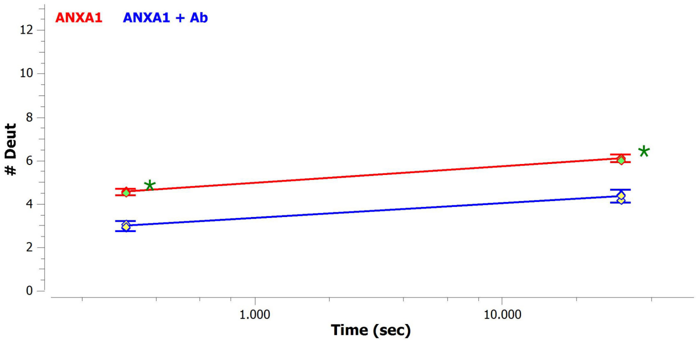


193-207: FGVNEDLADSDARAL (#123)

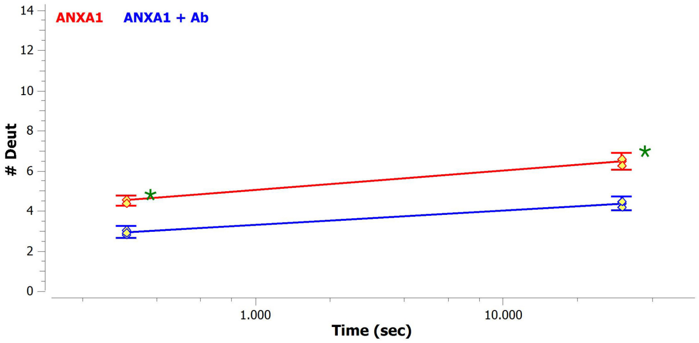


194-199: GVNEDL (#124)

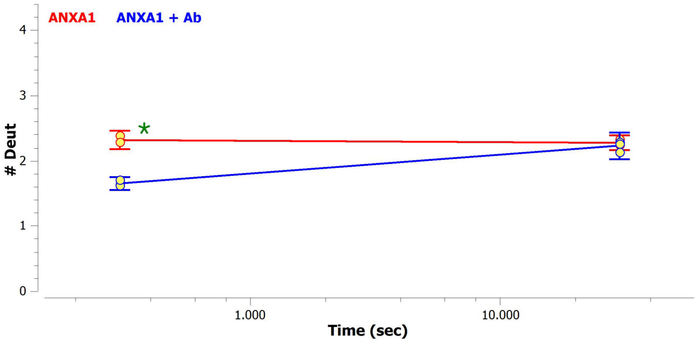


194-204: GVNEDLADSDA (#125)

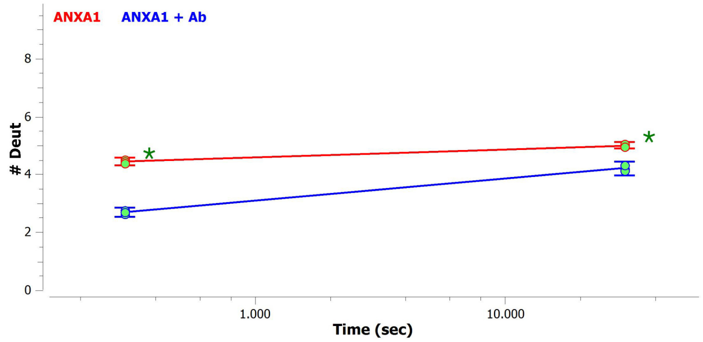


194-206: GVNEDLADSDARA (#126)

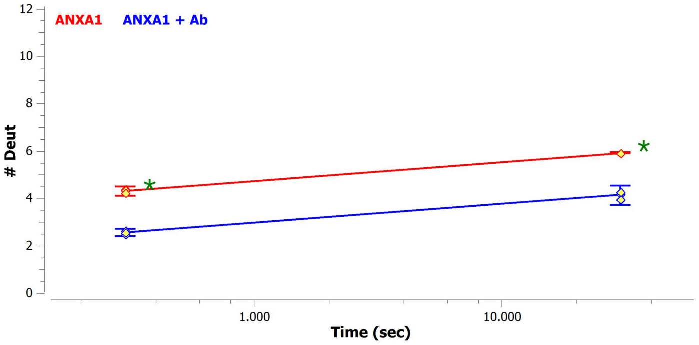


197-204: EDLADSDA (#127)

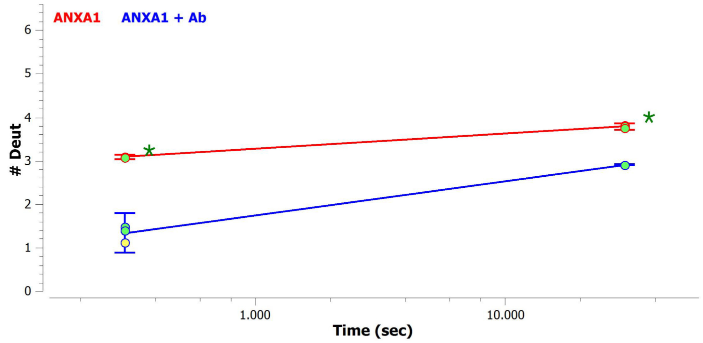


197-206: EDLADSDARA (#128)

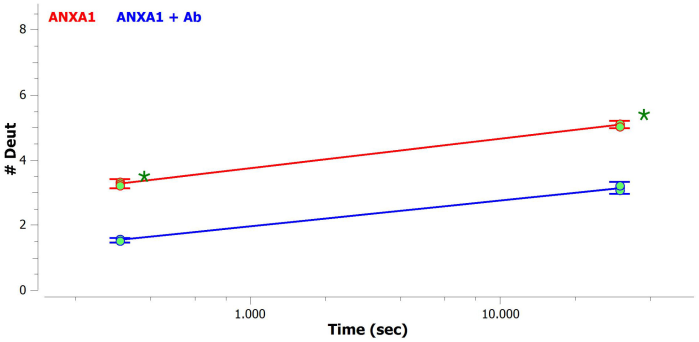


197-207: EDLADSDARAL (#129)

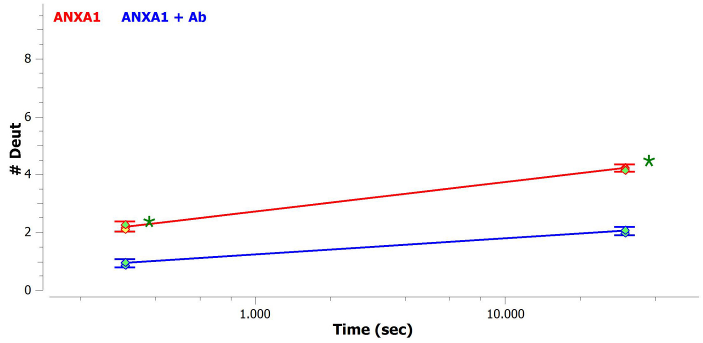


197-222: EDLADSDARALYEAGERRKGTDVNVF (#130)

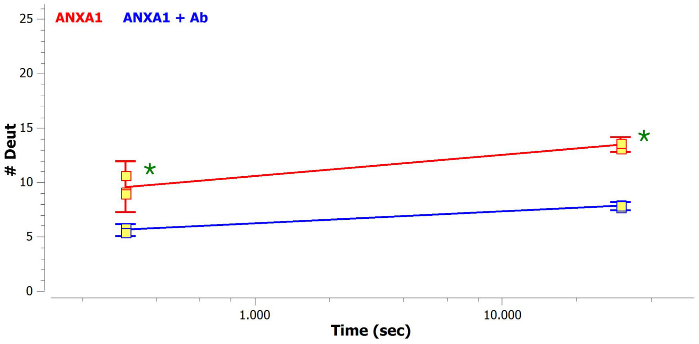

198-207: DLADSDARAL (#131)

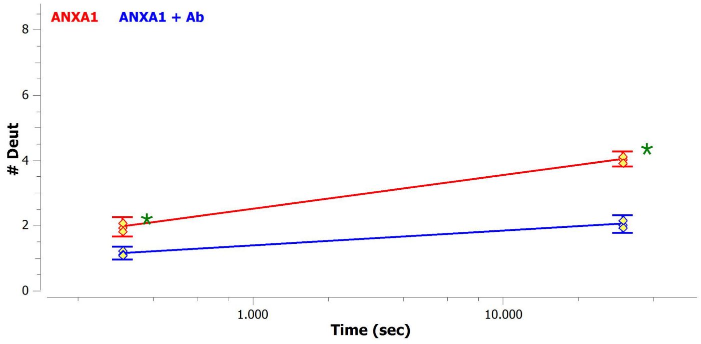


198-222: DLADSDARALYEAGERRKGTDVNVF (#132)

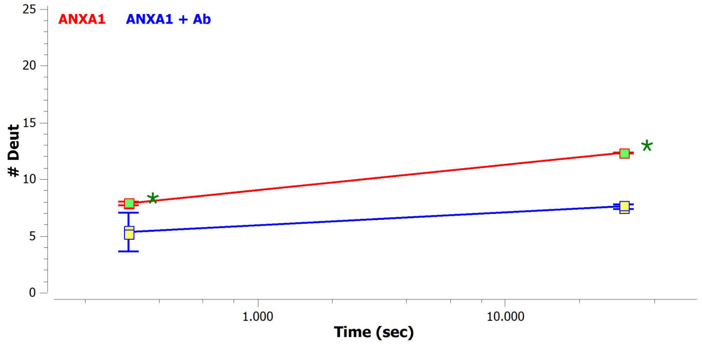


199-207: LADSDARAL (#133)

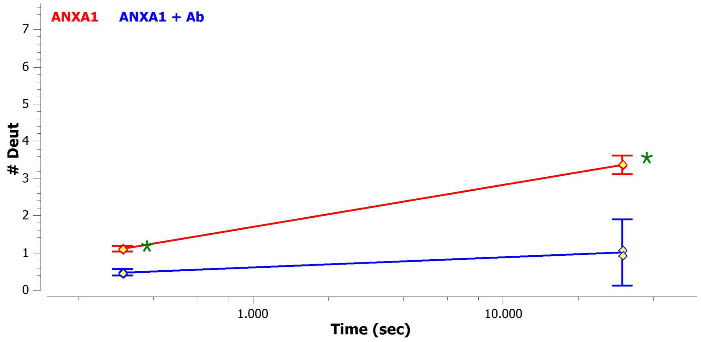


200-218: ADSDARALYEAGERRKGTD (#134)

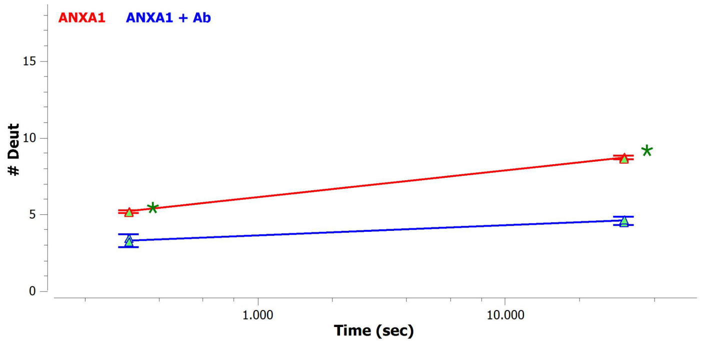


200-221: ADSDARALYEAGERRKGTDVNV (#135)

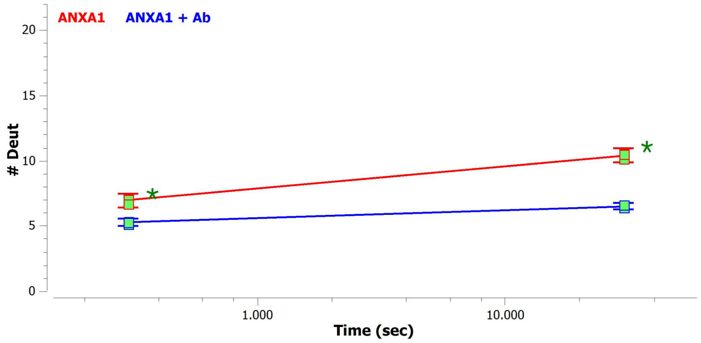


200-222: ADSDARALYEAGERRKGTDVNVF (#136)

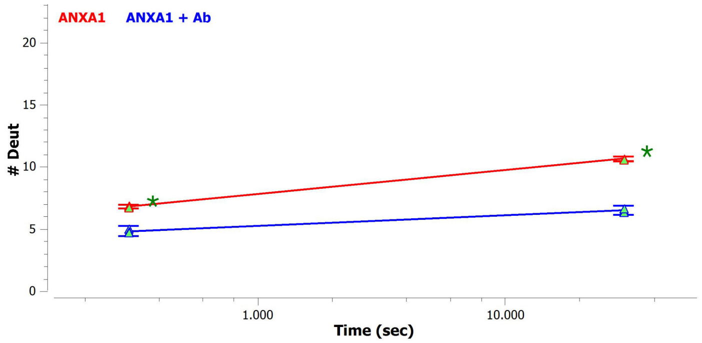


200-226: ADSDARALYEAGERRKGTDVNVFNTIL (#137)

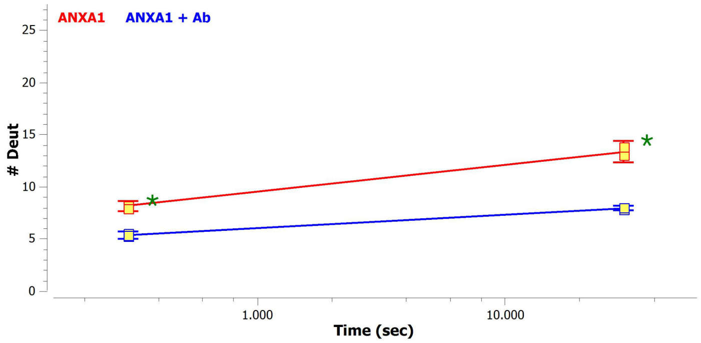


204-222: ARALYEAGERRKGTDVNVF (#138)

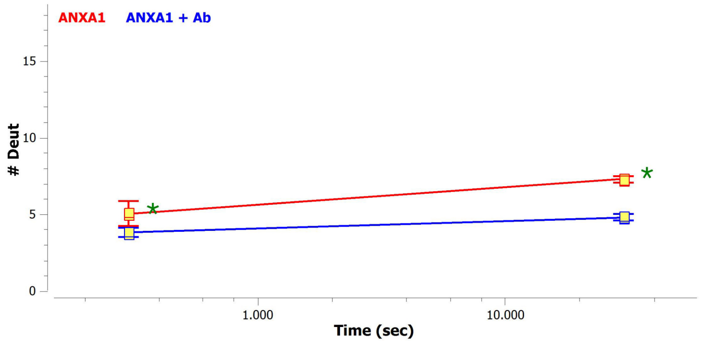


205-212: RALYEAGE (#139)

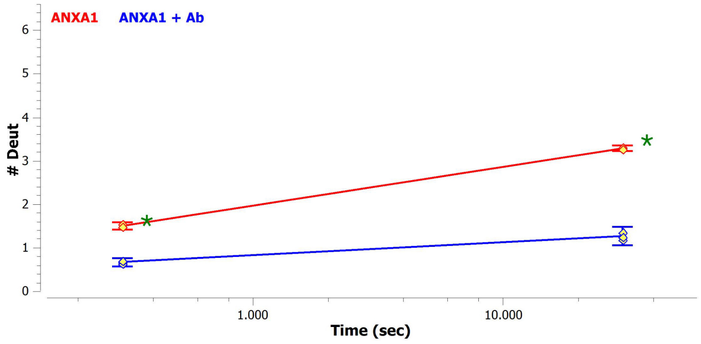


205-221: RALYEAGERRKGTDVNV (#140)

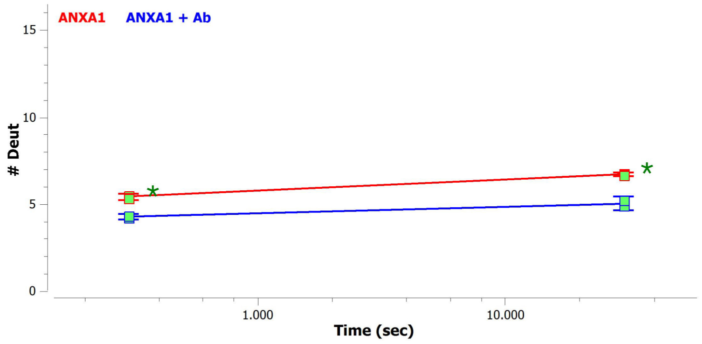

205-222: RALYEAGERRKGTDVNVF (#141)

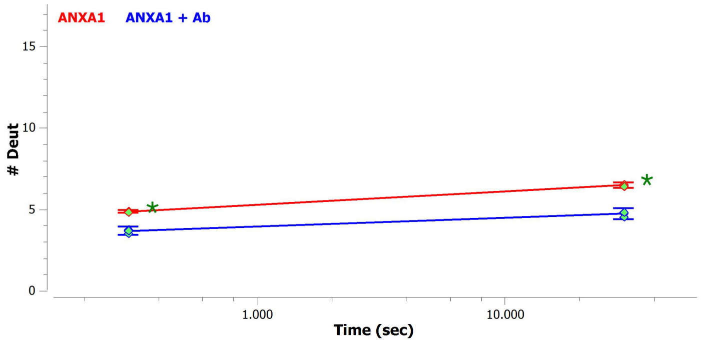


205-226: RALYEAGERRKGTDVNVFNTIL (#142)

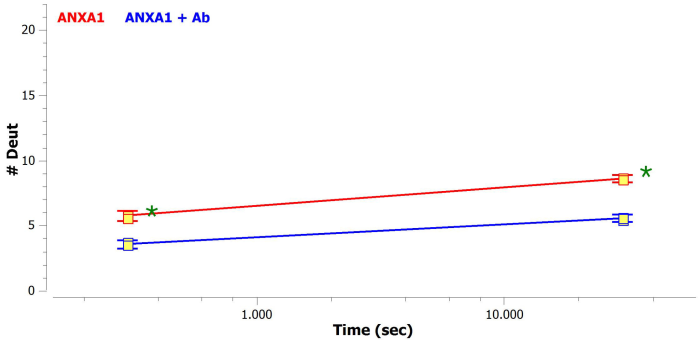


207-221: LYEAGERRKGTDVNV (#143)

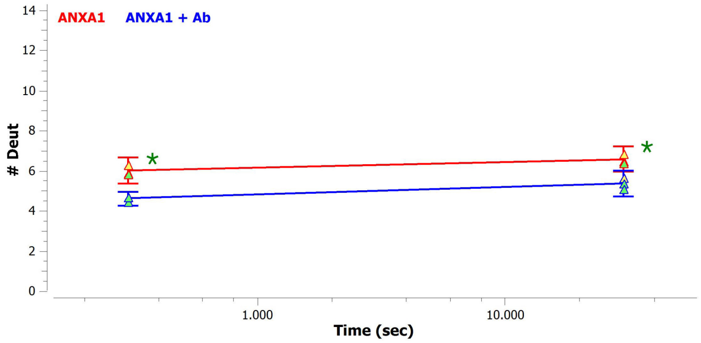


207-222: LYEAGERRKGTDVNVF (#144)

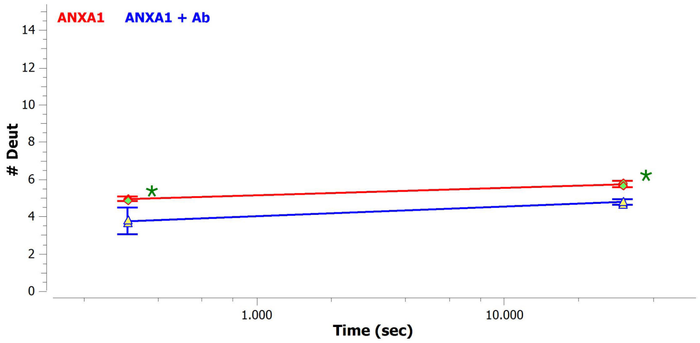


207-226: LYEAGERRKGTDVNVFNTIL (#145)

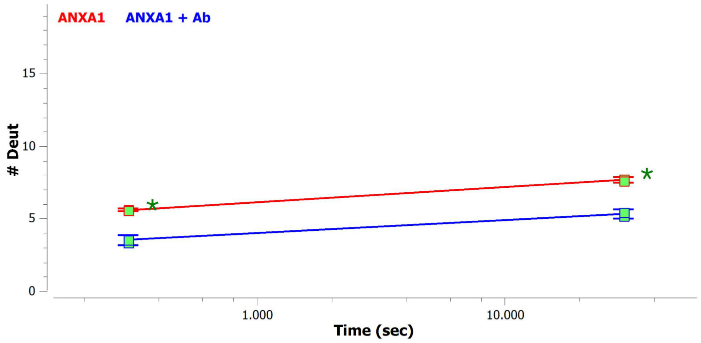


208-222: YEAGERRKGTDVNVF (#146)

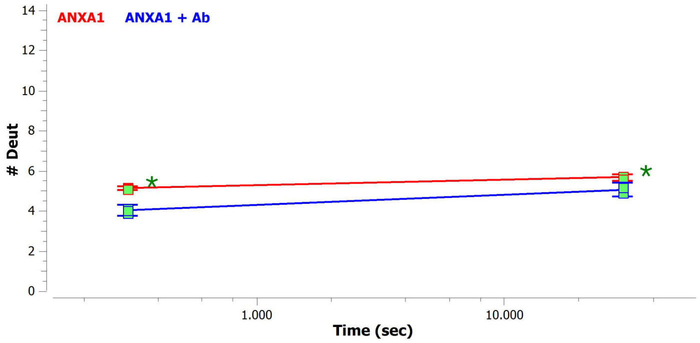


208-226: YEAGERRKGTDVNVFNTIL (#147)

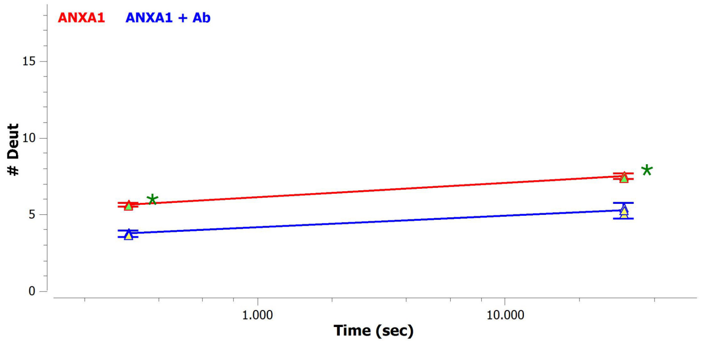


212-222: ERRKGTDVNVF (#148)

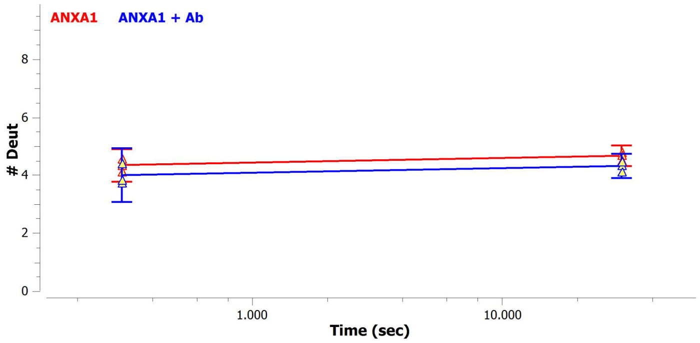


213-222: RRKGTDVNVF (#149)

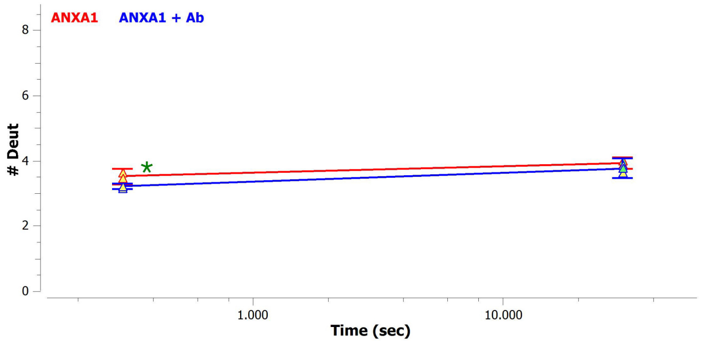


219-222: VNVF (#150)

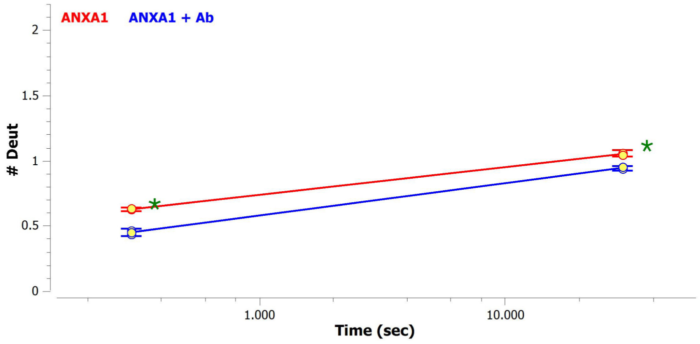

222-226: FNTIL (#151)

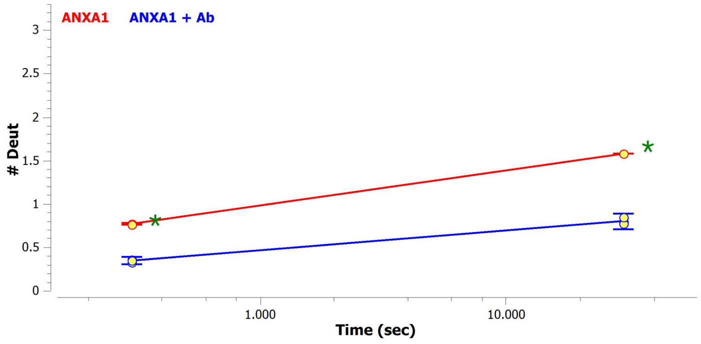


223-234: NTILTTRSYPQL (#152)

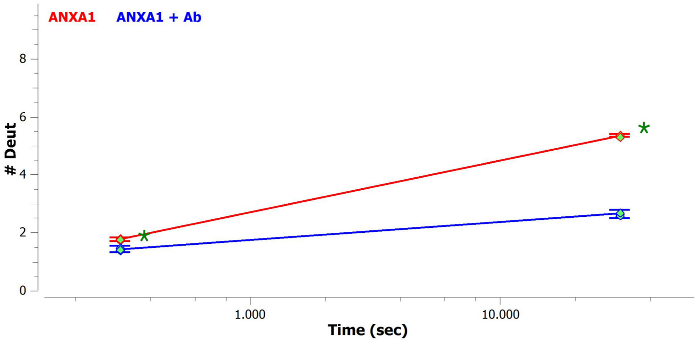


227-234: TTRSYPQL (#153)

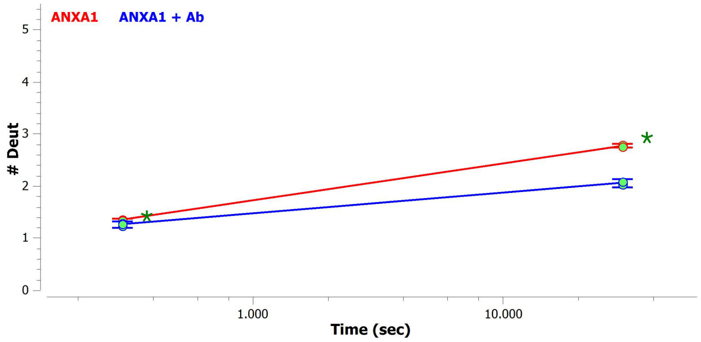


235-254: RRVFQKYTKYSKHDMNKVLD (#154)

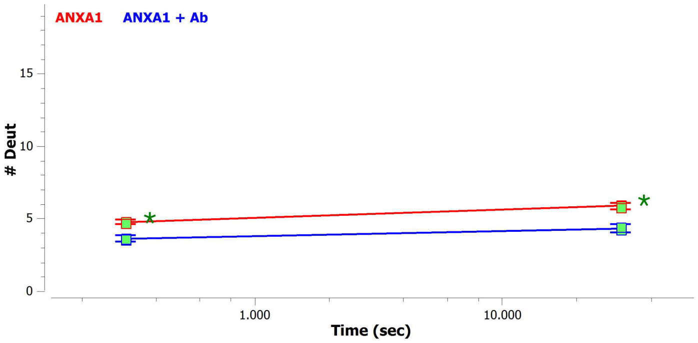


235-255: RRVFQKYTKYSKHDMNKVLDL (#155)

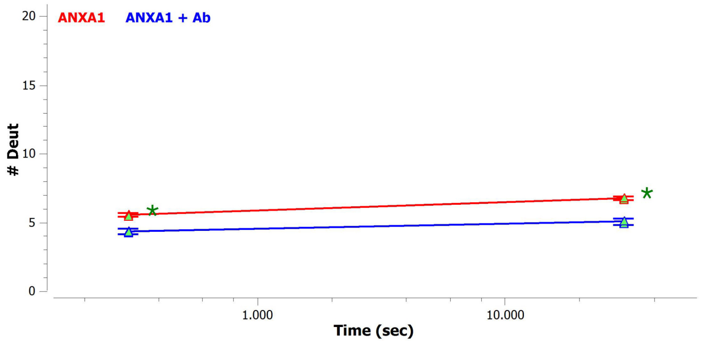


235-257: RRVFQKYTKYSKHDMNKVLDLEL (#156)

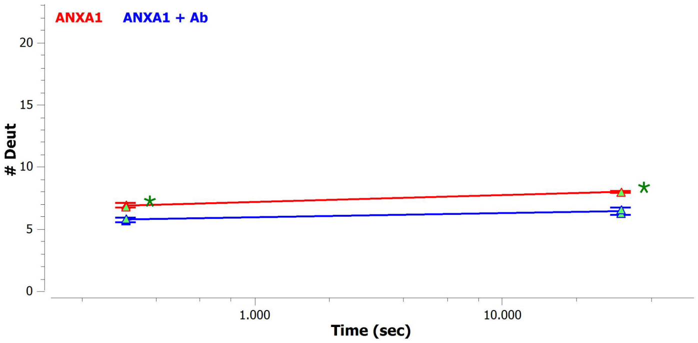


255-262: LELKGDIE (#157)

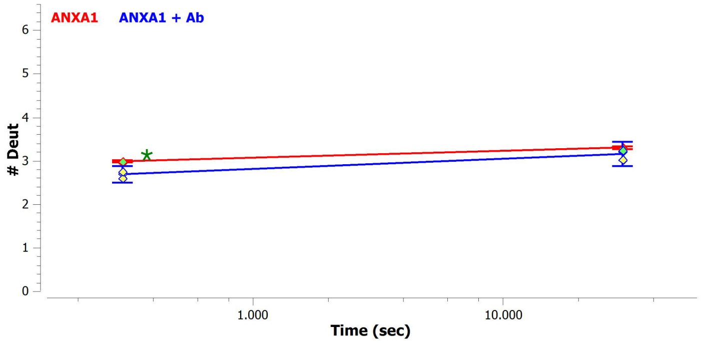


255-266: LELKGDIEKCLT (#158)

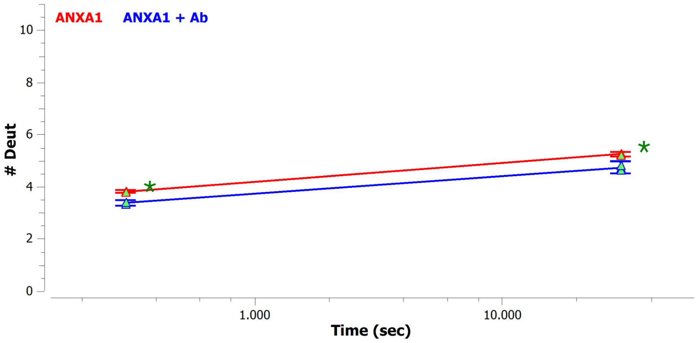


255-267: LELKGDIEKCLTA (#159)

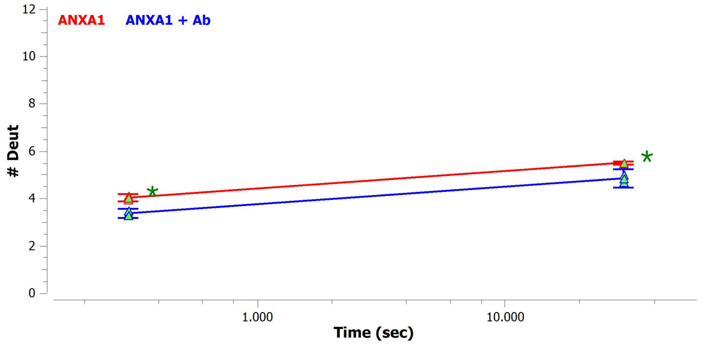


256-266: ELKGDIEKCLT (#160)

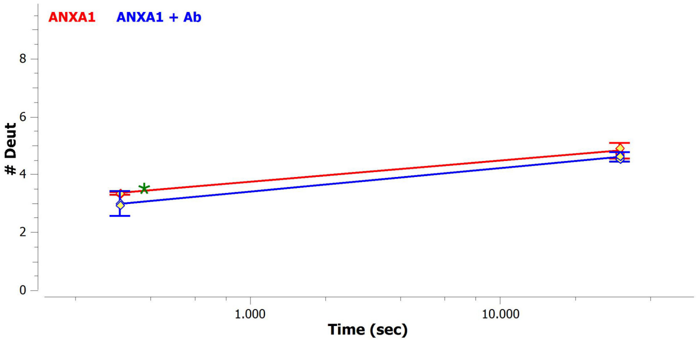

256-267: ELKGDIEKCLTA (#161)

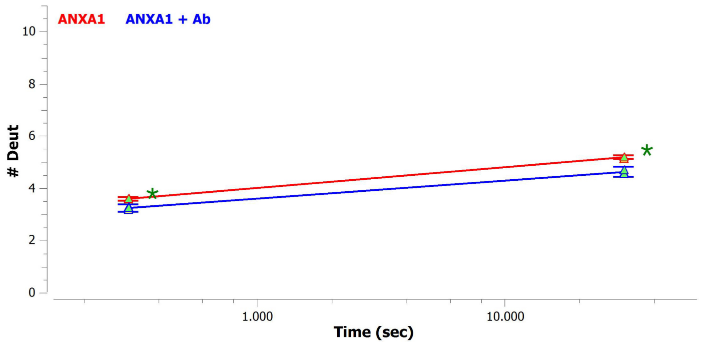


258-265: KGDIEKCL (#162)

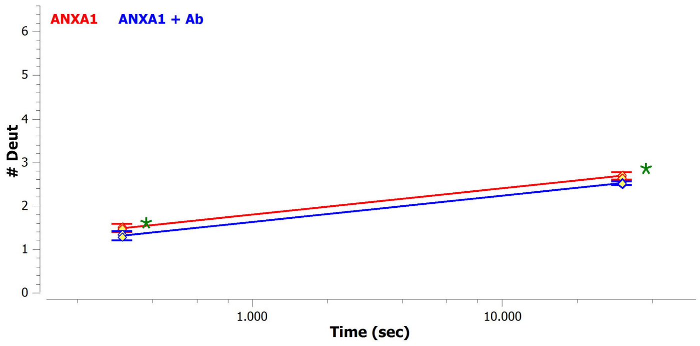


258-266: KGDIEKCLT (#163)

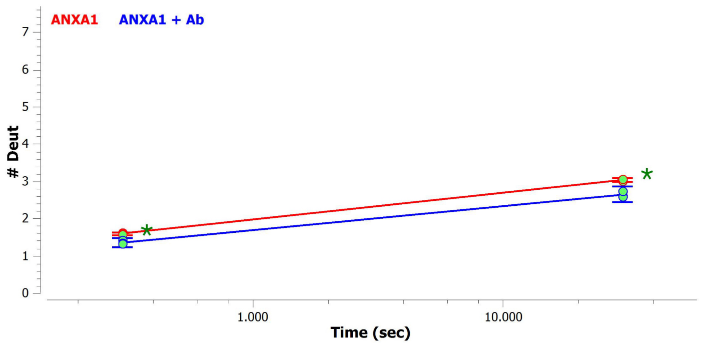


258-267: KGDIEKCLTA (#164)

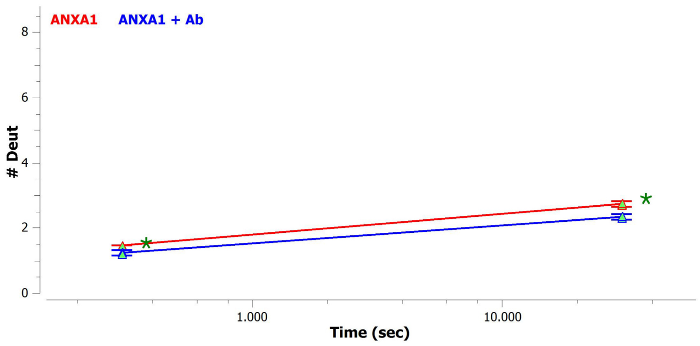


258-278: KGDIEKCLTAIVKCATSKPAF (#165)

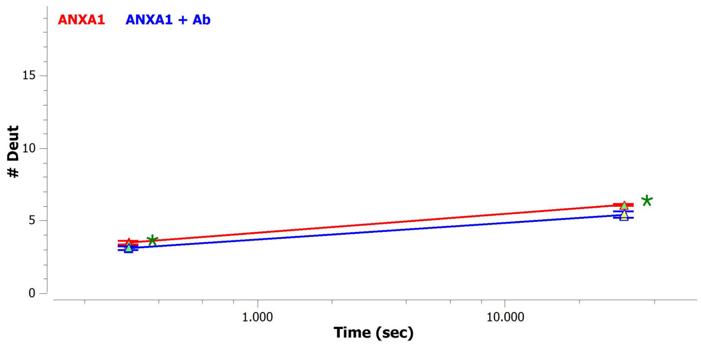


267-278: AIVKCATSKPAF (#166)

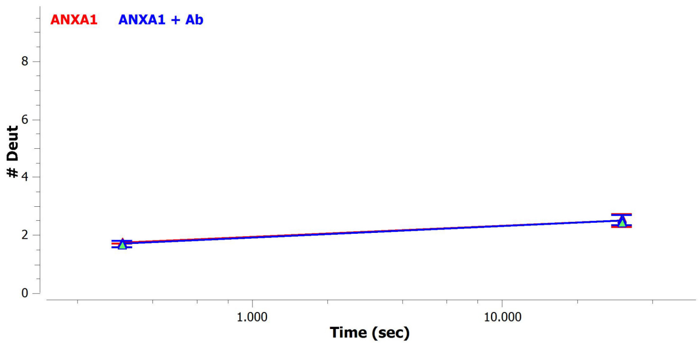


267-279: AIVKCATSKPAFF (#167)

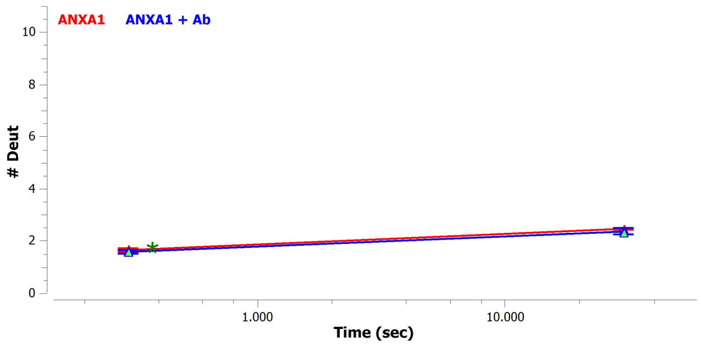


268-278: IVKCATSKPAF (#168)

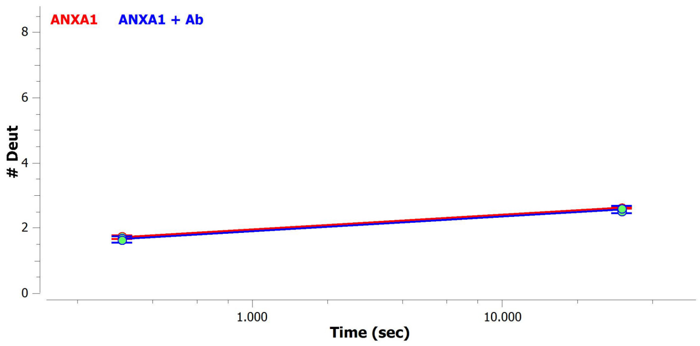


268-279: IVKCATSKPAFF (#169)

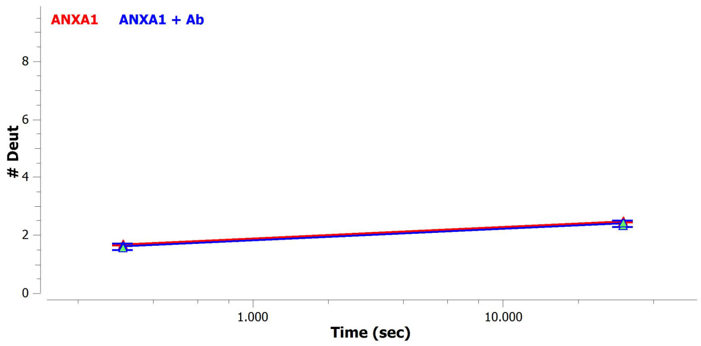


269-278: VKCATSKPAF (#170)

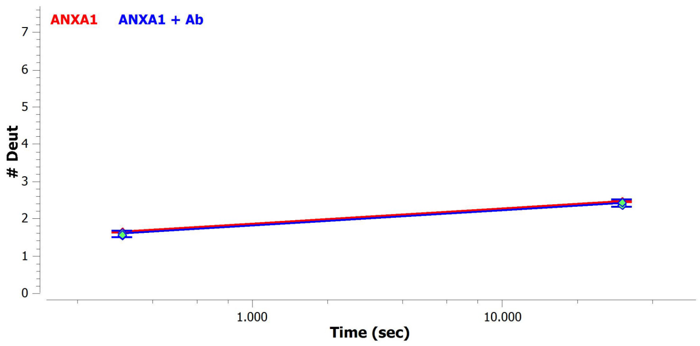

**270-278: KCATSKPAF (#171)**

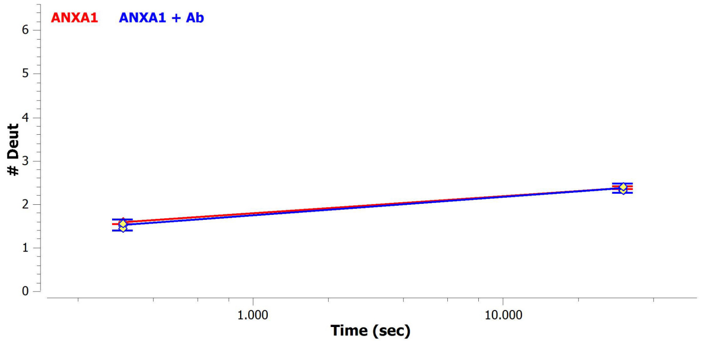


**279-297: FAEKLHQAMKGVGTRHKAL (#172)**

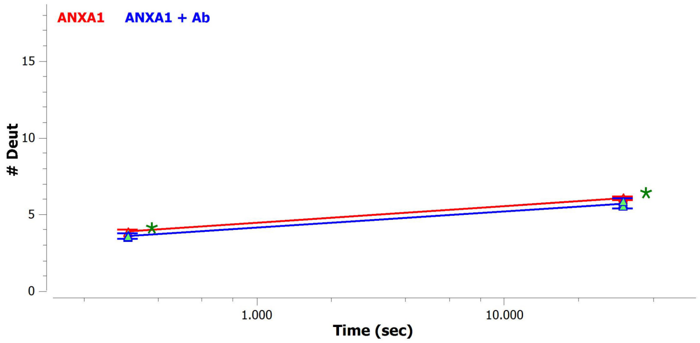


**279-300: FAEKLHQAMKGVGTRHKALIRI (#173)**

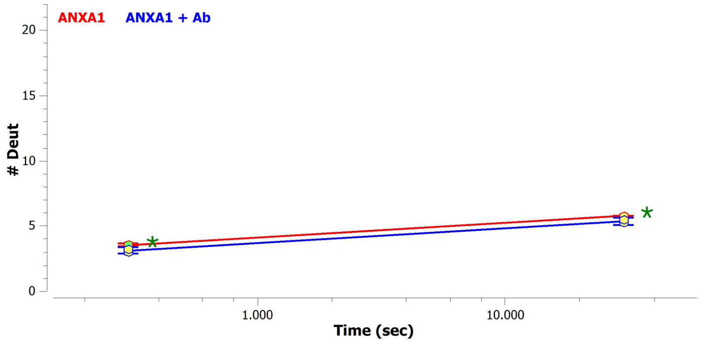


**279-309: FAEKLHQAMKGVGTRHKALIRIMVSRSEIDM (#174)**

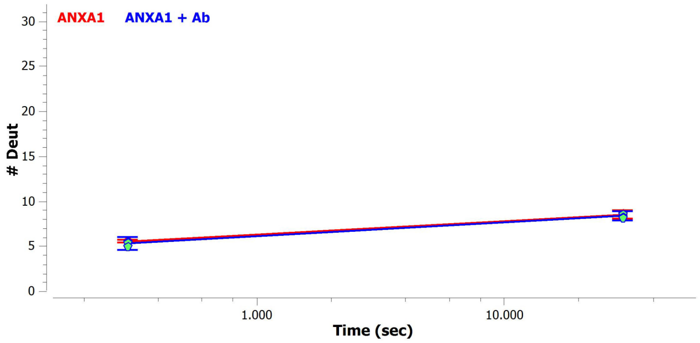


**280-297: AEKLHQAMKGVGTRHKAL (#175)**

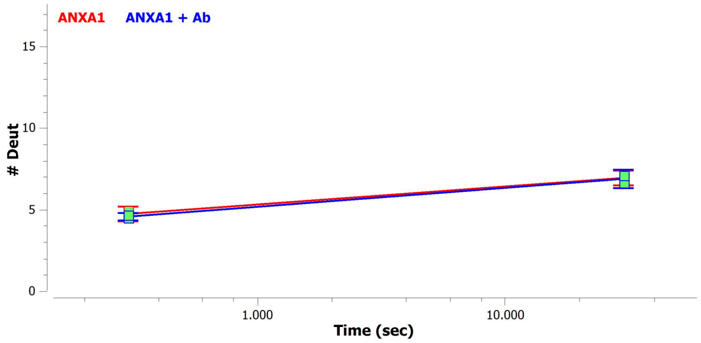


**298-303: IRIMVS (#176)**

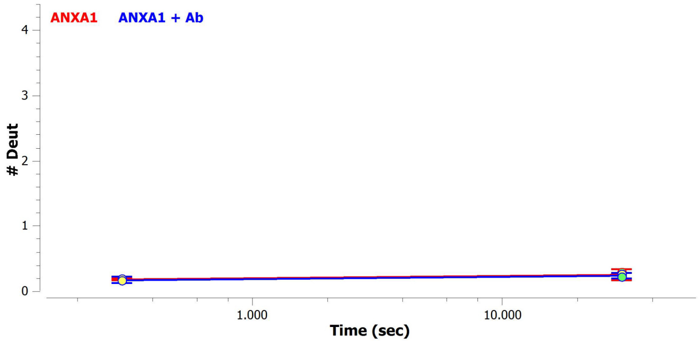


**298-305: IRIMVSRS (#177)**

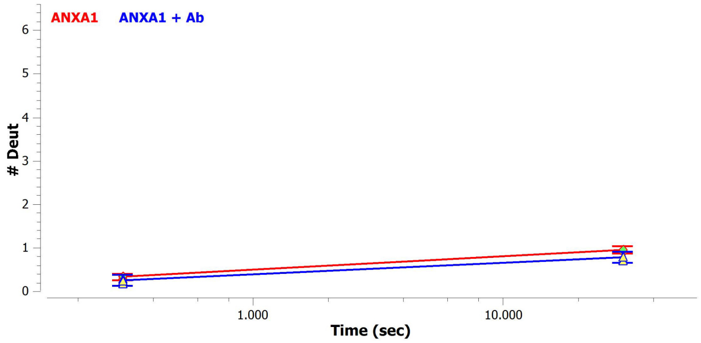


**298-306: IRIMVSRSE (#178)**

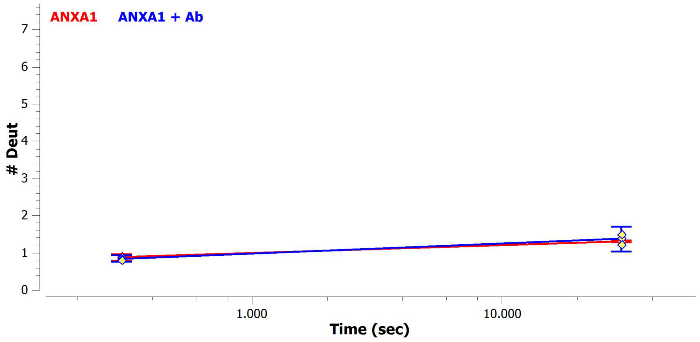


**298-309: IRIMVSRSEIDM (#179)**

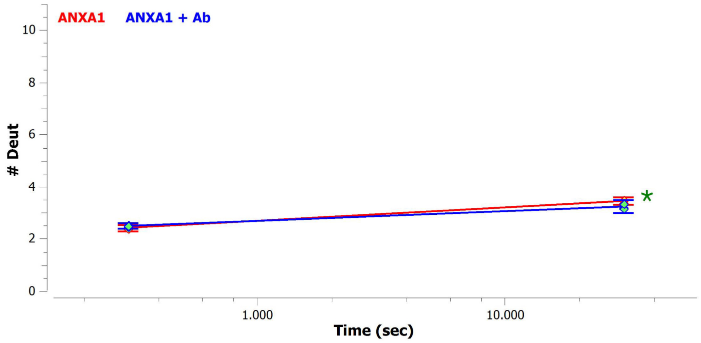


**298-311: IRIMVSRSEIDMND (#180)**

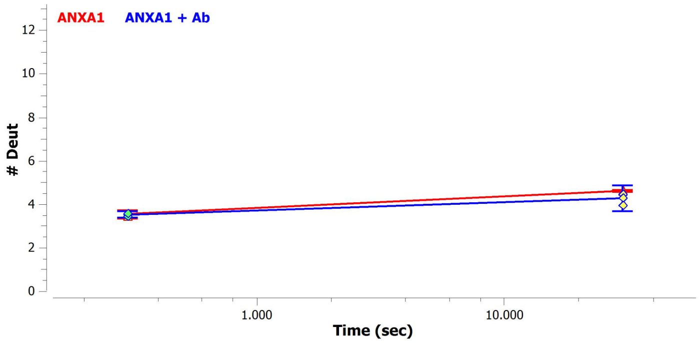

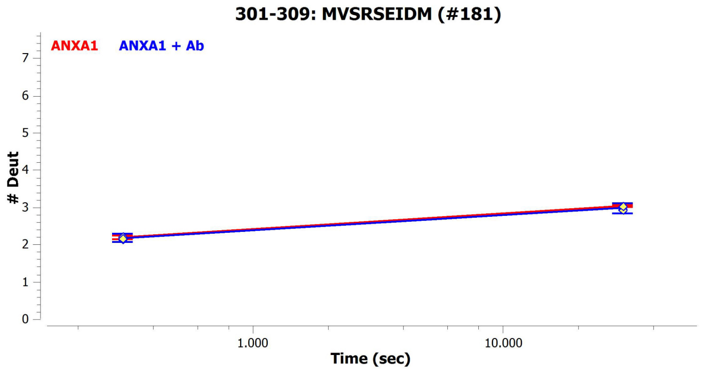
301-309: MVSRSEIDM (#181)
ANXA1
ANXA1 + Ab
# Deut
Time (sec)
1.000
10.000

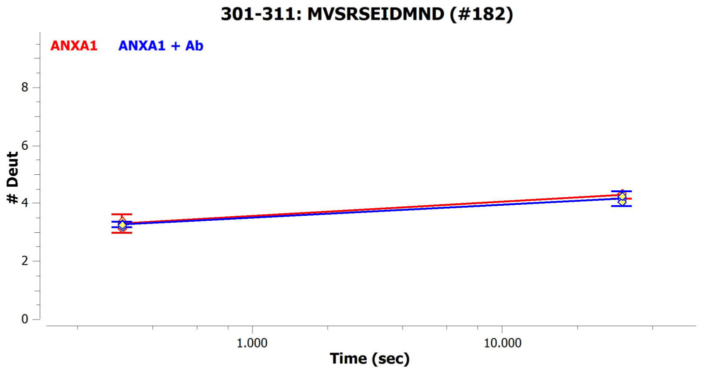
301-311: MVSRSEIDMND (#182)
ANXA1
ANXA1 + Ab
# Deut
Time (sec)
1.000
10.000

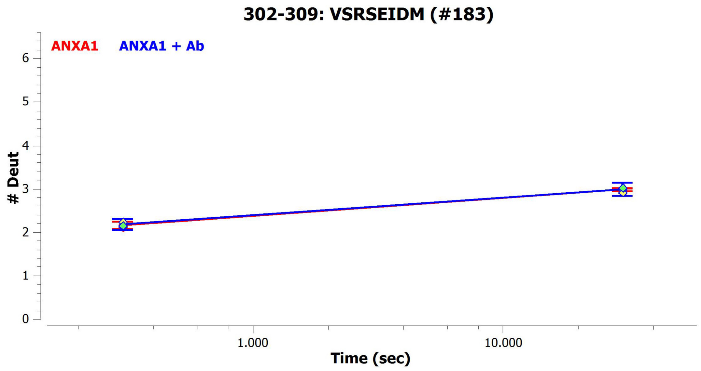
302-309: VSRSEIDM (#183)
ANXA1
ANXA1 + Ab
# Deut
Time (sec)
1.000
10.000

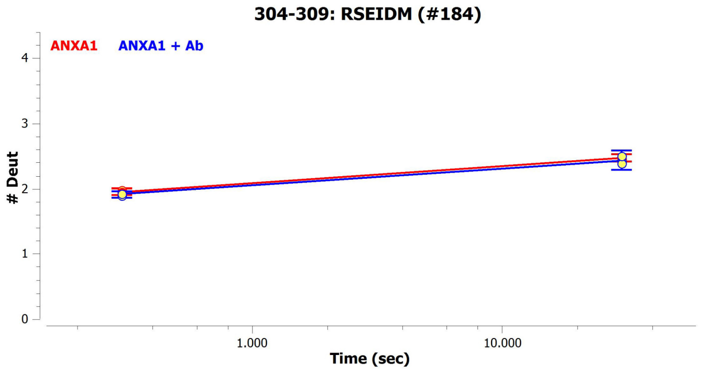
304-309: RSEIDM (#184)
ANXA1
ANXA1 + Ab
# Deut
Time (sec)
1.000
10.000

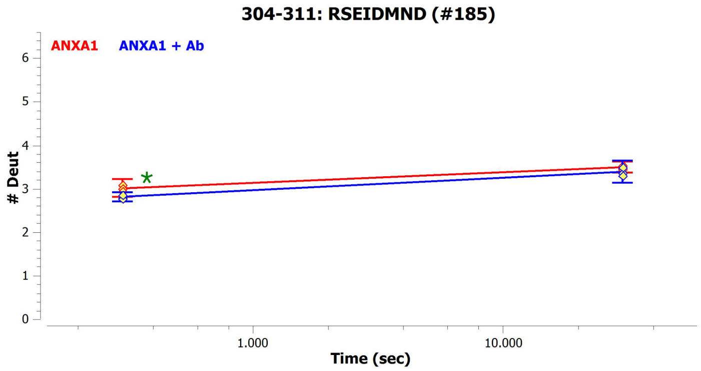
304-311: RSEIDMND (#185)
ANXA1
ANXA1 + Ab
# Deut
Time (sec)
1.000
10.000

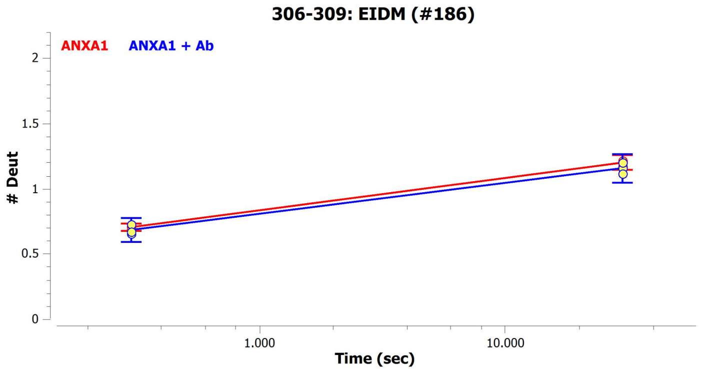
306-309: EIDM (#186)
ANXA1
ANXA1 + Ab
# Deut
Time (sec)
1.000
10.000

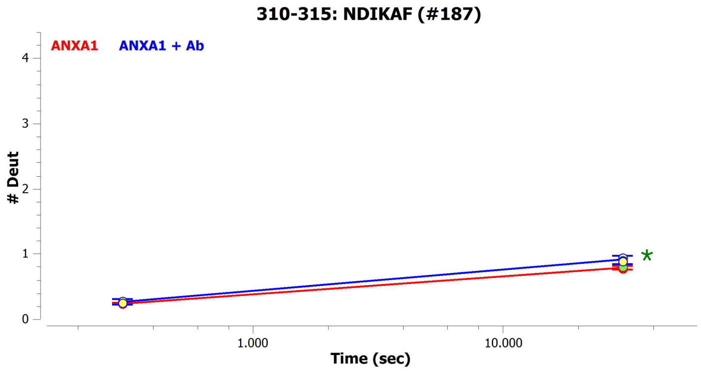
310-315: NDIKAF (#187)
ANXA1
ANXA1 + Ab
# Deut
Time (sec)
1.000
10.000

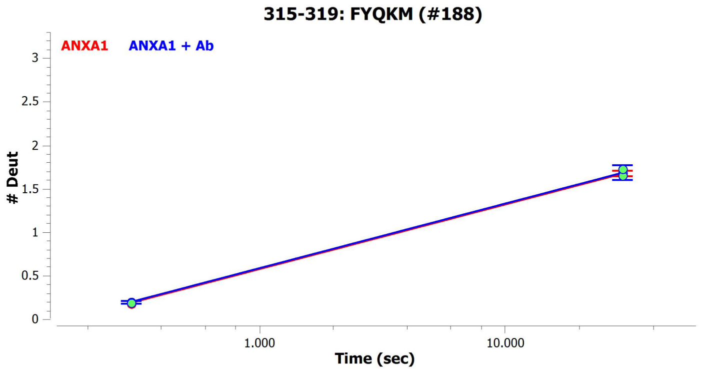
315-319: FYQKM (#188)
ANXA1
ANXA1 + Ab
# Deut
Time (sec)
1.000
10.000

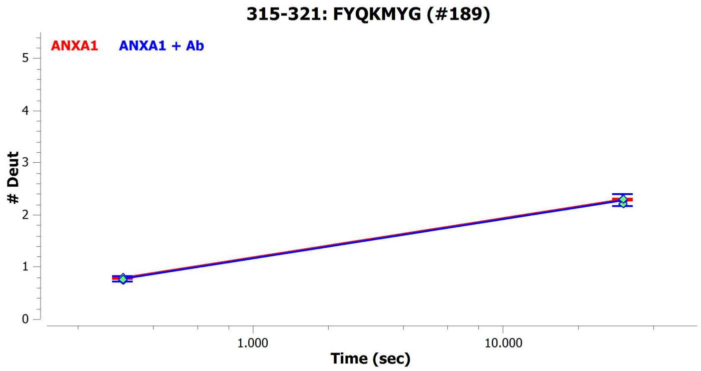
315-321: FYQKMYG (#189)
ANXA1
ANXA1 + Ab
# Deut
Time (sec)
1.000
10.000

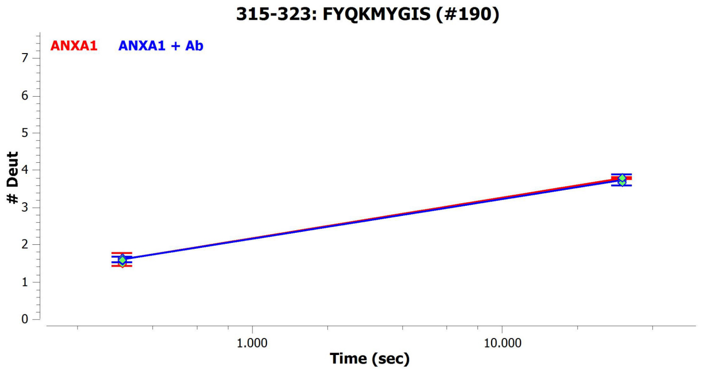
315-323: FYQKMYGIS (#190)
ANXA1
ANXA1 + Ab
# Deut
Time (sec)
1.000
10.000

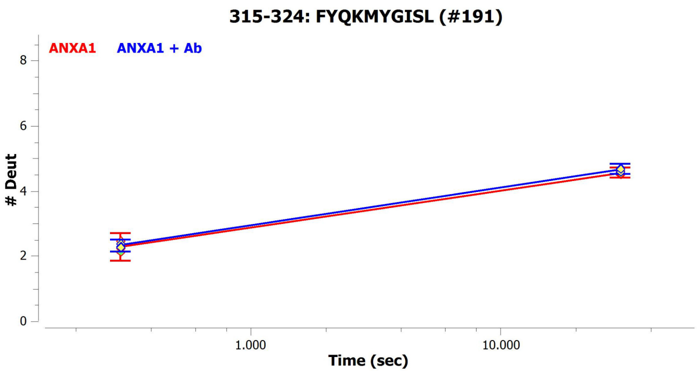
315-324: FYQKMYGISL (#191)
ANXA1
ANXA1 + Ab
# Deut
Time (sec)
1.000
10.000

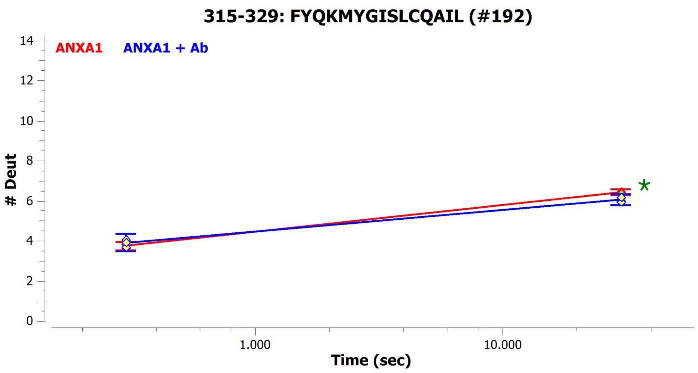
315-329: FYQKMYGISLCQAIL (#192)
ANXA1
ANXA1 + Ab
# Deut
Time (sec)
1.000
10.000

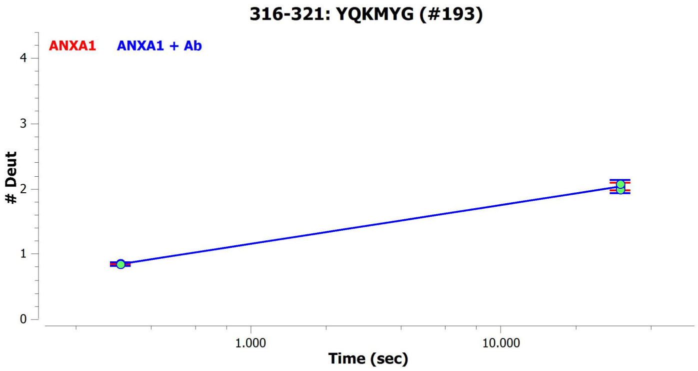
316-321: YQKMYG (#193)
ANXA1
ANXA1 + Ab
# Deut
Time (sec)
1.000
10.000

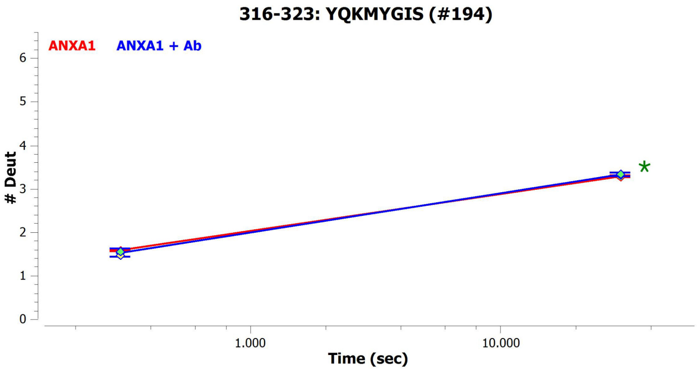
316-323: YQKMYGIS (#194)
ANXA1
ANXA1 + Ab
# Deut
Time (sec)
1.000
10.000

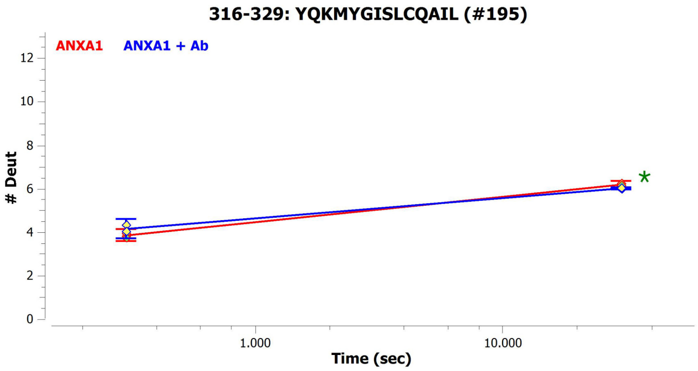
316-329: YQKMYGISLCQAIL (#195)
ANXA1
ANXA1 + Ab
# Deut
Time (sec)
1.000
10.000

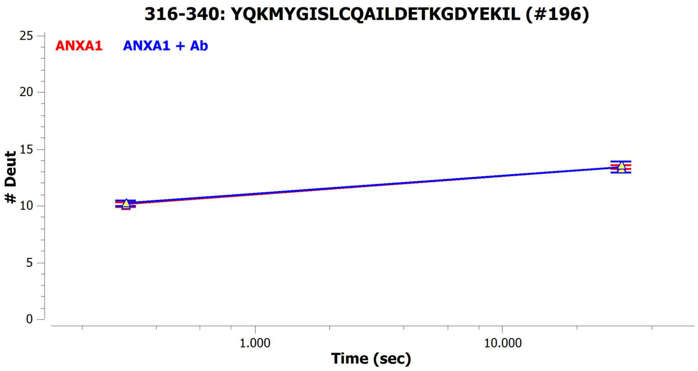
316-340: YQKMYGISLCQAILDETKGDYEKIL (#196)
ANXA1
ANXA1 + Ab
# Deut
Time (sec)
1.000
10.000

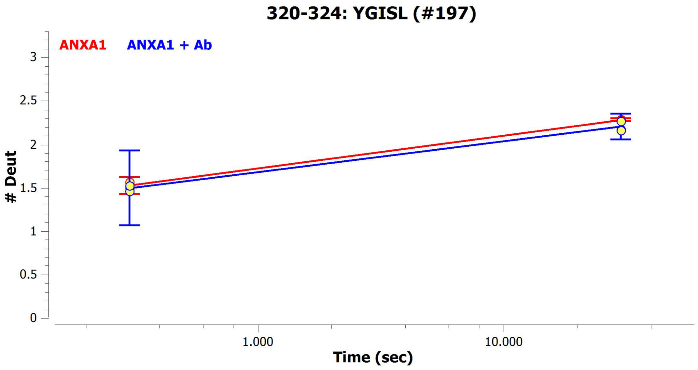
320-324: YGISL (#197)
ANXA1
ANXA1 + Ab
# Deut
Time (sec)
1.000
10.000

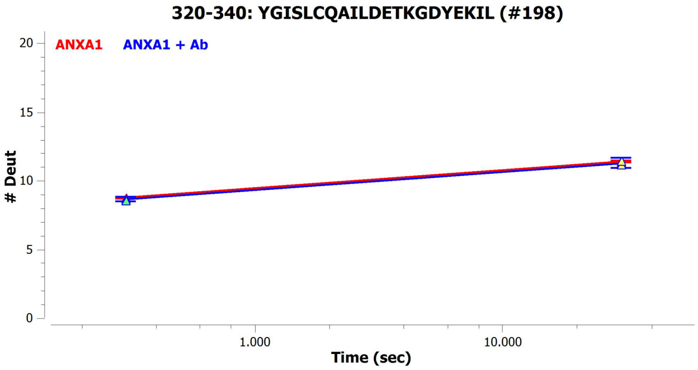
320-340: YGISLCQAILDETKGDYEKIL (#198)
ANXA1
ANXA1 + Ab
# Deut
Time (sec)
1.000
10.000

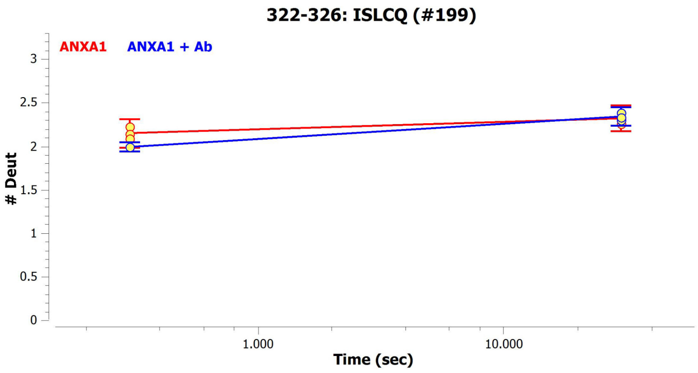
322-326: ISLCQ (#199)
ANXA1
ANXA1 + Ab
# Deut
Time (sec)
1.000
10.000

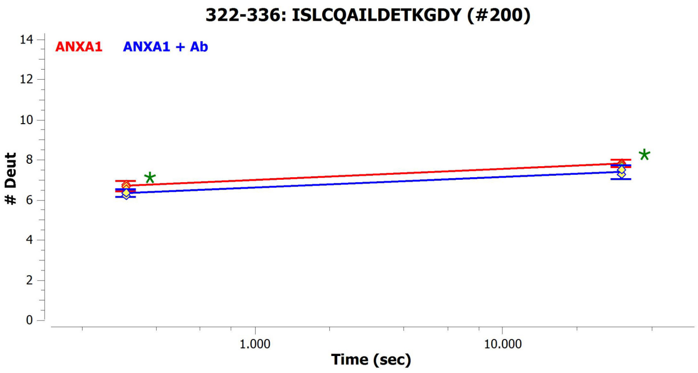
322-336: ISLCQAILDETKGDY (#200)
ANXA1
ANXA1 + Ab
# Deut
Time (sec)
1.000
10.000

**322-340: ISLCQAILDETKGDYEKIL (#201)**

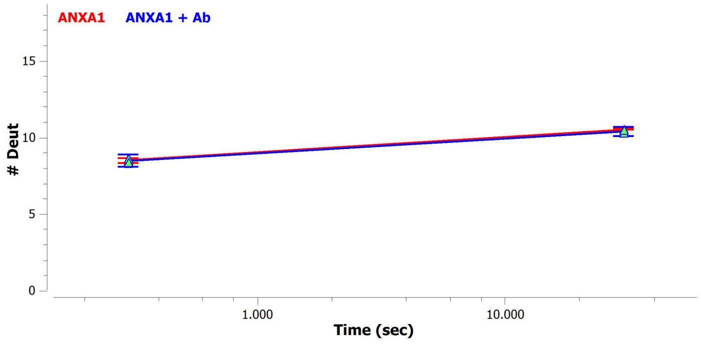


**322-347: ISLCQAILDETKGDYEKILVALCGGN (#202)**

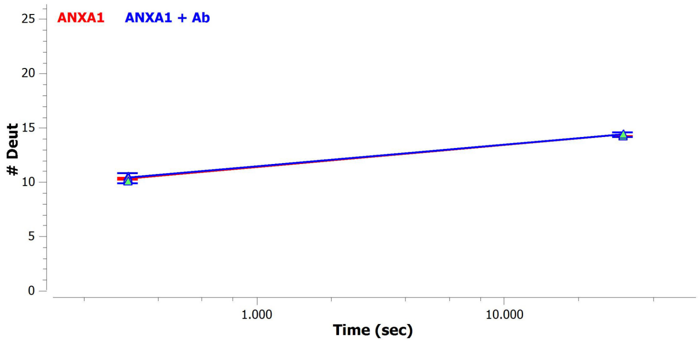


**324-329: LCQAIL (#203)**

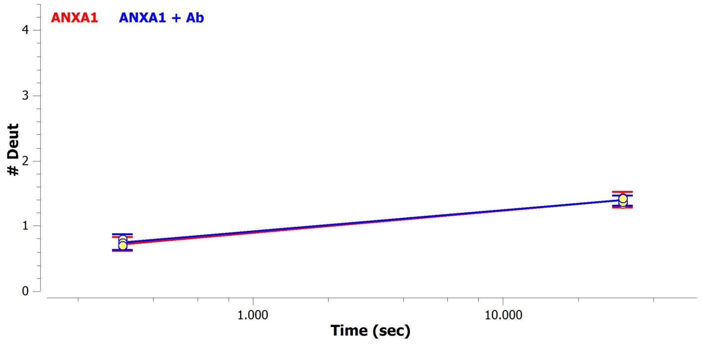


**325-329: CQAIL (#204)**

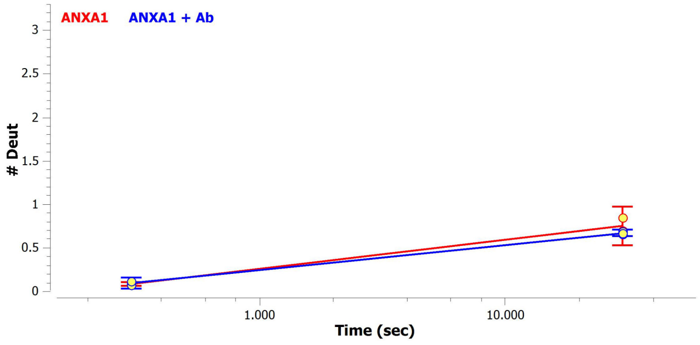


**330-340: DETKGDYEKIL (#205)**

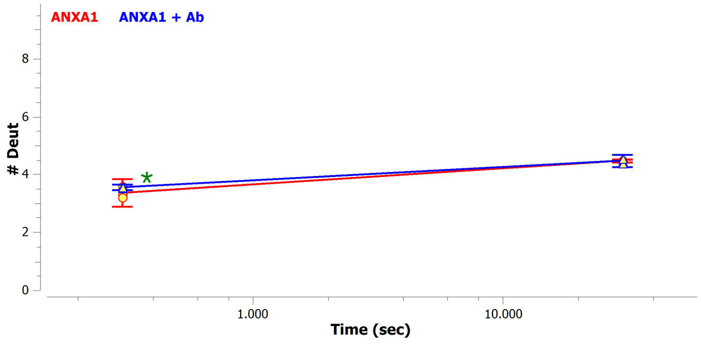


**332-340: TKGDYEKIL (#206)**

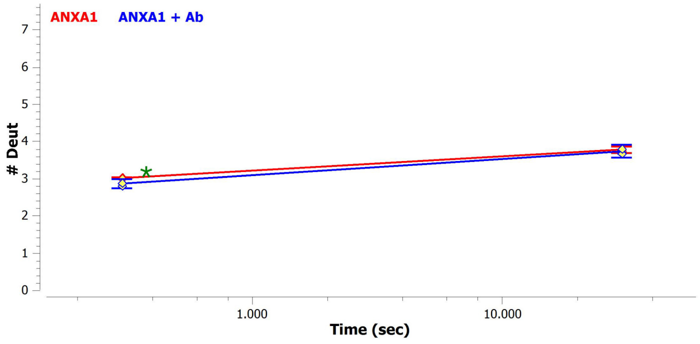


**341-347: VALCGGN (#207)**

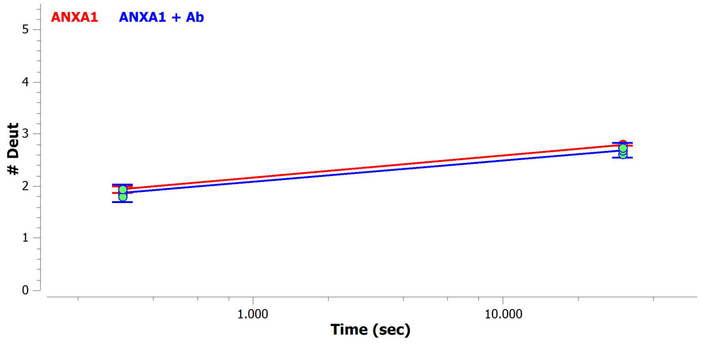

Figure S7: All peptide uptake plots of the **<u>bead based digest</u>**. used in this publication of the unbound state shown in red and the bound state shown in blue. Time points marked with a star show significant differences ($p<0.05$) between both states determined by students t-test. The Y-Axis scaled to 100% possible exchange of each peptide and the error bars are based on the 95% confidence level.
